# Size polymorphism and low sequence diversity in the locus encoding the *Plasmodium vivax* rhoptry neck protein 4 (PvRON4) in Colombian isolates

**DOI:** 10.1186/s12936-016-1563-4

**Published:** 2016-10-18

**Authors:** Sindy P. Buitrago, Diego Garzón-Ospina, Manuel A. Patarroyo

**Affiliations:** 1 Fundación Instituto de Inmunología de Colombia (FIDIC), Carrera 50 No. 26-20, Bogotá D.C., Colombia; 2 Microbiology Postgraduate Program, Universidad Nacional de Colombia, Bogotá D.C., Colombia; 3 School of Medicine and Health Sciences, Universidad del Rosario, Bogotá D.C., Colombia

**Keywords:** *Plasmodium vivax*, Rhoptry, Genetic diversity, Tandem repeat, *pvron4*, Natural selection, Functional restriction

## Abstract

**Background:**

Designing a vaccine against *Plasmodium vivax* has focused on selecting antigens involved in invasion mechanisms that must have domains with low polymorphism for avoiding allele-specific immune responses. The rhoptry neck protein 4 (RON4) forms part of the tight junction, which is essential in the invasion of hepatocytes and/or erythrocytes; however, little is known about this locus’ genetic diversity.

**Methods:**

DNA sequences from 73 Colombian clinical isolates from *pvron4* gene were analysed for characterizing their genetic diversity; *pvron4* haplotype number and distribution, as well as the evolutionary forces determining diversity pattern, were assessed by population genetics and molecular evolutionary approaches.

**Results:**

*ron4* has low genetic diversity in *P. vivax* at sequence level; however, a variable amount of tandem repeats at the N-terminal region leads to extensive size polymorphism. This region seems to be exposed to the immune system. The central region has a putative esterase/lipase domain which, like the protein’s C-terminal fragment, is highly conserved at intra- and inter-species level. Both regions are under purifying selection.

**Conclusions:**

*pvron4* is the locus having the lowest genetic diversity described to date for *P. vivax*. The repeat regions in the N-terminal region could be associated with immune evasion mechanisms while the central region and the C-terminal region seem to be under functional or structural constraint. Bearing such results in mind, the PvRON4 central and/or C-terminal portions represent promising candidates when designing a subunit-based vaccine as they are aimed at avoiding an allele-specific immune response, which might limit vaccine efficacy.

**Electronic supplementary material:**

The online version of this article (doi:10.1186/s12936-016-1563-4) contains supplementary material, which is available to authorized users.

## Background

Malaria is the parasitic disease having the greatest impact on public health [1]. It is caused by different species from the *Plasmodium* genus, these being widely distributed throughout the world’s tropical and sub-tropical regions [2]. These parasites cause 140–300 million clinical cases and more than half a million deaths annually [3, 4]. *Plasmodium falciparum* is considered the most lethal species, mainly affecting vulnerable populations in sub-Saharan Africa [4]. Even though efforts were initially concentrated on controlling this species, reports of ever-increasingly severe cases caused by *Plasmodium vivax* [5] and the appearance of drug-resistant strains during the last few years [6, 7] has made this species a growing public health problem, affecting more than a third of the world’s population, having high prevalence in Asia and South and Central America [3, 7, 8].

Designing an anti-malarial vaccine against *P. vivax* (as for *P. falciparum*) has been focused on blocking parasite-host interactions during different parasitic stages, especially during the blood phase responsible for the disease’s clinical manifestations [9, 10]. A large amount of *P. vivax* antigens have been characterized to date [10, 11], however, their genetic diversity should be assessed for selecting the best antigens for vaccine development [10, 12]. Highly polymorphic antigens can provoke allele-specific immune responses leading to protection having low efficacy after vaccination. On the contrary, those having limited diversity are attractive targets for being evaluated as candidates as they avoid an allele-specific immune response [13].

Most antigens characterized to date have been merozoite proteins [10, 11], including the microneme AMA1 protein and rhoptry neck (RONs) proteins. The interaction between these proteins (specifically AMA1–RON2) has been well described in *Toxoplasma gondii* and *P. falciparum,* these being the structural basis for the tight junction (TJ), a connective ring through which a parasite enters a host cell [14–18].

The RON protein complex (characterized in *P. falciparum*) consists of RON2, RON4 and RON5 proteins [14, 17, 19]. Even though the mechanisms regarding function and interaction between the complex’s proteins are not clear, they are considered important targets for blocking invasion. Various studies have highlighted the potential of AMA1 and RON2 as vaccine candidates, however, current knowledge concerning the other RONs is deficient. Co-localization studies and invasion models described for *Plasmodium* spp and *T. gondii* have led to establishing RON4’s convincing participation in the TJ [15, 17, 20, 21]. Likewise, its expression in the parasite’s invasive forms [21–23], the ability to provoke an immune response in natural malarial infections [23] and the protein’s conserved nature, specifically towards the C-terminal (inferred by comparative analysis between *Pf*RON4 and *Tg*RON4 amino acid sequences) [24] suggest that this protein plays an important role for the parasite and could thus be evaluated as vaccine candidate.

The *P. falciparum* RON4 orthologue has recently been characterized in the *P. vivax* VCG-I strain (Vivax Colombia Guaviare-I) [22]. PvRON4 (*P. vivax* RON4) is encoded by a gene having around 2657 bp in this species, expressed during the last hours of the intra-erythrocyte cycle and secreted from the rhoptry neck. This consists of signal peptide sequence, a low complexity domain formed by two types of tandem repeats, a double spiral alpha helix domain and five conserved cysteines towards the C-terminal [22]; the latter region seems to be highly conserved among *P. vivax* and parasite species infecting monkeys [25].

Bearing RON4’s potential participation in invasion in mind and given that parasite antigen genetic diversity is an obstacle for designing a completely effective vaccine against *P. vivax*, this study was thus aimed at using Colombian clinical isolates for evaluating *pvron4* locus genetic diversity and the evolutionary mechanisms determining its variation pattern.

## Methods

### Sample collection

*Plasmodium vivax* genomic DNA was obtained from 73 clinical isolates collected from 2007 to 2015 (2007: 10, 2008: 12, 2010: 18, and 2015: 33 samples). These came from Colombia’s Pacific coast region (Chocó and Nariño departments), Urabá/lower Cauca/southern Córdoba (Córdoba and Antioquia departments) and the Orinoquia-Amazonia region (Amazonas and Guainía departments), representing the three regions having the greatest transmissibility in Colombia [26]. More than 360,000 cases of *P. vivax* infection were recorded between 2007 and 2015, more than 14 % of them regarding Colombia’s Pacific coastal region, Urabá/lower Cauca/southern Córdoba 62 % while 7.5 % of *P. vivax* cases were recorded in the Orinoquia-Amazonia region. Malaria symptomatic patients (living in the regions described above) were diagnosed with *P. vivax* infection by microscopy and then invited to donate 5 mL of venous blood. Some Amazonia samples were collected and diagnosed, as has been reported elsewhere [27]. Male and female patients aged 16–64 years were invited to participate in the study. DNA was extracted and stored at −20 °C before being processed and were genotyped by PCR-RFLP from the *pvmsp*-*3α* gene.

### Amplifying, cloning and sequencing the *pvron4* locus

A set of primers was designed to amplify and clone *pvron4* based on Sal-I genomic sequence (GenBank accession number AAKM01000005.1), sequences being as follows: *pvron4* dir 5′ CACAGTGCAACCATGTCTCG 3′ (20 bp) and *pvron4* rev 5′ GCAAGCTAATTTCACAAGTCTTC 3′ (23 bp) primers. Touchdown-PCR was used for amplification using the KAPA-HiFi HotStart Readymix enzyme (Kapa Biosystems) in 25 μL reactions using VCG-I strain genomic DNA as positive control. Thermal conditions were as follows: a 5 min denaturing step at 95 °C, 10 cycles consisting of 20 s at 98 °C, 15 s at 68 °C (temperature was reduced by one degree per cycle) and 1 min at 72 °C, followed by 35 cycles of 20 s at 98 °C, 15 s at 60 °C, 1 min at 72 °C and a final 5-min extension at 72 °C. PCR products were purified using an UltraClean PCR Clean-up purification kit (MOBIO).

The amplicons were ligated in pGEM T-easy cloning vector and then used for transforming *Escherichia coli* JM109 strain cells. Recombinant bacteria were selected by the alpha complementation method and their growth ability in the presence of ampicillin. These were confirmed by PCR using MangoTaq DNA polymerase and internal primers for the gene (intdir: 5′ TGTGGGTGGCGAGAGTG 3′ (17 bp), and intrev: 5′ ATATTTCCATTGCTGTACTAGG 3′ (22 bp), designed on Sal-I genomic sequence) using the following thermal conditions a 5 min denaturing step at 95 °C, 35 cycles of 20 s at 98 °C, 15 s at 60 °C, 1 min at 72 °C and a final 5-min extension at 72 °C. The plasmid from the two recombinant colonies per sample was extracted using an UltraClean 6 Minute Mini Plasmid Prep kit (MOBIO) and sent to be sequenced using a BigDye Terminator kit (MACROGEN, Seoul, South Korea), with universal primers SP6 Promoter Primer (Cat.# Q5011), T7 Promoter Primer (Cat.# Q5021) [28] and a set of internal primers (*pvron4dseq*: 5′ CACTAGAAAAGCTAAACATAAACC 3′ (24 bp), and *pvron4rseq*: 5′ ACTCCAATGGTCCTCAAC 3′ (18 bp) designed on the Sal-I genomic sequence) for sequencing.

### Statistical analysis of *pvron4* sequences

The electropherograms obtained by sequencing were assembled using CLC DNA workbench v.3 software (CLC bio, Cambridge, MA, USA). The 73 consensus sequences obtained in this study (Additional file 1), 7 reference sequences (from the Salvador-I (Sal-I, GenBank: XM_001615228.1), Mauritania-I (GenBank: AFNI01000694.1), India-VII (GenBank: AFBK01001155.1), Brazil-I (GenBank: AFMK01001544.1/AFMK01001546.1), North Korea (GenBank: AFNJ01001110.1), ctg (GenBank: AAKM01000005) and P01 (GeneDB: PVP01_0916600.1) strains) and 13 orthologous sequences (from *Plasmodium cynomolgi* (GeneBank: BAEJ01000746.1), *Plasmodium knowlesi* (GeneBank: NC_011910.1), *Plasmodium inui* (GeneBank: AMYR01000790.1/AMYR01000791.1), *Plasmodium fragile* (GeneBank: NW_012192637.1), *Plasmodium coatneyi* (GeneBank: CM002860.1), *Plasmodium reichenowi* (GeneBank: LVLA01000012.1), *P. falciparum* (GeneBank: XM_001347803.2), *Plasmodium bergei* (GeneBank: CAAI01002287.1), *Plasmodium yoelii* (GeneBank: AABL01000590.1), *Plasmodium chabaudi* (GeneBank: CAAJ01004050.1), *Plasmodium vinckei* (GeneBank: AMYS01000107.1), *Plasmodium gaboni* (GeneBank: LVLB01000012.1), and *Plasmodium gallinaceum* (Sanger Institute: gal28a.d000001405.Contig1/gal28a.d000000110.Contig1) were used for obtaining the deduced amino acid sequence used for multiple alignment with the MUSCLE algorithm [29]. Such alignment was manually edited to ensure correct repeat region alignment and then used for inferring DNA alignment by Pal2Nal software [30]. The T-REKS algorithm was used for identifying repeat regions [31].

DnaSP v.5 software [32] was used for calculating genetic diversity regarding Colombian sequences and *P. vivax* reference sequences alignment using estimators based on single nucleotide polymorphism (SNP) and sequence length (InDels). Tajima [33], Fu and Li [34], Fu [35], Fay and Wu [36] tests were used for evaluating deviations from the neutral model of molecular evolution, bearing coalescence simulations in mind for obtaining confidence intervals and their statistical significance. The repeat regions or those having gaps were not taken into account for analysis.

The Nei-Gojobori modified method [37] with MEGA v.6 software [38] was used for calculating the average number of synonymous substitutions per synonymous site (d_S_) and the average number of non-synonymous substitutions per non-synonymous site (d_N_) at intra-species level. The average amount of synonymous divergences per synonymous site (K_S_) and the average amount of non-synonymous divergences per non-synonymous site (K_N_) were calculated by modified Nei-Gojobori method with Jukes-Cantor correction [39] for determining natural selection signals throughout *Plasmodium* spp evolutionary history (using the *P. vivax* sequences, together with phylogenetically close parasites’ orthologous sequences). The differences between intra- and inter-species substitution rates were determined by Fisher’s exact test (recommended when the amount of synonymous and/or no-synonymous substitutions is fewer than ten) or the Z-test incorporated in MEGA software v6. Additionally, the McDonald–Kreitman (MK) test [40] with Jukes-Cantor correction was used for evaluating neutrality deviations using the Standard & Generalized McDonald–Kreitman Test web server [41, 42].

A sliding window was used for analysing evolutionary rate (ω = d_N_/d_S_ and/or K_N_/K_S_) by evaluating the effect of selection throughout the gene. Individual sites under selection were identified by calculating synonymous and non-synonymous substitution rates per codon using SLAC, FEL, REL, IFEL [43], MEME [44], and FUBAR methods [45] in the Datamonkey online server [46]. Repeat regions or those having gaps were not taken into account for this analysis.

The random effects likelihood (REL)-branch-site method [47] was used for evaluating the existence of lineages under episodic diversifying selection in *Plasmodium* for the *ron4* locus. The MUSCLE algorithm was used for aligning 14 orthologous protein sequences from different species from the genus; this was then used for inferring the best evolutionary model using MEGA software. Phylogeny was then inferred by using the maximum likelihood method with the JFF + G + F model. This is used as reference for analysing lineage-specific positive selection with the REL-branch-site method in the HyPhy package [48], using a DNA alignment inferred by Pal2Nal from aligning amino acids.

### Effective number of codons

ENCprime [49] and DnaSP software were used for estimating the effective number of codons (ENC). This is a measurement of selective pressure at translational level, leading to protein function loss or gain [49]. This test compares the use of each codon versus a null distribution (uniform use of synonymous codons). ENC values close to 61 indicate that all synonymous codons for each amino acid are used equitably, while values close to 0 suggest bias or preferential codon use [50]. Statistical significance could be affected by gene length or recombination [50]. The codon bias index (CBI) was thus used, which takes values ranging from 0 (uniform use of synonymous codons) to 1 (maximum codon bias) [51].

### Linkage disequilibrium and recombination

Linkage disequilibrium (LD) was evaluated by calculating the Z_nS_ estimator [52]. A linear regression between this and the nucleotide distance was performed to see whether intragenic recombination occurred in *pvron4*. Recombination was also evaluated by ZZ estimator [53], the minimum number of recombination events (Rm) [54], the GARD algorithm [55] and the RDP v.3 software [56].

### *pvron4* locus differentiation and population genetic structure

The degree of genetic differentiation (or inter-population heterogeneity) in the *pvron4* locus among Colombian *P. vivax* malaria-endemic regions was estimated by analysis of molecular variance (AMOVA) and Wright’s fixating index (F_ST_), using the Arlequin v.3.1 software [57]. NETWORK v.5 software was used for constructing a median joining network for evaluating possible mutational pathways giving rise to *pvron4* haplotypes, their distribution and frequencies. This method expresses multiple plausible evolutionary pathways as cycles, bound by vectors interpreted as extinct ancestral sequences [58].

### Predicting *pvron4* putative domains and antigenic potential

The Blastp algorithm from the NCBI web server was used for identifying putative domains in PvRON4 using the Sal-I sequence as reference. The B-cell epitope prediction tool available at the immune epitope database (IEDB) server was used for evaluating PvRON4s antigenic potential regarding its antigenicity [59], its hydrophobicity [60], protein solvent availability [61] and its potential linear B-cell epitopes [62]. These tests were done with two PvRON4 haplotypes differentiated by the amount of repeats: haplotype 6 (one copy of GEHGEHAEHGE) and haplotype 17 (seven copies of the repeat), to evaluate the effect of repeat number on protein antigenic behavior.

## Results

### *pvron4* locus genetic diversity

**Fig. 1:**
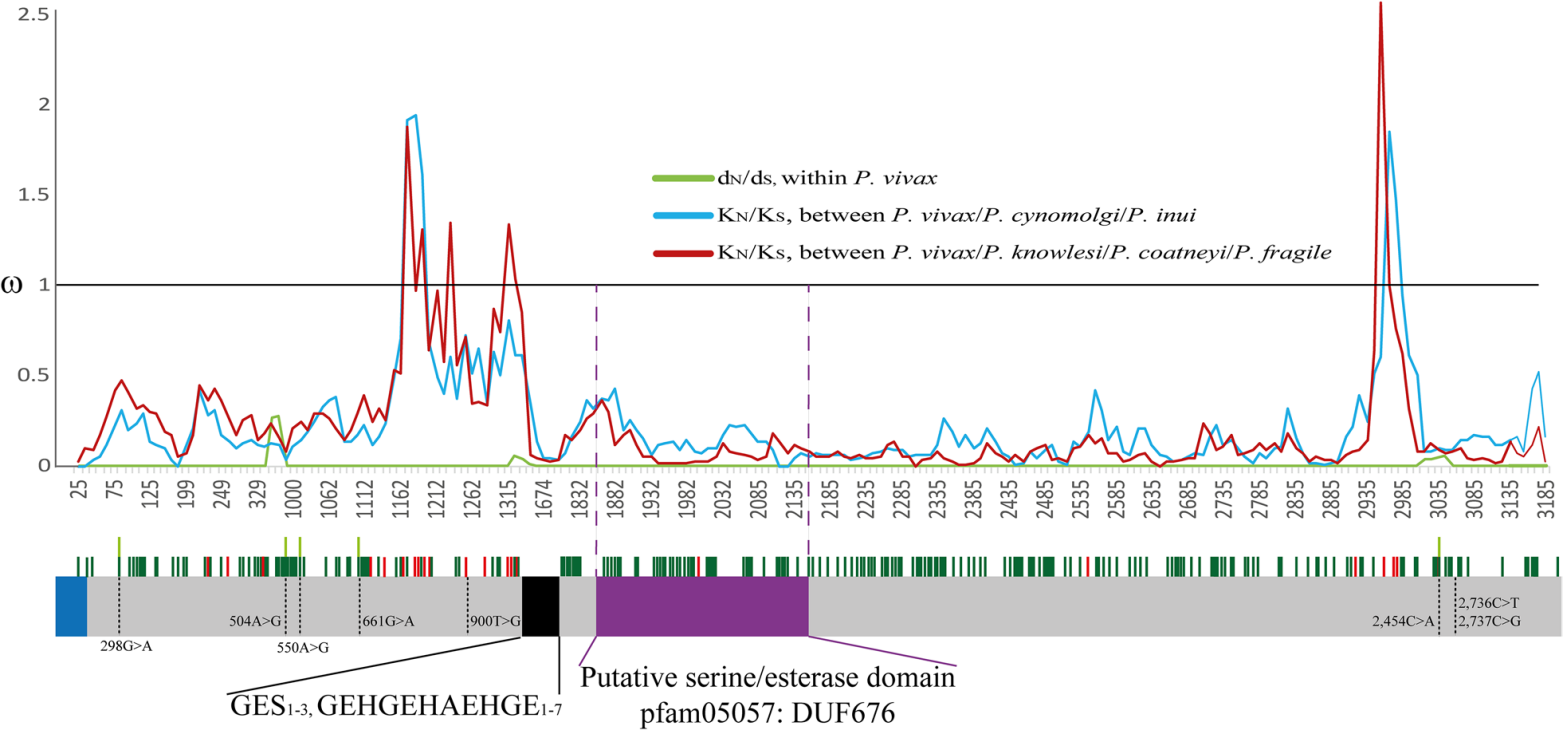
Representation of *pvron4* encoding DNA sequence and sliding window for ω rate. *ron4* ω values (d_N_/d_S_) within *P. vivax* are shown in *green* throughout. Likewise, ω divergence rates (K_N_/K_S_) between the species infecting primates and *P. vivax* are shown in *blue* (for the species evolutionarily closest to *P. vivax*) and *red* (most distant). A representation of the *ron4* gene is given below the sliding window indicating signal peptide (*blue*), the repeat region (*black*) and the putative esterase/lipase domain (*purple*). The *P. vivax* SNP positions are shown as* discontinue lines*, their numbering is based on Additional file 2 alignment. The sites under purifying selection within species are represented by *light green lines* and *dark green* between species while positively selected sites between species are shown by *red lines*

Seventy-three sequences from the *pvron4* locus obtained from the *P. vivax* Colombian population (24 from Orinoquia-Amazonia, 21 from the Pacific coast and 28 from Urabá/lower-Cauca/southern Córdoba) were analysed. *pvron4* was initially annotated from the VCG-I strain as being a 2657 bp gene [22], however, the locus analysed from Colombian samples had a variation in length due to two tandem repeats located towards the gene’s 5′-end (Fig. 1). These repeats consisted of copies of the GTGGCGAGA nucleotide sequence encoding GES amino acids (repeated one to three times) and a longer sequence CGGAGAGCACGGTGAACACGCTGAACATGGGGAGCA encoding the GEHGEHAEHGE peptide (repeated one to seven times).

Few SNPs were found. Regarding the 2542 sites analysed concerning the Colombian sequences and the reference ones, the number of SNPs varied from five to eight (Table 1). The genetic diversity estimators gave low values (θ_w_ = 4.7 × 10^−4^ and π = 4.1 × 10^−4^) in the *P. vivax* Colombian population. Such values remained constant when comparing the Colombian sequences to the reference ones (Table 1). Likewise, the total amount of *pvron4* haplotypes identified and haplotype diversity were low (Table 1), however, 15 haplotypes in the Colombian population (21 when Colombian and reference sequences are analysed together) and high diversity estimator values (Table 1) were found when analysing insertions/deletions (InDels) in *pvron4.* Bearing the SNPs and InDels between the *pvron4* reference sequence and Colombian sequences in mind, 32 haplotypes were identified (Additional file 2).

**Table 1:** *pvron4* genetic diversity estimators calculated from single nucleotide polymorphism and sequence length polymorphism

Single nucleotide polymorphism									Sequence length polymorphism				
n	Sites	Ss	S	Ps	H	Hd	θ_w_	π	Sites	No InDels	H	Hd	π
*Colombian and reference isolates*													
80	2542	8	3	5	11	0.67	7.6 × 10^−4^	4.3 × 10^−4^	408	21	21	0.82	9.9 × 10^−4^
*Colombian isolates*													
73	2464	5	0	5	8	0.65	4.7 × 10^−4^	4.1 × 10^−4^	289	15	15	0.78	8.0 × 10^−4^

Genetic diversity estimators were calculated using the reference sequences obtained from databases together with Colombian isolates as well as for just Colombian isolates’ sequences

*n* number of isolates analysed, *sites* total of sites analysed excluding gaps, *Ss* number of segregating sites, *S* number of singleton sites, *Ps* number of informative-parsimonious sites, *H* number of haplotypes, *Hd* haplotype diversity, *θ*_*w*_ Watterson estimator, *π* nucleotide diversity per site

### Evaluating the effect of selection on the *pvron4* locus

No statistically significant values were found for *pvron4* when using the Tajima, Fu and Li, Fay and Wu and Fu estimators (Table 2). Likewise, the MK test did not reveal any deviations regarding neutrality (Table 3), however, the d_N_ − d_S_ difference (Table 3) showed that the synonymous substitution rate was higher than the non-synonymous substitution rate (p = 0.000, Fisher’s exact test), suggesting that *pvron4* was under purifying selection. The sliding windows led to ω < 1 values being observed throughout the gene (Fig. 1).

**Table 2:** Neutrality, linkage disequilibrium and recombination tests for the *pvron4* gene in the *Plasmodium vivax* Colombian population

N	Gene	Tajima	Fu and Li		Fay and Wu’s H	Fu´s Fs	Z_nS_	ZZ	RM
		D	D*	F*					
*Colombian and reference isolates*									
80	2578	NP	NP	NP	NP	NP	0.154^τ^	−0.002	0
*Colombian isolates*									
73	2578	−0.037	−0.035	−0.020	−0.039	−0.028	0.163^τ^	−0.002	0

No statistically significant values were found in neutrality tests

*Z*_*nS*_ average of R^2 for all comparison pairs, *ZZ: Z*_*nS*_ *−* *Za* difference, *Rm* minimum amount of recombination events, *NP* not performed, since not all sequences belonged to the same population

^τ^ p < 0.05

**Table 3:** Difference between d_N_ _−_ d_S_, K_N_ _−_ K_S_ and the neutral index from MK test

*P. vivax*	*P. vivax/Plasmodium* ssp						
	*P. knowlesi*	*P. inui*	*P. coatneyi*	*P. cynomolgi*	*P. fragile*	*P.cyn/P.inu*	*P.kno/P.coa/P.fra*
d_N_ _−_ d_S_	*K*_*N*_ _−_ *K*_*S*_						
−0.002*	−0.011^β^	−0.006^β^	−0.006^β^	−0.009^β^	−0.010^β^	−0.015^β^	−0.025^β^
	*NI*						
	0.696	0.597	0.857	1.133	0.919		

Non-synonymous substitution rate (d_N_) and synonymous substitution rate (d_S_) within *P. vivax*. Non-synonymous (K_N_) and synonymous (K_S_) divergence between *P. vivax* and phylogenetically close species. Neutrality index (NI) estimated by McDonald–Kreitman test using Jukes Cantor correction

*P.cyn P. cynomolgi*, *P.inu P. inui*, *P.kno P. knowlesi*, *P.coa P. coatneyi*, *P.fra P. fragile*

* p < 0.01

^β^ p < 0.001

When comparing phylogenetically related species, the sliding window gave ω < 1 values, with few regions having ω ≥ 1 (positions 1172–1325, 2955–2975 and in position 2955; Fig. 1). The K_N_ − K_S_ difference (Table 3) gave negative values suggesting that purifying selection has played an important role in this locus' evolutionary history in the genus *Plasmodium*. On the other hand, four negatively selected sites (28, 112, 149, 643; Fig. 1) were found throughout the gene in *P. vivax* when calculating selection per codon based on maximum probability methods (SCAL, FEL, IFEL, REL, and FUBAR) and the Bayesian method (MEME), while no sites were found to be under positive selection (Fig. 1). These methods revealed 162 sites under negative selection and 21 under positive selection between species (Fig. 1).

**Fig. 2:**
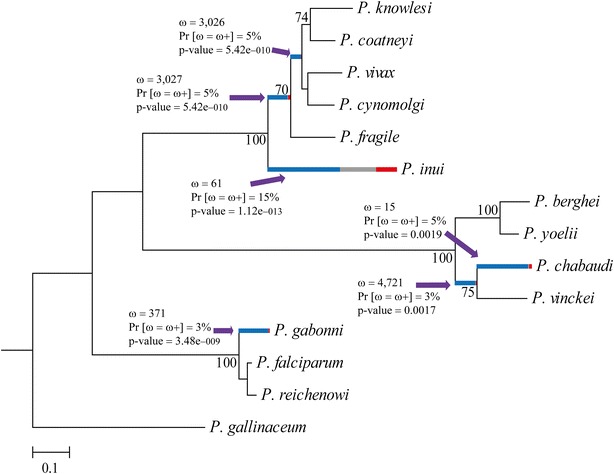
Lineages subject to episodic diversifying selection in *ron4*. The branches (lineages) under episodic positive selection were identified by the Branch-site REL method. The figure shows the ω^+^ values, the percentage of sites (Pr [ω = ω+]) and p values. The shade of each *colour* on branches indicates strength of selection (*red* shows ω^+^ > 15, *blue* ω^−^ ≤ 1 and *grey* ω = 1). The size of each *colour* represents the percentage of sites in the corresponding class. Branches have been classified as undergoing episodic diversifying selection by the p value corrected for multiple testing using the Holm-Bonferroni method at p < 0.05

Phylogeny was inferred from orthologous sequences from 14 *Plasmodium* species. This was used as reference framework for the REL-branch-site test. This method led to finding six branches (lineages) having evidence of episodic selection (Fig. 2). Three were ancestral branches (internal) while the other three (external) had given rise to *Plasmodium inui*, *Plasmodium chabaudi* and *Plasmodium gaboni*. The MEME method revealed codons under diversifying episodic selection.

### Effective number of codons

Given the high conservation of the *pvron4* locus, deviation regarding the effective use of codons was evaluated as a means of selection at translational level. The ENC for *pvron4* estimated by ENCprime (N'c = 53, scaled *X* ^2^ = 0.103) and DnaSP (ENC = 55.4, scaled *X* ^2^ = 0.159) gave values close to 61, with a CBI value of 0.162, suggesting that there was no bias regarding the effective use of codons, thereby ruling out translational selection.

### Linkage disequilibrium and recombination

The Z_nS_ estimator was used for evaluating LD between *pvron4* polymorphisms, giving 0.15. Linear regression between the LD and nucleotide distance showed a reduction in LD as distance increased, thereby suggesting recombination. However, the ZZ estimator gave −0.0019 and no minimum recombination sites were detected. Likewise, GARD methods found no breakpoints nor did RDP software reveal recombination tracks.

### The *Plasmodium vivax* Colombian population’s genetic structure regarding the *pvron4* locus

An AMOVA between the three Colombian regions was calculated for evaluating *pvron4* geospatial genetic diversity in Colombia, as well as Wright’s fixation index (F_ST_) between the different populations (regions). AMOVA revealed statistically significant differences between the sub-populations within the regions (F_SC_ = 0.06, p = 0.04). The Nariño sub-population (from the Pacific region) could be responsible for genetic differences regarding each of the other subpopulations (Additional files 3, 4). Calculating the F_ST_ index between populations (regions) gave values close to 0. There was a statistically significant difference between the Urabá/lower Cauca/southern Córdoba and Orinoquia-Amazonia regions (Table 4).

**Table 4:** Inter-population F_ST_ statistic for *pvron4*

F_ST_	Pacific coast	Urabá/lower Cauca/southern Córdoba	Orinoquia-Amazonia
Pacific coast		0.44043	0.31836
Urabá/lower Cauca/southern Córdoba	−0.00581		0.01465
Orinoquia-Amazonia	0.00542	*0.08128*	

F_ST_ was calculated for parasite populations in three Colombian regions. Values close to 0 indicate low genetic differentiation while values close to 1 indicate high genetic differentiation. Values below the diagonal indicate the F_ST_ value and those above the diagonal represent the p values. Values in italics indicate significant differences having p < 0.02

**Fig. 3:**
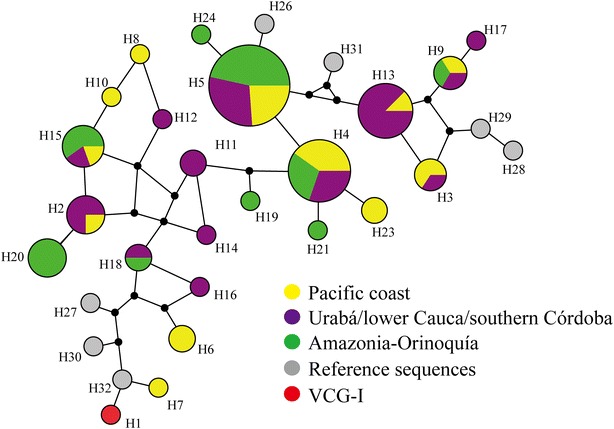
Median-joining network for Colombian regions. The *Figure* shows the *pvron4* haplotypes identified from the isolates from the three regions of Colombia. Haplotypes 22 and 28 were included within haplotype 15 using the contraction star algorithm [86] for simplifying interpretation of the network. Each node is a haplotype and its size indicates its frequency. The *lines* connecting the haplotypes represent the different mutational paths and the median vectors are the ancestral sequences explaining the relationship and evolutionary origin

A median joining network was used for better understanding the evolutionary relationship between *pvron4* haplotypes for describing the set of potential mutational pathways giving rise to the 32 haplotypes available for the locus (Fig. 3 and Additional file 3). The network showed that the parasite’s populations shared haplotypes, regardless of geographical region, which were related by different mutational pathways and ancestral sequences (median vectors) (Fig. 3 and Additional file 3). The most frequently occurring haplotypes were H5 (53.1 %), followed by H4 (31.2 %), H13 (25 %), H3 and H20 (12.5 % each). The presence of unique haplotypes in the Nariño (H7, H8 and H10) and Amazonas populations (H21, H22, H2) should be noted as they could be considered rare or specific regional alleles.

### Predicting *pvron4* putative domains and antigenic potential

Analysing the PvRON4 sequence from the Sal-I strain revealed the presence of a putative domain for the esterase/lipase (pfam05057) protein superfamily between amino acids 311–425 (nucleotides 931 to 1275, numbers based on the Sal-I reference sequence, GenBank access number: XM_001615228.1) e-value 1.10e-03. This domain was located after the repeat region and was highly conserved (Fig. 1).

According to antigenicity and B-cell linear epitope prediction, there was a potentially antigenic region between positions 41–171 and 178 up to 256 for haplotype 6 and up to position 340 for haplotype 17 (Additional file 5). This agreed with hydrophobicity and solvent accessibility predictions so that the PvRON4 N-terminal region seems to be a potential immune target. By contrast, the central region (following the repeat region) and the C-terminal seemed to be less antigenic, being less solvent-exposed (Additional file 5).

## Discussion

Various proteins contained within the parasite’s apical organelles seem to be crucial for host cell invasion and thus represent promising vaccine targets. RON complex proteins are among the proteins localized in the apical organelles, forming part of the TJ [17, 21, 63, 64]. This TJ plays a decisive role in parasite entry to a host cell and closure of the parasitophorous vacuole [17]. The RON4 protein located in the invasion complex is present in Phylum Apicomplexa members [15, 23, 24], suggesting that it forms part of a conserved invasion pathway. This protein has thus been described as a potential vaccine candidate.

A vaccine candidate must have several characteristics [10, 65]; one of them is to have low genetic diversity to avoid allele-specific immune responses, which could reduce vaccine efficacy. The analysis of *P. falciparum* laboratory strains from different geographically origins showed the *pfron4* locus as being a highly conserved locus, having just one amino acid substitution [23]. *Plasmodium vivax ron4* seems to have the same pattern, as analysing five reference sequences revealed low genetic diversity [25]. This study thus analysed 73 clinical isolates from the Colombian population for confirming *pvron4* as a highly conserved gene in *P. vivax*. In spite of increasing the number of sequences analysed, *pvron4* diversity remained low, the present study’s results showing that *pvron4* had lower genetic diversity than in a previous report [25]. Only eight SNPs were identified in the 80 available sequences compared to 14 previously identified ones [25]. Such high number of previously reported SNPs (i.e., 14) was due to erroneous repeat region alignment. *pvron4* had a similar pattern to that of other apical organelle proteins [66, 67]. *pvrap1* and *pvrap2* had 0.0009 to 0.001 π [67] while *pvron4* had a much lower value than *pvrap1*, suggesting it had low genetic diversity, the locus being more conserved to date for *P. vivax*. As it has been suggested for *pvrap1* and *pvra*p2 [67] the low diversity in *pvron4* could be the consequence of functional/structural constraint (see below) due to the key role of this protein in parasite invasion.

Even though *pvron4* was a highly conserved sequence regarding SNP occurrence, it had high polymorphism regarding size. Previous studies have identified two types of repeats towards the N-terminal of the encoded protein [22]. These repeats were reported as being imperfect copies of amino acids GGEH/SGEH/S and G/AEH. However, the analysis here performed showed that the *pvron4* repeat region consisted of two types of repeats having 100 % identity; the first encoded three GES amino acids (one to three copies) and the second one GEHGEHAEHGE amino acids (one to seven copies). These repeats gave a high number of different haplotypes (alleles) in *P. vivax*.

Previous studies have suggested that tandem repeats could play an important role as host immune response evasion mechanism [68–71]. In this study, 21 haplotypes were identified in PvRON4 when the InDels were analysed. PvRON4 N-terminal region seems to be the most exposed protein according to solvent availability and hydrophobicity results. This region (between signal peptide and repeat region) seems to be a potential antigenic target due to this being where the largest amount of potential B-cell linear epitopes was predicted. The repeat sequences identified broadened the solvent-exposed region and the protein’s antigenic potential. The PvRON4 N-terminal region could thus be the region exposed to a host’s immune system and repeats could be acting as an immunological smokescreen. Further antigenic and immunogenic studies are needed to confirm such hypothesis. As the repeat region was highly conserved regarding sequence, it could play an important structural or functional role, as has been suggested recently for the CSP [72, 73].

While the N-terminal region might to be exposed to the immune system, the central and C-terminal regions seem to be under functional constraint. Neutrality tests (e.g., Tajima, Fu and Li) gave no statistically significant values and neutrality was thus not ruled out. If *pvron4* is under neutrality then it should show high polymorphism unless there is a functional or structural constraint [74]. Given that this locus was highly conserved regarding sequence, functional/structural constraint is probable. However, selection at translational level could also be responsible for high conservation in the *pvron4* sequence. Analysing regarding preferential codon use did not reveal bias regarding codon use (ENC = 53–55). In fact this value was similar to that reported for the complete genome (ENC = 52.18) [75], suggesting that high *pvron4* locus conservation was not due to selection at translational level and could have been a result of strong purifying selection.

The d_S_ rate was significantly greater than the d_N_ rate according to Fisher’s exact test, suggesting that this locus has evolved under purifying selection. However, it is not easy to evaluate how natural selection acts in highly conserved antigens [66, 76, 77]. Previous studies have compared *P. vivax* sequences to phylogenetically related species to evaluate the effect of selection on parasite antigens [66, 76–78]. Sliding window analysis of ω rate gave values less than 1 towards the protein’s central region as well as towards the C-terminal. The K_S_ was statistically greater than K_N_ and various sites under purifying selection between species were detected in these regions, suggesting that purifying selection plays an important role during the locus’ evolution in the genus. Bearing in mind that functional regions tend to have slower evolution and are usually conserved between species [79], these results suggest that the PvRON4 C-terminal and central regions could be functionally important. The presence of conserved cysteines in the C-terminal portion (usually associated with protein–protein interaction) could be mediating the interaction between RON4 and AMA-1 and/or other RONs [16, 80], while the presence of a putative esterase/lipase domain in the protein’s central region could be involved in RON4 entry to the host cell.

*Plasmodium falciparum* and *T. gondii* studies have shown that the RON4 C-terminal region seems to play an important role in invasion [16, 24]. RON4 is located inside red blood cells (RBC), anchoring the AMA1/RON2 complex [17, 18, 24]; RON4 must thus be secreted and enter RBC during initial invasion stages by a yet-unknown mechanism. The presence of an esterase/lipase domain in the PvRON4 central region could provide a clue regarding the action mechanism. This is one of the protein’s most structured regions, being highly conserved among species and containing several sites under purifying selection, suggesting a functional/structural role. Therefore, while the PvRON4 N-terminal region seems to be associated with evasion of the immune response, the central region (containing the esterase/lipase domain) could be associated with the rupture of ester bonds in the phospholipids constituting host cell membrane. Such rupture would enable RON4 entry to RBC or hepatocyte cytoplasm. Once inside, the RON4 C-terminal region anchors RON proteins, which, in turn, enable AMA1-mediated interaction between the parasite and host cells. It can thus be hypothesized that such putative esterase/lipase domain could play a role regarding RON4 entry to a host cell, however, further functional assays are needed to confirm this.

In spite of *ron4* being highly conserved between species and that purifying selection seems to be important during this locus’ evolution in *Plasmodium*, some sites under positive selection were identified, coinciding with the regions where ω > 1 was observed. Previous studies have shown that some antigens (regardless of their genetic diversity) have regions/codons under episodic positive selection, which could have enabled adaptation to different hosts [76, 81, 82]. The topology obtained for *ron4* was similar to that obtained when analysing mitochondrial DNA [83]. The phylogenetic relationships of species infecting rodents and hominids can be seen in *ron4* phylogeny, however, such relationships have not been seen for species infecting monkeys. These species have a complex evolutionary history, which includes biogeographic aspects, adaptation to new macaque hosts and even a change from monkeys to humans [81, 84]. The episodic selection observed here might thus have been a consequence of this group of parasites’ rapid diversification (in the N-terminal region and a small portion of the C-terminal region) thereby enabling RON4 to adapt from an ancestral population to new available hosts, as previously suggested [76, 81, 82, 84].

A relatively high number of haplotypes has been found in the *pvron4* locus in Colombia, resulting from a combination of SNPs and tandem repeats. AMOVA analysis and median joining showed that Colombian regions shared most haplotypes and seemed to be genetically similar. However, it was observed that a 6 % of estimated variation between these regions was due to differences between the subpopulations constituting them. The F_ST_ value showed that some subpopulations might not be genetically similar; this could be associated with the presence of unique haplotypes. This agreed with studies in Colombia involving other parasite antigens [77], as well as mitochondrial DNA studies in America [85], suggesting that the parasite population in America is structured and has limited gene flow. However, since some subpopulations analysed here had limited sample size, the number of sequences must be increased for such results to be confirmed.

## Conclusions

Designing a vaccine which is completely effective against the parasites causing malaria requires antigens having limited genetic diversity to avoid allele-specific immune responses. The *pvron4* locus was seen to have low genetic diversity regarding SNPs but had a large amount of haplotypes due to tandem repeats located in the proteins’ N-terminal, which could be involved in evading the immune response. On the other hand, the central and C-terminal regions are highly conserved, even between species. Such regions are under purifying selection, suggesting that they are under functionally or structurally constraint. The central region has a putative esterase/lipase domain, leading to the hypothesis that this domain enables RON4 entry to host cells while the C-terminal region anchors the AMA1/RON complex. Bearing the aforementioned results in mind, PvRON4 central/C-terminal region would seem to be a promising candidate for inclusion when designing a subunit-based vaccine against *P. vivax*.

## Additional files

**Additional file 1.** The sequences of the 73 Colombian isolates obtained in this study, 7 reference sequences and 13 orthologous sequences from the *ron4* locus.

**Additional file 2.** Aligning the 32 haplotypes identified for *pvron4.*

**Additional file 3.** Median–joining network for Colombian departments. The Figure shows the evaluative relationship between the 32 *pvron4* haplotypes identified from isolates from five Colombian departments, together with the reference sequence. Haplotypes 22 and 28 were included within haplotype 15 when using the star contraction algorithm [86] for simplifying interpretation of the network. Each node is a haplotype and its size indicates its frequency. The lines connecting the haplotypes represent mutational paths and the median vectors are the ancestral sequences explaining the evolutionary relationship and origin.

**Additional file 4.** Inter-population F_ST_ statistic for *pvron4* per department. F_ST_ was calculated for parasite subpopulations in Colombia. Values close to 0 indicate low genetic differentiation while values close to 1 indicate high genetic differentiation. Values below the diagonal indicate the F_ST_ value and those above the diagonal represent the p-values. Values in bold indicate significant differences having p <0.03.

**Additional file 5.** Predicting potentially antigenic regions in PvRON4. Predictive tests for A. Linear B epitopes (threshold=0.35); B. Kolaskar and Tongaonkar antigenicity (threshold=1.00); C. Parker hydrophobicity (threshold=2.31) and D. Accessibility to solvent (threshold=1,000) using haplotype 6 and 17 sequences. The dotted line shows the regions having the greatest probability of being recognised by the immune system according to score on each prediction. The inverted antigenicity values arose from calculating antigenic propensity, regardless of the possible occurrence of amino acids in epitopes and on protein surface.

## Abbreviations

AMA1: apical membrane antigen 1
AMOVA: analysis of molecular variance
BepiPred: B cell epitope prediction
CBI: codon bias index
CSP: circumsporozoite protein
d_**N**_: non-synonymous substitutions per non-synonym site
d_**S**_: synonyms substitutions per synonym site
ENC: effective number of codons
FEL: fixed effects likelihood
F_**SC**_: F-statistic, used to estimate the proportion of genetic variability found among populations within groups
F_**ST**_: Wright’s fixation index, used to estimate the proportion of genetic variability found among populations
FUBAR: fast, unconstrained Bayesian approximation for inferring selection
GARD: genetic algorithm recombination detection
IEDB: immune epitope database and analysis resource
IFEL: internal fixed effects likelihood
InDels: insertions/deletions
K_**N**_: average number of non-synonyms divergences per non-synonym site
K_**S**_: average number of synonyms divergences per synonym site
LD: linkage disequilibrium
MEME: mixed effects model of evolution
MK: McDonald–Kretiman test
N’c: ENC prime
PCR–RFLP: polymerase chain reaction-restriction fragment length polymorphism
*pfron4*: gene encoding *Plasmodium falciparum* rhoptry neck protein 4
*pvmsp*-*3α*: alpha subunit of gene encoding *Plasmodium vivax* merozoite surface protein-3
*pvrap1*: gene encoding *Plasmodium vivax* rhoptry-associated protein 1
*pvrap2*: gene encoding *Plasmodium vivax* rhoptry-associated protein 2
*pvron4*: gene encoding *Plasmodium vivax* rhoptry neck protein 4
RDP3: recombination detection program 3
REL: random effects likelihood
Rm: minimum number of recombination events
RON2: *Plasmodium vivax* rhoptry neck protein 2
RONs: Rhoptry neck proteins
SLAC: single likelihood ancestor counting
SNP: single nucleotide polymorphisms
*Tg*RON4: *Toxoplasma gondii* rhoptry neck protein 4
TJ: tight junction
VCG-I: Vivax Colombia Guainía-I
