# Supplementary material for: Size polymorphism and low sequence diversity in the locus encoding the Plasmodium vivax rhoptry neck protein 4 (PvRON4) in Colombian isolates
Source: Malar J. 2016 Oct 18;15:501. doi: 10.1186/s12936-016-1563-4 (PMC5069803; doi:10.1186/s12936-016-1563-4)
Supplement: Supplementary file 2 — Additional file 2. Aligning the 32 haplotypes identified for pvron4. [file 12936_2016_1563_MOESM2_ESM.pdf]

Size polymorphism and low sequence diversity in the locus encoding the *Plasmodium vivax* rhoptry neck protein 4 (PvRON4) in Colombian isolates

Additional file 2. Aligning the 32 haplotypes identified for *pvron4*.

```
                                   *        20         *        40         *        60         *        80         *       100
H1_VCG                 : ATGTCTCGTAAAAGGGTTTTTCTTCTGTGCATTTTTCTGGCCATTTCCGCCGTTGTTGACGAAGCCGAGTCGTTCAGGAGTAAGCAAAAAGAAAGGAAAAAAAA :  104
H2                     : ATGTCTCGTAAAAGGGTTTTTCTTCTGTGCATTTTTCTGGCCATTTCCGCCGTTGTTGACGAAGCCGAGTCGTTCAGGAGTAAGCAAAAAGAAAGGAAAAAAAA :  104
H3                     : ATGTCTCGTAAAAGGGTTTTTCTTCTGTGCATTTTTCTGGCCATTTCCGCCGTTGTTGACGAAGCCGAGTCGTTCAGGAGTAAGCAAAAAGAAAGGAAAAAAAA :  104
H4                     : ATGTCTCGTAAAAGGGTTTTTCTTCTGTGCATTTTTCTGGCCATTTCCGCCGTTGTTGACGAAGCCGAGTCGTTCAGGAGTAAGCAAAAAGAAAGGAAAAAAAA :  104
H5                     : ATGTCTCGTAAAAGGGTTTTTCTTCTGTGCATTTTTCTGGCCATTTCCGCCGTTGTTGACGAAGCCGAGTCGTTCAGGAGTAAGCAAAAAGAAAGGAAAAAAAA :  104
H6                     : ATGTCTCGTAAAAGGGTTTTTCTTCTGTGCATTTTTCTGGCCATTTCCGCCGTTGTTGACGAAGCCGAGTCGTTCAGGAGTAAGCAAAAAGAAAGGAAAAAAAA :  104
H7                     : ATGTCTCGTAAAAGGGTTTTTCTTCTGTGCATTTTTCTGGCCATTTCCGCCGTTGTTGACGAAGCCGAGTCGTTCAGGAGTAAGCAAAAAGAAAGGAAAAAAAA :  104
H8                     : ATGTCTCGTAAAAGGGTTTTTCTTCTGTGCATTTTTCTGGCCATTTCCGCCGTTGTTGACGAAGCCGAGTCGTTCAGGAGTAAGCAAAAAGAAAGGAAAAAAAA :  104
H9                     : ATGTCTCGTAAAAGGGTTTTTCTTCTGTGCATTTTTCTGGCCATTTCCGCCGTTGTTGACGAAGCCGAGTCGTTCAGGAGTAAGCAAAAAGAAAGGAAAAAAAA :  104
H10                    : ATGTCTCGTAAAAGGGTTTTTCTTCTGTGCATTTTTCTGGCCATTTCCGCCGTTGTTGACGAAGCCGAGTCGTTCAGGAGTAAGCAAAAAGAAAGGAAAAAAAA :  104
H11                    : ATGTCTCGTAAAAGGGTTTTTCTTCTGTGCATTTTTCTGGCCATTTCCGCCGTTGTTGACGAAGCCGAGTCGTTCAGGAGTAAGCAAAAAGAAAGGAAAAAAAA :  104
H12                    : ATGTCTCGTAAAAGGGTTTTTCTTCTGTGCATTTTTCTGGCCATTTCCGCCGTTGTTGACGAAGCCGAGTCGTTCAGGAGTAAGCAAAAAGAAAGGAAAAAAAA :  104
H13                    : ATGTCTCGTAAAAGGGTTTTTCTTCTGTGCATTTTTCTGGCCATTTCCGCCGTTGTTGACGAAGCCGAGTCGTTCAGGAGTAAGCAAAAAGAAAGGAAAAAAAA :  104
H14                    : ATGTCTCGTAAAAGGGTTTTTCTTCTGTGCATTTTTCTGGCCATTTCCGCCGTTGTTGACGAAGCCGAGTCGTTCAGGAGTAAGCAAAAAGAAAGGAAAAAAAA :  104
H15                    : ATGTCTCGTAAAAGGGTTTTTCTTCTGTGCATTTTTCTGGCCATTTCCGCCGTTGTTGACGAAGCCGAGTCGTTCAGGAGTAAGCAAAAAGAAAGGAAAAAAAA :  104
H16                    : ATGTCTCGTAAAAGGGTTTTTCTTCTGTGCATTTTTCTGGCCATTTCCGCCGTTGTTGACGAAGCCGAGTCGTTCAGGAGTAAGCAAAAAGAAAGGAAAAAAAA :  104
H17                    : ATGTCTCGTAAAAGGGTTTTTCTTCTGTGCATTTTTCTGGCCATTTCCGCCGTTGTTGACGAAGCCGAGTCGTTCAGGAGTAAGCAAAAAGAAAGGAAAAAAAA :  104
H18                    : ATGTCTCGTAAAAGGGTTTTTCTTCTGTGCATTTTTCTGGCCATTTCCGCCGTTGTTGACGAAGCCGAGTCGTTCAGGAGTAAGCAAAAAGAAAGGAAAAAAAA :  104
H19                    : ATGTCTCGTAAAAGGGTTTTTCTTCTGTGCATTTTTCTGGCCATTTCCGCCGTTGTTGACGAAGCCGAGTCGTTCAGGAGTAAGCAAAAAGAAAGGAAAAAAAA :  104
H20                    : ATGTCTCGTAAAAGGGTTTTTCTTCTGTGCATTTTTCTGGCCATTTCCGCCGTTGTTGACGAAGCCGAGTCGTTCAGGAGTAAGCAAAAAGAAAGGAAAAAAAA :  104
H21                    : ATGTCTCGTAAAAGGGTTTTTCTTCTGTGCATTTTTCTGGCCATTTCCGCCGTTGTTGACGAAGCCGAGTCGTTCAGGAGTAAGCAAAAAGAAAGGAAAAAAAA :  104
H22                    : ATGTCTCGTAAAAGGGTTTTTCTTCTGTGCATTTTTCTGGCCATTTCCGCCGTTGTTGACGAAGCCGAGTCGTTCAGGAGTAAGCAAAAAGAAAGGAAAAAAAA :  104
H23                    : ATGTCTCGTAAAAGGGTTTTTCTTCTGTGCATTTTTCTGGCCATTTCCGCCGTTGTTGACGAAGCCGAGTCGTTCAGGAGTAAGCAAAAAGAAAGGAAAAAAAA :  104
H24                    : ATGTCTCGTAAAAGGGTTTTTCTTCTGTGCATTTTTCTGGCCATTTCCGCCGTTGTTGACGAAGCCGAGTCGTTCAGGAGTAAGCAAAAAGAAAGGAAAAAAAA :  104
H25                    : ATGTCTCGTAAAAGGGTTTTTCTTCTGTGCATTTTTCTGGCCATTTCCGCCGTTGTTGACGAAGCCGAGTCGTTCAGGAGTAAGCAAAAAGAAAGGAAAAAAAA :  104
H26_Mauritania-I_ref   : ATGTCTCGTAAAAGGGTTTTTCTTCTGTGCATTTTTCTGGCCATTTCCGCCGTTGTTGACGAAGCCGAGTCGTTCAGGAGTAAGCAAAAAGAAAGGAAAAAAAA :  104
H27_India-VII_refseq   : ATGTCTCGTAAAAGGGTTTTTCTTCTGTGCATTTTTCTGGCCATTTCCGCCGTTGTTGACGAAGCCGAGTCGTTCAGGAGTAAGCAAAAAGAAAGGAAAAAAAA :  104
H28_Ctg_refsequence    : ATGTCTCGTAAAAGGGTTTTTCTTCTGTGCATTTTTCTGGCCATTTCCGCCGTTGTTGACGAAGCCGAGTCGTTCAGGAGTAAGCAAAAAGAAAGGAAAAAAAA :  104
H29_Salvador-I_refse   : ATGTCTCGTAAAAGGGTTTTTCTTCTGTGCATTTTTCTGGCCATTTCCGCCGTTGTTGACGAAGCCGAGTCGTTCAGGAGTAAGCAAAAAGAAAGGAAAAAAAA :  104
H30_NKorea_refsequen   : ATGTCTCGTAAAAGGGTTTTTCTTCTGTGCATTTTTCTGGCCATTTCCGCCGTTGTTGACGAAGCCGAGTCGTTCAGGAGTAAGCAAAAAGAAAGGAAAAAAAA :  104
H31_P.vivax01_refseq   : ATGTCTCGTAAAAGGGTTTTTCTTCTGTGCATTTTTCTGGCCATTTCCGCCGTTGTTGACGAAGCCGAGTCGTTCAGGAGTAAGCAAAAAGAAAGGAAAAAAAA :  104
H32_Brazil-I_refsequ   : ATGTCTCGTAAAAGGGTTTTTCTTCTGTGCATTTTTCTGGCCATTTCCGCCGTTGTTGACGAAGCCGAGTCGTTCAGGAGTAAGCAAAAAGAAAGGAAAAAAAA :  104
                         ATGTCTCGTAAAAGGGTTTTTCTTCTGTGCATTTTTCTGGCCATTTCCGCCGTTGTTGACGAAGCCGAGTCGTTCAGGAGTAAGCAAAAAGAAAGGAAAAAAAA

                               *       120         *       140         *       160         *       180         *       200
H1_VCG                 : GGAGAGATAGGTAGAGAGGGGCATCACACGCAACTGCTATACGCATGCAACTGCGATACGCACGTGACTGCGGTACACACCTGTGCAATGGCGCGTGCCGTATG :  208
H2                     : GGAGAGATAGGTAGAGAGGGGCATCACACGCAACTGCTATACGCATGCAACTGCGATACGCACGTGACTGCGGTACACACCTGTGCAATGGCGCGTGCCGTATG :  208
H3                     : GGAGAGATAGGTAGAGAGGGGCATCACACGCAACTGCTATACGCATGCAACTGCGATACGCACGTGACTGCGGTACACACCTGTGCAATGGCGCGTGCCGTATG :  208
H4                     : GGAGAGATAGGTAGAGAGGGGCATCACACGCAACTGCTATACGCATGCAACTGCGATACGCACGTGACTGCGGTACACACCTGTGCAATGGCGCGTGCCGTATG :  208
H5                     : GGAGAGATAGGTAGAGAGGGGCATCACACGCAACTGCTATACGCATGCAACTGCGATACGCACGTGACTGCGGTACACACCTGTGCAATGGCGCGTGCCGTATG :  208
H6                     : GGAGAGATAGGTAGAGAGGGGCATCACACGCAACTGCTATACGCATGCAACTGCGATACGCACGTGACTGCGGTACACACCTGTGCAATGGCGCGTGCCGTATG :  208
H7                     : GGAGAGATAGGTAGAGAGGGGCATCACACGCAACTGCTATACGCATGCAACTGCGATACGCACGTGACTGCGGTACACACCTGTGCAATGGCGCGTGCCGTATG :  208
```

```
H8                  : GGAGAGATAGGTAGAGAGGGGCATCACACGCAACTGCTATACGCATGCAACTGCGATACGCACGTGACTGCGGTACACACCTGTGCAATGGCGCGTGCCGTATG :  208
H9                  : GGAGAGATAGGTAGAGAGGGGCATCACACGCAACTGCTATACGCATGCAACTGCG-----------------GTACACACCTGTGCAATGGCGCGTGCCGTATG :  191
H10                 : GGAGAGATAGGTAGAGAGGGGCATCACACGCAACTGCTATACGCATGCAACTGCGATACGCACGTGACTGCGGTACACACCTGTGCAATGGCGCGTGCCGTATG :  208
H11                 : GGAGAGATAGGTAGAGAGGGGCATCACACGCAACTGCTATACGCATGCAACTGCGATACGCACGTGACTGCGGTACACACCTGTGCAATGGCGCGTGCCGTATG :  208
H12                 : GGAGAGATAGGTAGAGAGGGGCATCACACGCAACTGCTATACGCATGCAACTGCGATACGCACGTGACTGCGGTACACACCTGTGCAATGGCGCGTGCCGTATG :  208
H13                 : GGAGAGATAGGTAGAGAGGGGCATCACACGCAACTGCTATACGCATGCAACTGCGATACGCACGTGACTGCGGTACACACCTGTGCAATGGCGCGTGCCGTATG :  208
H14                 : GGAGAGATAGGTAGAGAGGGGCATCACACGCAACTGCTATACGCATGCAACTGCGATACGCACGTGACTGCGGTACACACCTGTGCAATGGCGCGTGCCGTATG :  208
H15                 : GGAGAGATAGGTAGAGAGGGGCATCACACGCAACTGCTATACGCATGCAACTGCGATACGCACGTGACTGCGGTACACACCTGTGCAATGGCGCGTGCCGTATG :  208
H16                 : GGAGAGATAGGTAGAGAGGGGCATCACACGCAACTGCTATACGCATGCAACTGCGATACGCACGTGACTGCGGTACACACCTGTGCAATGGCGCGTGCCGTATG :  208
H17                 : GGAGAGATAGGTAGAGAGGGGCATCACACGCAACTGCTATACGCATGCAACTGCGATACGCACGTGACTGCGGTACACACCTGTGCAATGGCGCGTGCCGTATG :  208
H18                 : GGAGAGATAGGTAGAGAGGGGCATCACACGCAACTGCTATACGCATGCAACTGCGATACGCACGTGACTGCGGTACACACCTGTGCAATGGCGCGTGCCGTATG :  208
H19                 : GGAGAGATAGGTAGAGAGGGGCATCACACGCAACTGCTATACGCATGCAACTGCGATACGCACGTGACTGCGGTACACACCTGTGCAATGGCGCGTGCCGTATG :  208
H20                 : GGAGAGATAGGTAGAGAGGGGCATCACACGCAACTGCTATACGCATGCAACTGCGATACGCACGTGACTGCGGTACACACCTGTGCAATGGCGCGTGCCGTATG :  208
H21                 : GGAGAGATAGGTAGAGAGGGGCATCACACGCAACTGCTATACGCATGCAACTGCGATACGCACGTGACTGCGGTACACACCTGTGCAATGGCGCGTGCCGTATG :  208
H22                 : GGAGAGATAGGTAGAGAGGGGCATCACACGCAACTGCTATACGCATGCAACTGCGATACGCACGTGACTGCGGTACACACCTGTGCAATGGCGCGTGCCGTATG :  208
H23                 : GGAGAGATAGGTAGAGAGGGGCATCACACGCAACTGCTATACGCATGCAACTGCGATACGCACGTGACTGCGGTACACACCTGTGCAATGGCGCGTGCCGTATG :  208
H24                 : GGAGAGATAGGTAGAGAGGGGCATCACACGCAACTGCTATACGCATGCAACTGCGATACGCACGTGACTGCGGTACACACCTGTGCAATGGCGCGTGCCGTATG :  208
H25                 : GGAGAGATAGGTAGAGAGGGGCATCACACGCAACTGCTATACGCATGCAACTGCGATACGCACGTGACTGCGGTACACACCTGTGCAATGGCGCGTGCCGTATG :  208
H26_Mauritania-I_ref : GGAGAGATAGGTAGAGAGGGGCATCACACGCAACTGCTATACGCATGCAACTGCGATACGCACGTGACTGCGGTACACACCTGTGCAATGGCGCGTGCCGTATG :  208
H27_India-VII_refseq : GGAGAGATAGGTAGAGAGGGGCATCACACGCAACTGCTATACGCATGCAACTGCGATACGCACGTGACTGCGGTACACACCTGTGCAATGGCGCGTGCCGTATG :  208
H28_Ctg_refsequence  : GGAGAGATAGGTAGAGAGGGGCATCACACGCAACTGCTATACGCATGCAACTGCGATACGCACGTGACTGCGGTACACACCTGTGCAATGGCGCGTGCCGTATG :  208
H29_Salvador-I_refse : GGAGAGATAGGTAGAGAGGGGCATCACACGCAACTGCTATACGCATGCAACTGCGATACGCACGTGACTGCGGTACACACCTGTGCAATGGCGCGTGCCGTATG :  208
H30_NKorea_refsequen : GGAGAGATAGGTAGAGAGGGGCATCACACGCAACTGCTATACGCATGCAACTGCGATACGCACGTGACTGCGGTACACACCTGTGCAATGGCGCGTGCCGTATG :  208
H31_P.vivax01_refseq : GGAGAGATAGGTAGAGAGGGGCATCACACGCAACTGCTATACGCATGCAACTGCGATACGCACGTGACTGCGGTACACACCTGTGCAATGGCGCGTGCCGTATG :  208
H32_Brazil-I_refsequ : GGAGAGATAGGTAGAGAGGGGCATCACACGCAACTGCTATACGCATGCAACTGCGATACGCACGTGACTGCGGTACACACCTGTGCAATGGCGCGTGCCGTATG :  208
                       GGAGAGATAGGTAGAGAGGGGCATCACACGCAACTGCTATACGCATGCAACTGCGatacgcacgtgactgcgGTACACACCTGTGCAATGGCGCGTGCCGTATG

                         *       220         *       240         *       260         *       280         *       300         *
H1_VCG              : CATGCTGTCCACGGCGCTCCACCCCCAAAGCAACGAAATAAAAAATAAAAAGTAAATAAATAAATAAATAAATTCCCCCTTTCAGAGCCGGACGCGGCAGCGAA :  312
H2                  : CATGCTGTCCACGGCGCTCCACCCCCAAAGCAACGAAATAAAAAATAAAAAGTAAATAAATAAATAAATAAATTCCCCCTTTCAGAGCCGGACGCGGCAGCGAA :  312
H3                  : CATGCTGTCCACGGCGCTCCACCCCCAAAGCAACGAAATAAAAAATAAAAAGTAAATAAATAAATAAATAAATTCCCCCTTTCAGAGCCAGACGCGGCAGCGAA :  312
H4                  : CATGCTGTCCACGGCGCTCCACCCCCAAAGCAACGAAATAAAAAATAAAAAGTAAATAAATAAATAAATAAATTCCCCCTTTCAGAGCCAGACGCGGCAGCGAA :  312
H5                  : CATGCTGTCCACGGCGCTCCACCCCCAAAGCAACGAAATAAAAAATAAAAAGTAAATAAATAAATAAATAAATTCCCCCTTTCAGAGCCGGACGCGGCAGCGAA :  312
H6                  : CATGCTGTCCACGGCGCTCCACCCCCAAAGCAACGAAATAAAAAATAAAAAGTAAATAAATAAATAAATAAATTCCCCCTTTCAGAGCCGGACGCGGCAGCGAA :  312
H7                  : CATGCTGTCCACGGCGCTCCACCCCCAAAGCAACGAAATAAAAAATAAAAAGTAAATAAATAAATAAATAAATTCCCCCTTTCAGAGCCAGACGCGGCAGCGAA :  312
H8                  : CATGCTGTCCACGGCGCTCCACCCCCAAAGCAACGAAATAAAAAATAAAAAGTAAATAAATAAATAAATAAATTCCCCCTTTCAGAGCCAGACGCGGCAGCGAA :  312
H9                  : CATGCTGTCCACGGCGCTCCACCCCCAAAGCAACGAAATAAAAAATAAAAAGTAAATAAATAAATAAATAAATTCCCCCTTTCAGAGCCGGACGCGGCAGCGAA :  295
H10                 : CATGCTGTCCACGGCGCTCCACCCCCAAAGCAACGAAATAAAAAATAAAAAGTAAATAAATAAATAAATAAATTCCCCCTTTCAGAGCCAGACGCGGCAGCGAA :  312
H11                 : CATGCTGTCCACGGCGCTCCACCCCCAAAGCAACGAAATAAAAAATAAAAAGTAAATAAATAAATAAATAAATTCCCCCTTTCAGAGCCAGACGCGGCAGCGAA :  312
H12                 : CATGCTGTCCACGGCGCTCCACCCCCAAAGCAACGAAATAAAAAATAAAAAGTAAATAAATAAATAAATAAATTCCCCCTTTCAGAGCCAGACGCGGCAGCGAA :  312
H13                 : CATGCTGTCCACGGCGCTCCACCCCCAAAGCAACGAAATAAAAAATAAAAAGTAAATAAATAAATAAATAAATTCCCCCTTTCAGAGCCGGACGCGGCAGCGAA :  312
H14                 : CATGCTGTCCACGGCGCTCCACCCCCAAAGCAACGAAATAAAAAATAAAAAGTAAATAAATAAATAAATAAATTCCCCCTTTCAGAGCCGGACGCGGCAGCGAA :  312
H15                 : CATGCTGTCCACGGCGCTCCACCCCCAAAGCAACGAAATAAAAAATAAAAAGTAAATAAATAAATAAATAAATTCCCCCTTTCAGAGCCAGACGCGGCAGCGAA :  312
H16                 : CATGCTGTCCACGGCGCTCCACCCCCAAAGCAACGAAATAAAAAATAAAAAGTAAATAAATAAATAAATAAATTCCCCCTTTCAGAGCCGGACGCGGCAGCGAA :  312
H17                 : CATGCTGTCCACGGCGCTCCACCCCCAAAGCAACGAAATAAAAAATAAAAAGTAAATAAATAAATAAATAAATTCCCCCTTTCAGAGCCGGACGCGGCAGCGAA :  312
H18                 : CATGCTGTCCACGGCGCTCCACCCCCAAAGCAACGAAATAAAAAATAAAAAGTAAATAAATAAATAAATAAATTCCCCCTTTCAGAGCCGGACGCGGCAGCGAA :  312
H19                 : CATGCTGTCCACGGCGCTCCACCCCCAAAGCAACGAAATAAAAAATAAAAAGTAAATAAATAAATAAATAAATTCCCCCTTTCAGAGCCAGACGCGGCAGCGAA :  312
H20                 : CATGCTGTCCACGGCGCTCCACCCCCAAAGCAACGAAATAAAAAATAAAAAGTAAATAAATAAATAAATAAATTCCCCCTTTCAGAGCCGGACGCGGCAGCGAA :  312
H21                 : CATGCTGTCCACGGCGCTCCACCCCCAAAGCAACGAAATAAAAAATAAAAAGTAAATAAATAAATAAATAAATTCCCCCTTTCAGAGCCAGACGCGGCAGCGAA :  312
H22                 : CATGCTGTCCACGGCGCTCCACCCCCAAAGCAACGAAATAAAAAATAAAAAGTAAATAAATAAATAAATAAATTCCCCCTTTCAGAGCCAGACGCGGCAGCGAA :  312
```

```
H23                   : CATGCTGTCCACGGCGCTCCACCCCCAAAGCAACGAAATAAAAAATAAAAAGTAAATAAATAAATAAATAAATTCCCCCTTTCAGAGCCAGACGCGGCAGCGAA : 312
H24                   : CATGCTGTCCACGGCGCTCCACCCCCAAAGCAACGAAATAAAAAATAAAAAGTAAATAAATAAATAAATAAATTCCCCCTTTCAGAGCCGGACGCGGCAGCGAA : 312
H25                   : CATGCTGTCCACGGCGCTCCACCCCCAAAGCAACGAAATAAAAAATAAAAAGTAAATAAATAAATAAATAAATTCCCCCTTTCAGAGCCAGACGCGGCAGCGAA : 312
H26_Mauritania-I_ref  : CATGCTGTCCACGGCGCTCCACCCCCAAAGCAACGAAATAAAAAATAAAAAGTAAATAAATAAATAAATAAATTCCCCCTTTCAGAGCCGGACGCGGCAGCGAA : 312
H27_India-VII_refseq  : CATGCTGTCCACGGCGCTCCACCCCCAAAGCAACGAAATAAAAAATAAAAAGTAAATAAATAAATAAATAAATTCCCCCTTTCAGAGCCGGACGCGGCAGCGAA : 312
H28_Ctg_refsequence   : CATGCTGTCCACGGCGCTCCACCCCCAAAGCAACGAAATAAAAAATAAAAAGTAAATAAATAAATAAATAAATTCCCCCTTTCAGAGCCAGACGCGGCAGCGAA : 312
H29_Salvador-I_refse  : CATGCTGTCCACGGCGCTCCACCCCCAAAGCAACGAAATAAAAAATAAAAAGTAAATAAATAAATAAATAAATTCCCCCTTTCAGAGCCAGACGCGGCAGCGAA : 312
H30_NKorea_refsequen  : CATGCTGTCCACGGCGCTCCACCCCCAAAGCAACGAAATAAAAAATAAAAAGTAAATAAATAAATAAATAAATTCCCCCTTTCAGAGCCGGACGCGGCAGCGAA : 312
H31_P.vivax01_refseq  : CATGCTGTCCACGGCGCTCCACCCCCAAAGCAACGAAATAAAAAATAAAAAGTAAATAAATAAATAAATAAATTCCCCCTTTCAGAGCCGGACGCGGCAGCGAA : 312
H32_Brazil-I_refsequ  : CATGCTGTCCACGGCGCTCCACCCCCAAAGCAACGAAATAAAAAATAAAAAGTAAATAAATAAATAAATAAATTCCCCCTTTCAGAGCCGGACGCGGCAGCGAA : 312
                        CATGCTGTCCACGGCGCTCCACCCCCAAAGCAACGAAATAAAAAATAAAAAGTAAATAAATAAATAAATAAATTCCCCCTTTCAGAGCC GACGCGGCAGCGAA

                             320         *       340         *       360         *       380         *       400         *
H1_VCG                : CGTCTCCTTCCTGGAGTTCACAGCAGATGGCAACGCTGAAGAGGGGCAGGCGCAGCCAAGTGAACCCATCGTTAACACCCAGCCGCTGCAGGAAAATGTAAGTG : 416
H2                    : CGTCTCCTTCCTGGAGTTCACAGCAGATGGCAACGCTGAAGAGGGGCAGGCGCAGCCAAGTGAACCCATCGTTAACACCCAGCCGCTGCAGGAAAATGTAAGTG : 416
H3                    : CGTCTCCTTCCTGGAGTTCACAGCAGATGGCAACGCTGAAGAGGGGCAGGCGCAGCCAAGTGAACCCATCGTTAACACCCAGCCGCTGCAGGAAAATGTAAGTG : 416
H4                    : CGTCTCCTTCCTGGAGTTCACAGCAGATGGCAACGCTGAAGAGGGGCAGGCGCAGCCAAGTGAACCCATCGTTAACACCCAGCCGCTGCAGGAAAATGTAAGTG : 416
H5                    : CGTCTCCTTCCTGGAGTTCACAGCAGATGGCAACGCTGAAGAGGGGCAGGCGCAGCCAAGTGAACCCATCGTTAACACCCAGCCGCTGCAGGAAAATGTAAGTG : 416
H6                    : CGTCTCCTTCCTGGAGTTCACAGCAGATGGCAACGCTGAAGAGGGGCAGGCGCAGCCAAGTGAACCCATCGTTAACACCCAGCCGCTGCAGGAAAATGTAAGTG : 416
H7                    : CGTCTCCTTCCTGGAGTTCACAGCAGATGGCAACGCTGAAGAGGGGCAGGCGCAGCCAAGTGAACCCATCGTTAACACCCAGCCGCTGCAGGAAAATGTAAGTG : 416
H8                    : CGTCTCCTTCCTGGAGTTCACAGCAGATGGCAACGCTGAAGAGGGGCAGGCGCAGCCAAGTGAACCCATCGTTAACACCCAGCCGCTGCAGGAAAATGTAAGTG : 416
H9                    : CGTCTCCTTCCTGGAGTTCACAGCAGATGGCAACGCTGAAGAGGGGCAGGCGCAGCCAAGTGAACCCATCGTTAACACCCAGCCGCTGCAGGAAAATGTAAGTG : 399
H10                   : CGTCTCCTTCCTGGAGTTCACAGCAGATGGCAACGCTGAAGAGGGGCAGGCGCAGCCAAGTGAACCCATCGTTAACACCCAGCCGCTGCAGGAAAATGTAAGTG : 416
H11                   : CGTCTCCTTCCTGGAGTTCACAGCAGATGGCAACGCTGAAGAGGGGCAGGCGCAGCCAAGTGAACCCATCGTTAACACCCAGCCGCTGCAGGAAAATGTAAGTG : 416
H12                   : CGTCTCCTTCCTGGAGTTCACAGCAGATGGCAACGCTGAAGAGGGGCAGGCGCAGCCAAGTGAACCCATCGTTAACACCCAGCCGCTGCAGGAAAATGTAAGTG : 416
H13                   : CGTCTCCTTCCTGGAGTTCACAGCAGATGGCAACGCTGAAGAGGGGCAGGCGCAGCCAAGTGAACCCATCGTTAACACCCAGCCGCTGCAGGAAAATGTAAGTG : 416
H14                   : CGTCTCCTTCCTGGAGTTCACAGCAGATGGCAACGCTGAAGAGGGGCAGGCGCAGCCAAGTGAACCCATCGTTAACACCCAGCCGCTGCAGGAAAATGTAAGTG : 416
H15                   : CGTCTCCTTCCTGGAGTTCACAGCAGATGGCAACGCTGAAGAGGGGCAGGCGCAGCCAAGTGAACCCATCGTTAACACCCAGCCGCTGCAGGAAAATGTAAGTG : 416
H16                   : CGTCTCCTTCCTGGAGTTCACAGCAGATGGCAACGCTGAAGAGGGGCAGGCGCAGCCAAGTGAACCCATCGTTAACACCCAGCCGCTGCAGGAAAATGTAAGTG : 416
H17                   : CGTCTCCTTCCTGGAGTTCACAGCAGATGGCAACGCTGAAGAGGGGCAGGCGCAGCCAAGTGAACCCATCGTTAACACCCAGCCGCTGCAGGAAAATGTAAGTG : 416
H18                   : CGTCTCCTTCCTGGAGTTCACAGCAGATGGCAACGCTGAAGAGGGGCAGGCGCAGCCAAGTGAACCCATCGTTAACACCCAGCCGCTGCAGGAAAATGTAAGTG : 416
H19                   : CGTCTCCTTCCTGGAGTTCACAGCAGATGGCAACGCTGAAGAGGGGCAGGCGCAGCCAAGTGAACCCATCGTTAACACCCAGCCGCTGCAGGAAAATGTAAGTG : 416
H20                   : CGTCTCCTTCCTGGAGTTCACAGCAGATGGCAACGCTGAAGAGGGGCAGGCGCAGCCAAGTGAACCCATCGTTAACACCCAGCCGCTGCAGGAAAATGTAAGTG : 416
H21                   : CGTCTCCTTCCTGGAGTTCACAGCAGATGGCAACGCTGAAGAGGGGCAGGCGCAGCCAAGTGAACCCATCGTTAACACCCAGCCGCTGCAGGAAAATGTAAGTG : 416
H22                   : CGTCTCCTTCCTGGAGTTCACAGCAGATGGCAACGCTGAAGAGGGGCAGGCGCAGCCAAGTGAACCCATCGTTAACACCCAGCCGCTGCAGGAAAATGTAAGTG : 416
H23                   : CGTCTCCTTCCTGGAGTTCACAGCAGATGGCAACGCTGAAGAGGGGCAGGCGCAGCCAAGTGAACCCATCGTTAACACCCAGCCGCTGCAGGAAAATGTAAGTG : 416
H24                   : CGTCTCCTTCCTGGAGTTCACAGCAGATGGCAACGCTGAAGAGGGGCAGGCGCAGCCAAGTGAACCCATCGTTAACACCCAGCCGCTGCAGGAAAATGTAAGTG : 416
H25                   : CGTCTCCTTCCTGGAGTTCACAGCAGATGGCAACGCTGAAGAGGGGCAGGCGCAGCCAAGTGAACCCATCGTTAACACCCAGCCGCTGCAGGAAAATGTAAGTG : 416
H26_Mauritania-I_ref  : CGTCTCCTTCCTGGAGTTCACAGCAGATGGCAACGCTGAAGAGGGGCAGGCGCAGCCAAGTGAACCCATCGTTAACACCCAGCCGCTGCAGGAAAATGTAAGTG : 416
H27_India-VII_refseq  : CGTCTCCTTCCTGGAGTTCACAGCAGATGGCAACGCTGAAGAGGGGCAGGCGCAGCCAAGTGAACCCATCGTTAACACCCAGCCGCTGCAGGAAAATGTAAGTG : 416
H28_Ctg_refsequence   : CGTCTCCTTCCTGGAGTTCACAGCAGATGGCAACGCTGAAGAGGGGCAGGCGCAGCCAAGTGAACCCATCGTTAACACCCAGCCGCTGCAGGAAAATGTAAGTG : 416
H29_Salvador-I_refse  : CGTCTCCTTCCTGGAGTTCACAGCAGATGGCAACGCTGAAGAGGGGCAGGCGCAGCCAAGTGAACCCATCGTTAACACCCAGCCGCTGCAGGAAAATGTAAGTG : 416
H30_NKorea_refsequen  : CGTCTCCTTCCTGGAGTTCACAGCAGATGGCAACGCTGAAGAGGGGCAGGCGCAGCCAAGTGAACCCATCGTTAACACCCAGCCGCTGCAGGAAAATGTAAGTG : 416
H31_P.vivax01_refseq  : CGTCTCCTTCCTGGAGTTCACAGCAGATGGCAACGCTGAAGAGGGGCAGGCGCAGCCAAGTGAACCCATCGTTAACACCCAGCCGCTGCAGGAAAATGTAAGTG : 416
H32_Brazil-I_refsequ  : CGTCTCCTTCCTGGAGTTCACAGCAGATGGCAACGCTGAAGAGGGGCAGGCGCAGCCAAGTGAACCCATCGTTAACACCCAGCCGCTGCAGGAAAATGTAAGTG : 416
                        CGTCTCCTTCCTGGAGTTCACAGCAGATGGCAACGCTGAAGAGGGGCAGGCGCAGCCAAGTGAACCCATCGTTAACACCCAGCCGCTGCAGGAAAATGTAAGTG

                         420         *       440         *       460         *       480         *       500         *       520
H1_VCG                : AAGCCGCTCCCCACTCGGAGGAGTCCACTGGGGGAAGCGCGGAGAAGGGAAGTGGCGGCCACGAGGAAACCCCCCACGCAGGAGAACATGACAAAGGTAGTGGC : 520
H2                    : AAGCCGCTCCCCACTCGGAGGAGTCCACTGGGGGAAGCGCGGAGAAGGGAAGTGGCGGCCACGAGGAAACCCCCCACGCAGGAGAACATGACAAAGGTAGTGGC : 520
```

```
H3                     : AAGCCGCTCCCCACTCGGAGGAGTCCACTGGGGGAAGCGCGGAGAAGGGAAGTGGCGGCCACGAGGAAACCCCCCACGCAGGAGAACATGACAAAGGTAGTGGC :  520
H4                     : AAGCCGCTCCCCACTCGGAGGAGTCCACTGGGGGAAGCGCGGAGAAGGGAAGTGGCGGCCACGAGGAAACCCCCCACGCAGGAGAACATGACAAAGGTAGTGGC :  520
H5                     : AAGCCGCTCCCCACTCGGAGGAGTCCACTGGGGGAAGCGCGGAGAAGGGAAGTGGCGGCCACGAGGAAACCCCCCACGCAGGAGAACATGACAAAGGTAGTGGC :  520
H6                     : AAGCCGCTCCCCACTCGGAGGAGTCCACTGGGGGAAGCGCGGAGAAGGGAAGTGGCGGCCACGAGGAAACCCCCCACGCAGGAGAACATGACAAAGGTAGTGGC :  520
H7                     : AAGCCGCTCCCCACTCGGAGGAGTCCACTGGGGGAAGCGCGGAGAAGGGAAGTGGCGGCCACGAGGAAACCCCCCACGCAGGAGAACATGACAAAGGTAGTGGC :  520
H8                     : AAGCCGCTCCCCACTCGGAGGAGTCCACTGGGGGAAGCGCGGAGAAGGGAAGTGGCGGCCACGAGGAAACCCCCCACGCAGGAGAACATGACAAAGGTAGTGGC :  520
H9                     : AAGCCGCTCCCCACTCGGAGGAGTCCACTGGGGGAAGCGCGGAGAAGGGAAGTGGCGGCCACGAGGAAACCCCCCACGCAGGAGAACATGACAAAGGTAGTGGC :  503
H10                    : AAGCCGCTCCCCACTCGGAGGAGTCCACTGGGGGAAGCGCGGAGAAGGGAAGTGGCGGCCACGAGGAAACCCCCCACGCAGGAGAACATGACAAAGGTAGTGGC :  520
H11                    : AAGCCGCTCCCCACTCGGAGGAGTCCACTGGGGGAAGCGCGGAGAAGGGAAGTGGCGGCCACGAGGAAACCCCCCACGCAGGAGAACATGACAAAGGTAGTGGC :  520
H12                    : AAGCCGCTCCCCACTCGGAGGAGTCCACTGGGGGAAGCGCGGAGAAGGGAAGTGGCGGCCACGAGGAAACCCCCCACGCAGGAGAACATGACAAAGGTAGTGGC :  520
H13                    : AAGCCGCTCCCCACTCGGAGGAGTCCACTGGGGGAAGCGCGGAGAAGGGAAGTGGCGGCCACGAGGAAACCCCCCACGCAGGAGAACATGACAAAGGTAGTGGC :  520
H14                    : AAGCCGCTCCCCACTCGGAGGAGTCCACTGGGGGAAGCGCGGAGAAGGGAAGTGGCGGCCACGAGGAAACCCCCCACGCAGGAGAACATGACAAAGGTAGTGGC :  520
H15                    : AAGCCGCTCCCCACTCGGAGGAGTCCACTGGGGGAAGCGCGGAGAAGGGAAGTGGCGGCCACGAGGAAACCCCCCACGCAGGAGAACATGACAAAGGTAGTGGC :  520
H16                    : AAGCCGCTCCCCACTCGGAGGAGTCCACTGGGGGAAGCGCGGAGAAGGGAAGTGGCGGCCACGAGGAAACCCCCCACGCAGGAGAACATGACAAAGGTAGTGGC :  520
H17                    : AAGCCGCTCCCCACTCGGAGGAGTCCACTGGGGGAAGCGCGGAGAAGGGAAGTGGCGGCCACGAGGAAACCCCCCACGCAGGAGAACATGACAAAGGTAGTGGC :  520
H18                    : AAGCCGCTCCCCACTCGGAGGAGTCCACTGGGGGAAGCGCGGAGAAGGGAAGTGGCGGCCACGAGGAAACCCCCCACGCAGGAGAACATGACAAAGGTAGTGGC :  520
H19                    : AAGCCGCTCCCCACTCGGAGGAGTCCACTGGGGGAAGCGCGGAGAAGGGAAGTGGCGGCCACGAGGAAACCCCCCACGCAGGAGAACATGACAAAGGTAGTGGC :  520
H20                    : AAGCCGCTCCCCACTCGGAGGAGTCCACTGGGGGAAGCGCGGAGAAGGGAAGTGGCGGCCACGAGGAAACCCCCCACGCAGGAGAACATGACAAAGGTAGTGGC :  520
H21                    : AAGCCGCTCCCCACTCGGAGGAGTCCACTGGGGGAAGCGCGGAGAAGGGAAGTGGCGGCCACGAGGAAACCCCCCACGCAGGAGAACATGACAAAGGTAGTGGC :  520
H22                    : AAGCCGCTCCCCACTCGGAGGAGTCCACTGGGGGAAGCGCGGAGAAGGGAAGTGGCGGCCACGAGGAAACCCCCCACGCAGGAGAACATGACAAAGGTAGTGGC :  520
H23                    : AAGCCGCTCCCCACTCGGAGGAGTCCACTGGGGGAAGCGCGGAGAAGGGAAGTGGCGGCCACGAGGAAACCCCCCACGCAGGAGAACATGACAAAGGTAGTGGC :  520
H24                    : AAGCCGCTCCCCACTCGGAGGAGTCCACTGGGGGAAGCGCGGAGAAGGGAAGTGGCGGCCACGAGGAAACCCCCCACGCAGGAGAACATGACAAAGGTAGTGGC :  520
H25                    : AAGCCGCTCCCCACTCGGAGGAGTCCACTGGGGGAAGCGCGGAGAAGGGAAGTGGCGGCCACGAGGAAACCCCCCACGCAGGAGAACATGACAAAGGTAGTGGC :  520
H26_Mauritania-I_ref   : AAGCCGCTCCCCACTCGGAGGAGTCCACTGGGGGAAGCGCGGAGAAGGGAAGTGGCGGCCACGAGGAAACCCCCCACGCAGGAGAACATGACAAAGGTAGTGGC :  520
H27_India-VII_refseq   : AAGCCGCTCCCCACTCGGAGGAGTCCACTGGGGGAAGCGCGGAGAAGGGAAGTGGCGGCCACGAGGAAACCCCCCACGCAGGAGAACGTGACAAAGGTAGTGGC :  520
H28_Ctg_refsequence    : AAGCCGCTCCCCACTCGGAGGAGTCCACTGGGGGAAGCGCGGAGAAGGGAAGTGGCGGCCACGAGGAAACCCCCCACGCAGGAGAACATGACAAAGGTAGTGGC :  520
H29_Salvador-I_refse   : AAGCCGCTCCCCACTCGGAGGAGTCCACTGGGGGAAGCGCGGAGAAGGGAAGTGGCGGCCACGAGGAAACCCCCCACGCAGGAGAACATGACAAAGGTAGTGGC :  520
H30_NKorea_refsequen   : AAGCCGCTCCCCACTCGGAGGAGTCCACTGGGGGAAGCGCGGAGAAGGGAAGTGGCGGCCACGAGGAAACCCCCCACGCAGGAGAACATGACAAAGGTAGTGGC :  520
H31_P.vivax01_refseq   : AAGCCGCTCCCCACTCGGAGGAGTCCACTGGGGGAAGCGCGGAGAAGGGAAGTGGCGGCCACGAGGAAACCCCCCACGCAGGAGAACATGACAAAGGTAGTGGC :  520
H32_Brazil-I_refsequ   : AAGCCGCTCCCCACTCGGAGGAGTCCACTGGGGGAAGCGCGGAGAAGGGAAGTGGCGGCCACGAGGAAACCCCCCACGCAGGAGAACATGACAAAGGTAGTGGC :  520
                         AAGCCGCTCCCCACTCGGAGGAGTCCACTGGGGGAAGCGCGGAGAAGGGAAGTGGCGGCCACGAGGAAACCCCCCACGCAGGAGAACaTGACAAAGGTAGTGGC
```

```
                                   *      540         *      560         *      580         *      600         *      620
H1_VCG                 : GGAAGCAACGGGAGCAGCGAAAGCAACGGAAGCGTCTTGGCTGATGGCCACGCCGAAGAAGCGCCGAACGAGCAGGTGAGTAGCGAGAAGAACGACGGACACAG :  624
H2                     : GGAAGCAACGGGAGCAGCGAAAGCAACGGAAGCGTCTTGGCTGATGGCCACGCCGAAGAAGCGCCGAACGAGCAGGTGAGTAGCGAGAAGAACGACGGACACAG :  624
H3                     : GGAAGCAACGGGAGCAGCGAAAGCAACGGAAGCGTCTTGGCTGATGGCCACGCCGAAGAAGCGCCGAACGAGCAGGTGAGTAGCGAGAAGAACGACGGACACAG :  624
H4                     : GGAAGCAACGGGAGCAGCGAAAGCAACGGAAGCGTCTTGGCTGATGGCCACGCCGAAGAAGCGCCGAACGAGCAGGTGAGTAGCGAGAAGAACGACGGACACAG :  624
H5                     : GGAAGCAACGGGAGCAGCGAAAGCAACGGAAGCGTCTTGGCTGATGGCCACGCCGAAGAAGCGCCGAACGAGCAGGTGAGTAGCGAGAAGAACGACGGACACAG :  624
H6                     : GGAAGCAACGGGAGCAGCGAAAGCAACGGAAGCGTCTTGGCTGATGGCCACGCCGAAGAAGCGCCGAACGAGCAGGTGAGTAGCGAGAAGAACGACGGACACAG :  624
H7                     : GGAAGCAACGGGAGCAGCGAAAGCAACGGAAGCGTCTTGGCTGATGGCCACGCCGAAGAAGCGCCGAACGAGCAGGTGAGTAGCGAGAAGAACGACGGACACAG :  624
H8                     : GGAAGCAACGGGAGCAGCGAAAGCAACGGAAGCGTCTTGGCTGATGGCCACGCCGAAGAAGCGCCGAACGAGCAGGTGAGTAGCGAGAAGAACGACGGACACAG :  624
H9                     : GGAAGCAACGGGAGCAGCGAAAGCAACGGAAGCGTCTTGGCTGATGGCCACGCCGAAGAAGCGCCGAACGAGCAGGTGAGTAGCGAGAAGAACGACGGACACAG :  607
H10                    : GGAAGCAACGGGAGCAGCGAAAGCAACGGAAGCGTCTTGGCTGATGGCCACGCCGAAGAAGCGCCGAACGAGCAGGTGAGTAGCGAGAAGAACGACGGACACAG :  624
H11                    : GGAAGCAACGGGAGCAGCGAAAGCAACGGAAGCGTCTTGGCTGATGGCCACGCCGAAGAAGCGCCGAACGAGCAGGTGAGTAGCGAGAAGAACGACGGACACAG :  624
H12                    : GGAAGCAACGGGAGCAGCGAAAGCAACGGAAGCGTCTTGGCTGATGGCCACGCCGAAGAAGCGCCGAACGAGCAGGTGAGTAGCGAGAAGAACGACGGACACAG :  624
H13                    : GGAAGCAACGGGAGCAGCGAAAGCAACGGAAGCGTCTTGGCTGATGGCCACGCCGAAGAAGCGCCGAACGAGCAGGTGAGTAGCGAGAAGAACGACGGACACAG :  624
H14                    : GGAAGCAACGGGAGCAGCGAAAGCAACGGAAGCGTCTTGGCTGATGGCCACGCCGAAGAAGCGCCGAACGAGCAGGTGAGTAGCGAGAAGAACGACGGACACAG :  624
H15                    : GGAAGCAACGGGAGCAGCGAAAGCAACGGAAGCGTCTTGGCTGATGGCCACGCCGAAGAAGCGCCGAACGAGCAGGTGAGTAGCGAGAAGAACGACGGACACAG :  624
H16                    : GGAAGCAACGGGAGCAGCGAAAGCAACGGAAGCGTCTTGGCTGATGGCCACGCCGAAGAAGCGCCGAACGAGCAGGTGAGTAGCGAGAAGAACGACGGACACAG :  624
H17                    : GGAAGCAACGGGAGCAGCGAAAGCAACGGAAGCGTCTTGGCTGATGGCCACGCCGAAGAAGCGCCGAACGAGCAGGTGAGTAGCGAGAAGAACGACGGACACAG :  624
```

```
H18                   : GGAAGCAACGGGAGCAGCGAAAGCAACGGAAGCGTCTTGGCTGATGGCCACGCCGAAGAAGCGCCGAACGAGCAGGTGAGTAGCGAGAAGAACGACGGACACAG : 624
H19                   : GGAAGCAACGGGAGCAGCGAAAGCAACGGAAGCGTCTTGGCTGATGGCCACGCCGAAGAAGCGCCGAACGAGCAGGTGAGTAGCGAGAAGAACGACGGACACAG : 624
H20                   : GGAAGCAACGGGAGCAGCGAAAGCAACGGAAGCGTCTTGGCTGATGGCCACGCCGAAGAAGCGCCGAACGAGCAGGTGAGTAGCGAGAAGAACGACGGACACAG : 624
H21                   : GGAAGCAACGGGAGCAGCGAAAGCAACGGAAGCGTCTTGGCTGATGGCCACGCCGAAGAAGCGCCGAACGAGCAGGTGAGTAGCGAGAAGAACGACGGACACAG : 624
H22                   : GGAAGCAACGGGAGCAGCGAAAGCAACGGAAGCGTCTTGGCTGATGGCCACGCCGAAGAAGCGCCGAACGAGCAGGTGAGTAGCGAGAAGAACGACGGACACAG : 624
H23                   : GGAAGCAACGGGAGCAGCGAAAGCAACGGAAGCGTCTTGGCTGATGGCCACGCCGAAGAAGCGCCGAACGAGCAGGTGAGTAGCGAGAAGAACGACGGACACAG : 624
H24                   : GGAAGCAACGGGAGCAGCGAAAGCAACGGAAGCGTCTTGGCTGATGGCCACGCCGAAGAAGCGCCGAACGAGCAGGTGAGTAGCGAGAAGAACGACGGACACAG : 624
H25                   : GGAAGCAACGGGAGCAGCGAAAGCAACGGAAGCGTCTTGGCTGATGGCCACGCCGAAGAAGCGCCGAACGAGCAGGTGAGTAGCGAGAAGAACGACGGACACAG : 624
H26_Mauritania-I_ref  : GG------------------AAGCAACGGAAGCGTCTTGGCTGATGGCCACGCCGAAGAAGCGCCGAACGAGCAGGTGAGTAGCGAGAAGAACGACGGACACAG : 606
H27_India-VII_refseq  : GGAAGCAACGGGAGCAGCGAAAGCAACGGAAGCGTCTTGGCTGATGGCCACGCCGAAGAAGCGCCGAACGAGCAGGTGAGTAGCGAGAAGAACGACGGACACAG : 624
H28_Ctg_refsequence   : GGAAGCAACGGGAGCAGCGAAAGCAACGGAAGCGTCTTGGCTGATGGCCACGCCGAAGAAGCGCCGAACGAGCAGGTGAGTAGCGAGAAGAACGACGGACACAG : 624
H29_Salvador-I_refse  : GGAAGCAACGGGAGCAGCGAAAGCAACGGAAGCGTCTTGGCTGATGGCCACGCCGAAGAAGCGCCGAACGAGCAGGTGAGTAGCGAGAAGAACGACGGACACAG : 624
H30_NKorea_refsequen  : GGAAGCAACGGGAGCAGCGAAAGCAACGGAAGCGTCTTGGCTGATGGCCACGCCGAAGAAGCGCCGAACGAGCAGGTGAGTAGCGAGAAGAACGACGGACACAG : 624
H31_P.vivax01_refseq  : GGAAGCAACGGGAGCAGCGAAAGCAACGGGAGCGTCTTGGCTGATGGCCACGCCGAAGAAGCGCCGAACGAGCAGGTGAGTAGCGAGAAGAACGACGGACACAG : 624
H32_Brazil-I_refsequ  : GGAAGCAACGGGAGCAGCGAAAGCAACGGAAGCGTCTTGGCTGATGGCCACGCCGAAGAAGCGCCGAACGAGCAGGTGAGTAGCGAGAAGAACGACGGACACAG : 624
                        GGaagcaacgggagcagcgaAAGCAACGGaAGCGTCTTGGCTGATGGCCACGCCGAAGAAGCGCCGAACGAGCAGGTGAGTAGCGAGAAGAACGACGGACACAG

                            *       640         *       660         *       680         *       700         *       720
H1_VCG                : TGAAAGCAGCACCACGGGCTCACACTCAGATAAGGAGGAGCTGGACTTGTATACGTCCTCCCCCTCCGATAAAGAAGAAAACAGCGCCCCTGCCATGGGTGGTA : 728
H2                    : TGAAAGCAGCACCACGGGCTCACACTCAGATAAGGAGGAGCTGGACTTGTATACGTCCTCCCCCTCCGATAAAGAAGAAAACAGCGCCCCTGCCATGGGTGGTA : 728
H3                    : TGAAAGCAGCACCACGGGCTCACACTCAGATAAGGAGGAGCTGGACTTGTATACGTCCTCCCCCTCCGATAAAGAAGAAAACAGCGCCCCTGCCATGGGTGGTA : 728
H4                    : TGAAAGCAGCACCACGGGCTCACACTCAGATAAGGAGGAGCTGGACTTGTATACGTCCTCCCCCTCCGATAAAGAAGAAAACAGCGCCCCTGCCATGGGTGGTA : 728
H5                    : TGAAAGCAGCACCACGGGCTCACACTCAGATAAGGAGGAGCTGGACTTGTATACGTCCTCCCCCTCCGATAAAGAAGAAAACAGCGCCCCTGCCATGGGTGGTA : 728
H6                    : TGAAAGCAGCACCACGGGCTCACACTCAGATAAGGAGGAGCTGGACTTGTATACGTCCTCCCCCTCCGATAAAGAAGAAAACAGCGCCCCTGCCATGGGTGGTA : 728
H7                    : TGAAAGCAGCACCACGGGCTCACACTCAGATAAGGAGGAGCTGGACTTGTATACGTCCTCCCCCTCCGATAAAGAAGAAAACAGCGCCCCTGCCATGGGTGGTA : 728
H8                    : TGAAAGCAGCACCACGGGCTCACACTCAGATAAGGAGGAGCTGGACTTGTATACGTCCTCCCCCTCCGATAAAGAAGAAAACAGCGCCCCTGCCATGGGTGGTA : 728
H9                    : TGAAAGCAGCACCACGGGCTCACACTCAGATAAGGAGGAGCTGGACTTGTATACGTCCTCCCCCTCCGATAAAGAAGAAAACAGCGCCCCTGCCATGGGTGGTA : 711
H10                   : TGAAAGCAGCACCACGGGCTCACACTCAGATAAGGAGGAGCTGGACTTGTATACGTCCTCCCCCTCCGATAAAGAAGAAAACAGCGCCCCTGCCATGGGTGGTA : 728
H11                   : TGAAAGCAGCACCACGGGCTCACACTCAGATAAGGAGGAGCTGGACTTGTATACGTCCTCCCCCTCCGATAAAGAAGAAAACAGCGCCCCTGCCATGGGTGGTA : 728
H12                   : TGAAAGCAGCACCACGGGCTCACACTCAGATAAGGAGGAGCTGGACTTGTATACGTCCTCCCCCTCCGATAAAGAAGAAAACAGCGCCCCTGCCATGGGTGGTA : 728
H13                   : TGAAAGCAGCACCACGGGCTCACACTCAGATAAGGAGGAGCTGGACTTGTATACGTCCTCCCCCTCCGATAAAGAAGAAAACAGCGCCCCTGCCATGGGTGGTA : 728
H14                   : TGAAAGCAGCACCACGGGCTCACACTCAGATAAGGAGGAGCTGGACTTGTATACGTCCTCCCCCTCCGATAAAGAAGAAAACAGCGCCCCTGCCATGGGTGGTA : 728
H15                   : TGAAAGCAGCACCACGGGCTCACACTCAGATAAGGAGGAGCTGGACTTGTATACGTCCTCCCCCTCCGATAAAGAAGAAAACAGCGCCCCTGCCATGGGTGGTA : 728
H16                   : TGAAAGCAGCACCACGGGCTCACACTCAGATAAGGAGGAGCTGGACTTGTATACGTCCTCCCCCTCCGATAAAGAAGAAAACAGCGCCCCTGCCATGGGTGGTA : 728
H17                   : TGAAAGCAGCACCACGGGCTCACACTCAGATAAGGAGGAGCTGGACTTGTATACGTCCTCCCCCTCCGATAAAGAAGAAAACAGCGCCCCTGCCATGGGTGGTA : 728
H18                   : TGAAAGCAGCACCACGGGCTCACACTCAGATAAGGAGGAGCTGGACTTGTATACGTCCTCCCCCTCCGATAAAGAAGAAAACAGCGCCCCTGCCATGGGTGGTA : 728
H19                   : TGAAAGCAGCACCACGGGCTCACACTCAGATAAGGAGGAGCTGGACTTGTATACGTCCTCCCCCTCCGATAAAGAAGAAAACAGCGCCCCTGCCATGGGTGGTA : 728
H20                   : TGAAAGCAGCACCACGGGCTCACACTCAGATAAGGAGGAGCTGGACTTGTATACGTCCTCCCCCTCCGATAAAGAAGAAAACAGCGCCCCTGCCATGGGTGGTA : 728
H21                   : TGAAAGCAGCACCACGGGCTCACACTCAGATAAGGAGGAGCTGGACTTGTATACGTCCTCCCCCTCCGATAAAGAAGAAAACAGCGCCCCTGCCATGGGTGGTA : 728
H22                   : TGAAAGCAGCACCACGGGCTCACACTCAGATAAGGAGGAGCTGGACTTGTATACGTCCTCCCCCTCCGATAAAGAAGAAAACAGCGCCCCTGCCATGGGTGGTA : 728
H23                   : TGAAAGCAGCACCACGGGCTCACACTCAGATAAGGAGGAGCTGGACTTGTATACGTCCTCCCCCTCCGATAAAGAAGAAAACAGCGCCCCTGCCATGGGTGGTA : 728
H24                   : TGAAAGCAGCACCACGGGCTCACACTCAGATAAGGAGGAGCTGGACTTGTATACGTCCTCCCCCTCCGATAAAGAAGAAAACAGCGCCCCTGCCATGGGTGGTA : 728
H25                   : TGAAAGCAGCACCACGGGCTCACACTCAGATAAGGAAGAGCTGGACTTGTATACGTCCTCCCCCTCCGATAAAGAAGAAAACAGCGCCCCTGCCATGGGTGGTA : 728
H26_Mauritania-I_ref  : TGAAAGCAGCACCACGGGCTCACACTCAGATAAGGAGGAGCTGGACTTGTATACGTCCTCCCCCTCCGATAAAGAAGAAAACAGCGCCCCTGCCATGGGTGGTA : 710
H27_India-VII_refseq  : TGAAAGCAGCACCACGGGCTCACACTCAGATAAGGAGGAGCTGGACTTGTATACGTCCTCCCCCTCCGATAAAGAAGAAAACAGCGCCCCTGCCATGGGTGGTA : 728
H28_Ctg_refsequence   : TGAAAGCAGCACCACGGGCTCACACTCAGATAAGGAGGAGCTGGACTTGTATACGTCCTCCCCCTCCGATAAAGAAGAAAACAGCGCCCCTGCCATGGGTGGTA : 728
H29_Salvador-I_refse  : TGAAAGCAGCACCACGGGCTCACACTCAGATAAGGAGGAGCTGGACTTGTATACGTCCTCCCCCTCCGATAAAGAAGAAAACAGCGCCCCTGCCATGGGTGGTA : 728
H30_NKorea_refsequen  : TGAAAGCAGCACCACGGGCTCACACTCAGATAAGGAGGAGCTGGACTTGTATACGTCCTCCCCCTCCGATAAAGAAGAAAACAGCGCCCCTGCCATGGGTGGTA : 728
H31_P.vivax01_refseq  : TGAAAGCAGCACCACGGGCTCACACTCAGATAAGGAGGAGCTGGACTTGTATACGTCCTCCCCCTCCGATAAAGAAGAAAACAGCGCCCCTGCCATGGGTGGTA : 728
H32_Brazil-I_refsequ  : TGAAAGCAGCACCACGGGCTCACACTCAGATAAGGAGGAGCTGGACTTGTATACGTCCTCCCCCTCCGATAAAGAAGAAAACAGCGCCCCTGCCATGGGTGGTA : 728
```

```
                           TGAAAGCAGCACCACGGGCTCACACTCAGATAAGGAgGAGCTGGACTTGTATACGTCCTCCCCCTCCGATAAAGAAGAAAACAGCGCCCCTGCCATGGGTGGTA

                            *     740     *     760     *     780     *     800     *     820     *
H1_VCG                    : TGCACAACACGGTAGGCCTGAGGGGGGGAAGCGCCTCTGAATCGGCGGCCCCAAGTGAAGAGAAAGCATCCGAAGAGAATGGCCCCCATGAAGTGGCAGATAAA : 832
H2                        : TGCACAACACGGTAGGCCTGAGGGGGGGAAGCGCCTCTGAATCGGCGGCCCCAAGTGAAGAGAAAGCATCCGAAGAGAATGTCCCCCATGAAGTGGCAGATAAA : 832
H3                        : TGCACAACACGGTAGGCCTGAGGGGGGGAAGCGCCTCTGAATCGGCGGCCCCAAGTGAAGAGAAAGCATCCGAAGAGAATGTCCCCCATGAAGTGGCAGATAAA : 832
H4                        : TGCACAACACGGTAGGCCTGAGGGGGGGAAGCGCCTCTGAATCGGCGGCCCCAAGTGAAGAGAAAGCATCCGAAGAGAATGTCCCCCATGAAGTGGCAGATAAA : 832
H5                        : TGCACAACACGGTAGGCCTGAGGGGGGGAAGCGCCTCTGAATCGGCGGCCCCAAGTGAAGAGAAAGCATCCGAAGAGAATGTCCCCCATGAAGTGGCAGATAAA : 832
H6                        : TGCACAACACGGTAGGCCTGAGGGGGGGAAGCGCCTCTGAATCGGCGGCCCCAAGTGAAGAGAAAGCATCCGAAGAGAATGGCCCCCATGAAGTGGCAGATAAA : 832
H7                        : TGCACAACACGGTAGGCCTGAGGGGGGGAAGCGCCTCTGAATCGGCGGCCCCAAGTGAAGAGAAAGCATCCGAAGAGAATGTCCCCCATGAAGTGGCAGATAAA : 832
H8                        : TGCACAACACGGTAGGCCTGAGGGGGGGAAGCGCCTCTGAATCGGCGGCCCCAAGTGAAGAGAAAGCATCCGAAGAGAATGTCCCCCATGAAGTGGCAGATAAA : 832
H9                        : TGCACAACACGGTAGGCCTGAGGGGGGGAAGCGCCTCTGAATCGGCGGCCCCAAGTGAAGAGAAAGCATCCGAAGAGAATGTCCCCCATGAAGTGGCAGATAAA : 815
H10                       : TGCACAACACGGTAGGCCTGAGGGGGGGAAGCGCCTCTGAATCGGCGGCCCCAAGTGAAGAGAAAGCATCCGAAGAGAATGTCCCCCATGAAGTGGCAGATAAA : 832
H11                       : TGCACAACACGGTAGGCCTGAGGGGGGGAAGCGCCTCTGAATCGGCGGCCCCAAGTGAAGAGAAAGCATCCGAAGAGAATGTCCCCCATGAAGTGGCAGATAAA : 832
H12                       : TGCACAACACGGTAGGCCTGAGGGGGGGAAGCGCCTCTGAATCGGCGGCCCCAAGTGAAGAGAAAGCATCCGAAGAGAATGTCCCCCATGAAGTGGCAGATAAA : 832
H13                       : TGCACAACACGGTAGGCCTGAGGGGGGGAAGCGCCTCTGAATCGGCGGCCCCAAGTGAAGAGAAAGCATCCGAAGAGAATGTCCCCCATGAAGTGGCAGATAAA : 832
H14                       : TGCACAACACGGTAGGCCTGAGGGGGGGAAGCGCCTCTGAATCGGCGGCCCCAAGTGAAGAGAAAGCATCCGAAGAGAATGTCCCCCATGAAGTGGCAGATAAA : 832
H15                       : TGCACAACACGGTAGGCCTGAGGGGGGGAAGCGCCTCTGAATCGGCGGCCCCAAGTGAAGAGAAAGCATCCGAAGAGAATGTCCCCCATGAAGTGGCAGATAAA : 832
H16                       : TGCACAACACGGTAGGCCTGAGGGGGGGAAGCGCCTCTGAATCGGCGGCCCCAAGTGAAGAGAAAGCATCCGAAGAGAATGGCCCCCATGAAGTGGCAGATAAA : 832
H17                       : TGCACAACACGGTAGGCCTGAGGGGGGGAAGCGCCTCTGAATCGGCGGCCCCAAGTGAAGAGAAAGCATCCGAAGAGAATGTCCCCCATGAAGTGGCAGATAAA : 832
H18                       : TGCACAACACGGTAGGCCTGAGGGGGGGAAGCGCCTCTGAATCGGCGGCCCCAAGTGAAGAGAAAGCATCCGAAGAGAATGTCCCCCATGAAGTGGCAGATAAA : 832
H19                       : TGCACAACACGGTAGGCCTGAGGGGGGGAAGCGCCTCTGAATCGGCGGCCCCAAGTGAAGAGAAAGCATCCGAAGAGAATGTCCCCCATGAAGTGGCAGATAAA : 832
H20                       : TGCACAACACGGTAGGCCTGAGGGGGGGAAGCGCCTCTGAATCGGCGGCCCCAAGTGAAGAGAAAGCATCCGAAGAGAATGTCCCCCATGAAGTGGCAGATAAA : 832
H21                       : TGCACAACACGGTAGGCCTGAGGGGGGGAAGCGCCTCTGAATCGGCGGCCCCAAGTGAAGAGAAAGCATCCGAAGAGAATGTCCCCCATGAAGTGGCAGATAAA : 832
H22                       : TGCACAACACGGTAGGCCTGAGGGGGGGAAGCGCCTCTGAATCGGCGGCCCCAAGTGAAGAGAAAGCATCCGAAGAGAATGTCCCCCATGAAGTGGCAGATAAA : 832
H23                       : TGCACAACACGGTAGGCCTGAGGGGGGGAAGCGCCTCTGAATCGGCGGCCCCAAGTGAAGAGAAAGCATCCGAAGAGAATGTCCCCCATGAAGTGGCAGATAAA : 832
H24                       : TGCACAACACGGTAGGCCTGAGGGGGGGAAGCGCCTCTGAATCGGCGGCCCCAAGTGAAGAGAAAGCATCCGAAGAGAATGTCCCCCATGAAGTGGCAGATAAA : 832
H25                       : TGCACAACACGGTAGGCCTGAGGGGGGGAAGCGCCTCTGAATCGGCGGCCCCAAGTGAAGAGAAAGCATCCGAAGAGAATGTCCCCCATGAAGTGGCAGATAAA : 832
H26_Mauritania-I_ref      : TGCACAACACGGTAGGCCTGAGGGGGGGAAGCGCCTCTGAATCGGCGGCCCCAAGTGAAGAGAAAGCATCCGAAGAGAATGTCCCCCATGAAGTGGCAGATAAA : 814
H27_India-VII_refseq      : TGCACAACACGGTAGGCCTGAGGGGGGGAAGCGCCTCTGAATCGGCGGCCCCAAGTGAAGAGAAAGCATCCGAAGAGAATGTCCCCCATGAAGTGGCAGATAAA : 832
H28_Ctg_refsequence       : TGCACAACACGGTAGGCCTGAGGGGGGGAAGCGCCTCTGAATCGGCGGCCCCAAGTGAAGAGAAAGCATCCGAAGAGAATGTCCCCCATGAAGTGGCAGATAAA : 832
H29_Salvador-I_refse      : TGCACAACACGGTAGGCCTGAGGGGGGGAAGCGCCTCTGAATCGGCGGCCCCAAGTGAAGAGAAAGCATCCGAAGAGAATGTCCCCCATGAAGTGGCAGATAAA : 832
H30_NKorea_refsequen      : TGCACAACACGGTAGGCCTGAGGGGGGGAAGCGCCTCTGAATCGGCGGCCCCAAGTGAAGAGAAAGCATCCGAAGAGAATGTCCCCCATGAAGTGGCAGATAAA : 832
H31_P.vivax01_refseq      : TGCACAACACGGTAGGCCTGAGGGGGGGAAGCGCCTCTGAATCGGCGGCCCCAAGTGAAGAGAAAGCATCCGAAGAGAATGTCCCCCATGAAGTGGCAGATAAA : 832
H32_Brazil-I_refsequ      : TGCACAACACGGTAGGCCTGAGGGGGGGAAGCGCCTCTGAATCGGCGGCCCCAAGTGAAGAGAAAGCATCCGAAGAGAATGTCCCCCATGAAGTGGCAGATAAA : 832
                            TGCACAACACGGTAGGCCTGAGGGGGGGAAGCGCCTCTGAATCGGCGGCCCCAAGTGAAGAGAAAGCATCCGAAGAGAATGtCCCCCATGAAGTGGCAGATAAA

                                    840     *     860     *     880     *     900     *     920     *
H1_VCG                    : AATGGGGAGGACACCTCACAGGAGGGTTCCCACGCGGGAGCGCTGTCACCTGCGCATGTGGGTGGCGA---------GAGTGGCGAGAGTGGCGGCGAGCACGG : 927
H2                        : AATGGGGAGGACACCTCACAGGAGGGTTCCCACGCGGGAGCGCTGTCACCTGCGCATGTGGGTGGCGA---------GAGTGGCGAGAGTGGCGGCGAGCACGG : 927
H3                        : AATGGGGAGGACACCTCACAGGAGGGTTCCCACGCGGGAGCGCTGTCACCTGCGCATGTGGGTGGCGA---------GAGTGGCGAGAGTGGCGGCGAGCACGG : 927
H4                        : AATGGGGAGGACACCTCACAGGAGGGTTCCCACGCGGGAGCGCTGTCACCTGCGCATGTGGGTGGCGA---------GAGTGGCGAGAGTGGCGGCGAGCACGG : 927
H5                        : AATGGGGAGGACACCTCACAGGAGGGTTCCCACGCGGGAGCGCTGTCACCTGCGCATGTGGGTGGCGA---------GAGTGGCGAGAGTGGCGGCGAGCACGG : 927
H6                        : AATGGGGAGGACACCTCACAGGAGGGTTCCCACGCGGGAGCGCTGTCACCTGCGCATGTGGGTGGCGA------------------GAGTGGCGGCGAGCACGG : 918
H7                        : AATGGGGAGGACACCTCACAGGAGGGTTCCCACGCGGGAGCGCTGTCACCTGCGCATGTGGGTGGCGAGAGTGGCGAGAGTGGCGAGAGTGGCGGCGAGCACGG : 936
H8                        : AATGGGGAGGACACCTCACAGGAGGGTTCCCACGCGGGAGCGCTGTCACCTGCGCATGTGGGTGGCGAGAGTGGCGAGAGTGGCGAGAGTGGCGGCGAGCACGG : 936
H9                        : AATGGGGAGGACACCTCACAGGAGGGTTCCCACGCGGGAGCGCTGTCACCTGCGCATGTGGGTGGCGA---------GAGTGGCGAGAGTGGCGGCGAGCACGG : 910
H10                       : AATGGGGAGGACACCTCACAGGAGGGTTCCCACGCGGGAGCGCTGTCACCTGCGCATGTGGGTGGCGAGAGTGGCGAGAGTGGCGAGAGTGGCGGCGAGCACGG : 936
H11                       : AATGGGGAGGACACCTCACAGGAGGGTTCCCACGCGGGAGCGCTGTCACCTGCGCATGTGGGTGGCGA---------GAGTGGCGAGAGTGGCGGCGAGCACGG : 927
H12                       : AATGGGGAGGACACCTCACAGGAGGGTTCCCACGCGGGAGCGCTGTCACCTGCGCATGTGGGTGGCGA---------GAGTGGCGAGAGTGGCGGCGAGCACGG : 927
```

```
H13                   : AATGGGGAGGACACCTCACAGGAGGGTTCCCACGCGGGAGCGCTGTCACCTGCGCATGTGGGTGGCGA---------GAGTGGCGAGAGTGGCGGCGAGCACGG :  927
H14                   : AATGGGGAGGACACCTCACAGGAGGGTTCCCACGCGGGAGCGCTGTCACCTGCGCATGTGGGTGGCGA---------GAGTGGCGAGAGTGGCGGCGAGCACGG :  927
H15                   : AATGGGGAGGACACCTCACAGGAGGGTTCCCACGCGGGAGCGCTGTCACCTGCGCATGTGGGTGGCGA---------GAGTGGCGAGAGTGGCGGCGAGCACGG :  927
H16                   : AATGGGGAGGACACCTCACAGGAGGGTTCCCACGCGGGAGCGCTGTCACCTGCGCATGTGGGTGGCGA---------GAGTGGCGAGAGTGGCGGCGAGCACGG :  927
H17                   : AATGGGGAGGACACCTCACAGGAGGGTTCCCACGCGGGAGCGCTGTCACCTGCGCATGTGGGTGGCGA---------GAGTGGCGAGAGTGGCGGCGAGCACGG :  927
H18                   : AATGGGGAGGACACCTCACAGGAGGGTTCCCACGCGGGAGCGCTGTCACCTGCGCATGTGGGTGGCGA---------GAGTGGCGAGAGTGGCGGCGAGCACGG :  927
H19                   : AATGGGGAGGACACCTCACAGGAGGGTTCCCACGCGGGAGCGCTGTCACCTGCGCATGTGGGTGGCGA---------GAGTGGCGAGAGTGGCGGCGAGCACGG :  927
H20                   : AATGGGGAGGACACCTCACAGGAGGGTTCCCACGCGGGAGCGCTGTCACCTGCGCATGTGGGTGGCGA---------GAGTGGCGAGAGTGGCGGCGAGCACGG :  927
H21                   : AATGGGGAGGACACCTCACAGGAGGGTTCCCACGCGGGAGCGCTGTCACCTGCGCATGTGGGTGGCGA---------GAGTGGCGAGAGTGGCGGCGAGCACGG :  927
H22                   : AATGGGGAGGACACCTCACAGGAGGGTTCCCACGCGGGAGCGCTGTCACCTGCGCATGTGGGTGGCGA---------GAGTGGCGAGAGTGGCGGCGAGCACGG :  927
H23                   : AATGGGGAGGACACCTCACAGGAGGGTTCCCACGCGGGAGCGCTGTCACCTGCGCATGTGGGTGGCGA---------GAGTGGCGAGAGTGGCGGCGAGCACGG :  927
H24                   : AATGGGGAGGACACCTCACAGGAGGGTTCCCACGCGGGAGCGCTGTCACCTGCGCATGTGGGTGGCGAGAGTGGCGAGAGTGGCGAGAGTGGCGGCGAGCACGG :  936
H25                   : AATGGGGAGGACACCTCACAGGAGGGTTCCCACGCGGGAGCGCTGTCACCTGCGCATGTGGGTGGCGA---------GAGTGGCGAGAGTGGCGGCGAGCACGG :  927
H26_Mauritania-I_ref  : AATGGGGAGGACACCTCACAGGAGGGTTCCCACGCGGGAGCGCTGTCACCTGCGCATGTGGGTGGCGA------------------GAGTGGCGGCGAGCACGG :  900
H27_India-VII_refseq  : AATGGGGAGGACACCTCACAGGAGGGTTCCCACGCGGGAGCGCTGTCACCTGCGCATGTGGGTGGCGA------------------GAGTGGCGGCGAGCACGG :  918
H28_Ctg_refsequence   : AATGGGGAGGACACCTCACAGGAGGGTTCCCACGCGGGAGCGCTGTCACCTGCGCATGTGGGTGGCGA---------GAGTGGCGAGAGTGGCGGCGAGCACGG :  927
H29_Salvador-I_refse  : AATGGGGAGGACACCTCACAGGAGGGTTCCCACGCGGGAGCGCTGTCACCTGCGCATGTGGGTGGCGA---------GAGTGGCGAGAGTGGCGGCGAGCACGG :  927
H30_NKorea_refsequen  : AATGGGGAGGACACCTCACAGGAGGGTTCCCACGCGGGAGCGCTGTCACCTGCGCATGTGGGTGGCGA------------------GAGTGGCGGCGAGCACGG :  918
H31_P.vivax01_refseq  : AATGGGGAGGACACCTCACAGGAGGGTTCCCACGCGGGAGCGCTGTCACCTGCGCATGTGGGTGGCGA------------------GAGTGGCGGCGAGCACGG :  918
H32_Brazil-I_refsequ  : AATGGGGAGGACACCTCACAGGAGGGTTCCCACGCGGGAGCGCTGTCACCTGCGCATGTGGGTGGCGA---------GAGTGGCGAGAGTGGCGGCGAGCACGG :  927
                        AATGGGGAGGACACCTCACAGGAGGGTTCCCACGCGGGAGCGCTGTCACCTGCGCATGTGGGTGGCGA         gagtggcgaGAGTGGCGGCGAGCACGG

                           940         *        960         *        980         *       1000         *       1020         *      1040
H1_VCG                : AGAGCACGGTGAACACGCTGAACATGGGGAGCACGGAGAGCACGGTGAACATGGCGAGCACGGTGAACA----------------------------------- :  996
H2                    : AGAGCACGGTGAACACGCTGAACATGGGGAGCACGGA------------------GAGCACGGTGAACA----------------CGCTGAACATGGGGAGCAC :  997
H3                    : AGAGCACGGTGAACACGCTGAACATGGGGAGCACGGA------------------GAGCACGGTGAACA----------------CGCTGAACATGGGGAGCAC :  997
H4                    : AGAGCACGGTGAACACGCTGAACATGGGGAGCACGGA------------------GAGCACGGTGAACA----------------CGCTGAACATGGGGAGCAC :  997
H5                    : AGAGCACGGTGAACACGCTGAACATGGGGAGCACGGA------------------GAGCACGGTGAACA----------------CGCTGAACATGGGGAGCAC :  997
H6                    : AGAGCACGGTGAACACGCTGAACATGGGGAGCACGGA------------------GAGCACGGTGAACATGGCGAGCACGGTGAA------------------- :  985
H7                    : AGAGCACGGTGAACACGCTGAACATGGGGAGCACGGAGAGCACGGTGAACATGGCGAGCACGGTGAACA----------------------------------- : 1005
H8                    : AGAGCACGGTGAACACGCTGAACATGGGGAGCACGGA------------------GAGCACGGTGAACA----------------CGCTGAACATGGGGAGCAC : 1006
H9                    : AGAGCACGGTGAACACGCTGAACATGGGGAGCACGGA------------------GAGCACGGTGAACA----------------CGCTGAACATGGGGAGCAC :  980
H10                   : AGAGCACGGTGAACACGCTGAACATGGGGAGCACGGA------------------GAGCACGGTGAACA----------------CGCTGAACATGGGGAGCAC : 1006
H11                   : AGAGCACGGTGAACACGCTGAACATGGGGAGCACGGA------------------GAGCACGGTGAACA----------------CGCTGAACATGGGGAGCAC :  997
H12                   : AGAGCACGGTGAACACGCTGAACATGGGGAGCACGGA------------------GAGCACGGTGAACA----------------CGCTGAACATGGGGAGCAC :  997
H13                   : AGAGCACGGTGAACACGCTGAACATGGGGAGCACGGA------------------GAGCACGGTGAACA----------------CGCTGAACATGGGGAGCAC :  997
H14                   : AGAGCACGGTGAACACGCTGAACATGGGGAGCACGGA------------------GAGCACGGTGAACA----------------CGCTGAACATGGGGAGCAC :  997
H15                   : AGAGCACGGTGAACACGCTGAACATGGGGAGCACGGA------------------GAGCACGGTGAACA----------------CGCTGAACATGGGGAGCAC :  997
H16                   : AGAGCACGGTGAACACGCTGAACATGGGGAGCACGGA------------------GAGCACGGTGAACATGGCGAGCACGGTGAA------------------- :  994
H17                   : AGAGCACGGTGAACACGCTGAACATGGGGAGCACGGA------------------GAGCACGGTGAACA----------------CGCTGAACATGGGGAGCAC :  997
H18                   : AGAGCACGGTGAACACGCTGAACATGGGGAGCACGGA------------------GAGCACGGTGAACATGGCGAGCACGGTGAA------------------- :  994
H19                   : AGAGCACGGTGAACACGCTGAACATGGGGAGCACGGA------------------GAGCACGGTGAACA----------------CGCTGAACATGGGGAGCAC :  997
H20                   : AGAGCACGGTGAACACGCTGAACATGGGGAGCACGGA------------------GAGCACGGTGAACA----------------CGCTGAACATGGGGAGCAC :  997
H21                   : AGAGCACGGTGAACACGCTGAACATGGGGAGCACGGA------------------GAGCACGGTGAACA----------------CGCTGAACATGGGGAGCAC :  997
H22                   : AGAGCACGGTGAACACGCTGAACATGGGGAGCACGGA------------------GAGCACGGTGAACA----------------CGCTGAACATGGGGAGCAC :  997
H23                   : AGAGCACGGTGAACACGCTGAACATGGGGAGCACGGA------------------GAGCACGGTGAACA----------------CGCTGAACATGGGGAGCAC :  997
H24                   : AGAGCACGGTGAACACGCTGAACATGGGGAGCACGGA------------------GAGCACGGTGAACA----------------CGCTGAACATGGGGAGCAC : 1006
H25                   : AGAGCACGGTGAACACGCTGAACATGGGGAGCACGGA------------------GAGCACGGTGAACA----------------CGCTGAACATGGGGAGCAC :  997
H26_Mauritania-I_ref  : AGAGCACGGTGAACACGCTGAACATGGGGAGCACGGA------------------GAGCACGGTGAACA----------------CGCTGAACATGGGGAGCAC :  970
H27_India-VII_refseq  : AGAGCACGGTGAACACGCTGAACATGGGGAGCACGGAGAGCACGGTGAACATGGCGAGCACGGTGAACATGGCGAGCACGGTGAA------------------- : 1003
```

```
H28_Ctg_refsequence  : AGAGCACGGTGAACACGCTGAACATGGGGAGCACGGAGAGCACGG----------------------------------------------------------- :  972
H29_Salvador-I_refse : AGAGCACGGTGAACACGCTGAACATGGGGAGCACGGA------------------------------------------------------------------- :  964
H30_NKorea_refsequen : AGAGCACGGTGAACACGCTGAACATGGGGAGCACGGAGAGCACGGT---------------------------------------------------------- :  964
H31_P.vivax01_refseq : ---------TGAACACGCTGAACATGGGGAGCACGGAGAGCACGGTGAACA----------------------------------CGCTGAACATGGGGAGCAC :  979
H32_Brazil-I_refsequ : AGAGCACGGTGAACACGCTGAACATGGGGAGCACGGAGAGCACGGTGAACATGGCGAGCACGGTGAACA----------------------------------- :  996
                       agagcacggTGAACACGCTGAACATGGGGAGCACGGA                  gagcacggtgaaca

                                *      1060         *      1080         *      1100         *      1120         *      1140
H1_VCG               : -------------------------------------------------------------------------------------------------------- :    -
H2                   : GGAGAGCACGGTGAA----------------------------------------------------------------------------------------- : 1012
H3                   : GGAGAGCACGGTGAA----------------------------------------------------------------------------------------- : 1012
H4                   : GGAGAGCACGGTGAA----------------------------------------------------------------------------------------- : 1012
H5                   : GGAGAGCACGGTGAA----------------------------------------------------------------------------------------- : 1012
H6                   : -------------------------------------------------------------------------------------------------------- :    -
H7                   : -------------------------------------------------------------------------------------------------------- :    -
H8                   : GGAGAGCACGGTGAACAT-------------------------------------------------------------------------------------- : 1024
H9                   : GGAGAGCACGGTGAA----------------------------------------------------------------------------------------- :  995
H10                  : GGAGAGCACGGTGAA----------------------------------------------------------------------------------------- : 1021
H11                  : GGAGAGCACGGTGAA----------------------------------------------------------------------------------------- : 1012
H12                  : GGAGAGCACGGTGAACAT-------------------------------------------------------------------------------------- : 1015
H13                  : GGAGAGCACGGTGAA----------------------------------------------------------------------------------------- : 1012
H14                  : GGAGAGCACGGTGAA----------------------------------------------------------------------------------------- : 1012
H15                  : GGAGAGCACGGTGAA----------------------------------------------------------------------------------------- : 1012
H16                  : -------------------------------------------------------------------------------------------------------- :    -
H17                  : GGAGAGCACGGTGAA---------------------------------------------------------------------------------CACGCTGA : 1020
H18                  : -------------------------------------------------------------------------------------------------------- :    -
H19                  : GGAGAGCACGGTGAA----------------------------------------------------------------------------------------- : 1012
H20                  : GGAGAGCACGGTGAA----------------------------------------------------------------------------------------- : 1012
H21                  : GGAGAGCACGGTGAA----------------------------------------------------------------------------------------- : 1012
H22                  : GGAGAGCACGGTGAA----------------------------------------------------------------------------------------- : 1012
H23                  : GGAGAGCACGGTGAA----------------------------------------------------------------------------------------- : 1012
H24                  : GGAGAGCACGGTGAA----------------------------------------------------------------------------------------- : 1021
H25                  : GGAGAGCACGGTGAA----------------------------------------------------------------------------------------- : 1012
H26_Mauritania-I_ref : GGAGAGCACGGTGAACATGGTGAGCATGGTGAGCACGGTGAGCATGGTGAGCATGGTGAGCACGGTGAACATGGTGAGCATGGGGAGCACGGTGAG-------- : 1066
H27_India-VII_refseq : -------------------------------------------------------------------------------------------------------- :    -
H28_Ctg_refsequence  : -------------------------------------------------------------------------------------------------------- :    -
H29_Salvador-I_refse : -------------------------------------------------------------------------------------------------------- :    -
H30_NKorea_refsequen : -------------------------------------------------------------------------------------------------------- :    -
H31_P.vivax01_refseq : GGAGAGCACGGTGAA----------------------------------------------------------------------------------------- :  994
H32_Brazil-I_refsequ : -------------------------------------------------------------------------------------------------------- :    -

                            *      1160         *      1180         *      1200         *      1220         *      1240
H1_VCG               : -------------------------------------------------------------------------------------------------------- :    -
H2                   : -------------------------------------------------------------------------------------------------------- :    -
H3                   : ----------------------------------------------------------------------------------------------------CACG : 1016
H4                   : -------------------------------------------------------------------------------------------------------- :    -
H5                   : -------------------------------------------------------------------------------------------------------- :    -
H6                   : -------------------------------------------------------------------------------------------------------- :    -
H7                   : -------------------------------------------------------------------------------------------------------- :    -
```

```
H8                   : -------------------------------------------------------------------------------------------------------- :    -
H9                   : ----------------------------------------------------------------CACGCTGAACATGGGGAGCACGGAGAGCACGGTGAACACG : 1035
H10                  : -------------------------------------------------------------------------------------------------------- :    -
H11                  : -------------------------------------------------------------------------------------------------------- :    -
H12                  : -------------------------------------------------------------------------------------------------------- :    -
H13                  : ----------------------------------------------------------------------------------------------------CACG : 1016
H14                  : -------------------------------------------------------------------------------------------------------- :    -
H15                  : -------------------------------------------------------------------------------------------------------- :    -
H16                  : -------------------------------------------------------------------------------------------------------- :    -
H17                  : ACATGGGGAGCACGGAGAGCACGGTGAACACGCTGAACATGGGGAGCACGGAGAGCACGGTGAACACGCTGAACATGGGGAGCACGGAGAGCACGGTGAACACG : 1124
H18                  : -------------------------------------------------------------------------------------------------------- :    -
H19                  : -------------------------------------------------------------------------------------------------------- :    -
H20                  : -------------------------------------------------------------------------------------------------------- :    -
H21                  : -------------------------------------------------------------------------------------------------------- :    -
H22                  : -------------------------------------------------------------------------------------------------------- :    -
H23                  : -------------------------------------------------------------------------------------------------------- :    -
H24                  : -------------------------------------------------------------------------------------------------------- :    -
H25                  : -------------------------------------------------------------------------------------------------------- :    -
H26_Mauritania-I_ref : -------------------------------------------------------------------------------------------------------- :    -
H27_India-VII_refseq : -------------------------------------------------------------------------------------------------------- :    -
H28_Ctg_refsequence  : ---------------------------------------------------------------------------------------AGAGCACGGTGAACACG :  989
H29_Salvador-I_refse : -------------------------------------------------------------------------------GAGCACGGAGAGCACGGTGAACACG :  989
H30_NKorea_refsequen : -------------------------------------------------------------------------------------------------------- :    -
H31_P.vivax01_refseq : -------------------------------------------------------------------------------------------------------- :    -
H32_Brazil-I_refsequ : -------------------------------------------------------------------------------------------------------- :    -

                        *      1260         *      1280         *      1300         *      1320         *      1340         *
H1_VCG               : ----------------------------------------------------------------------------------------TGCTGAACAGGAGGAG : 1012
H2                   : --------------------------------------------------------------------CATGGCGAGCACGGTGAACATGCTGAACAGGAGGAG : 1048
H3                   : CTGAACATGGGGAGCACGGAGAGCACGGTGAACACGCTGAACATGGGGAGCACGGAGAGCACGGTGAACATGGCGAGCACGGTGAACATGCTGAACAGGAGGAG : 1120
H4                   : --------------------------------CACGCTGAACATGGGGAGCACGGAGAGCACGGTGAACATGGCGAGCACGGTGAACATGCTGAACAGGAGGAG : 1084
H5                   : --------------------------------CACGCTGAACATGGGGAGCACGGAGAGCACGGTGAACATGGCGAGCACGGTGAACATGCTGAACAGGAGGAG : 1084
H6                   : --------------------------------------------------------------------------------------CATGCTGAACAGGAGGAG : 1003
H7                   : ----------------------------------------------------------------------------------------TGCTGAACAGGAGGAG : 1021
H8                   : -----------------------------------------------------------------------GGCGAGCACGGTGAACATGCTGAACAGGAGGAG : 1057
H9                   : CTGAACATGGGGAGCACGGAGAGCACGGTGAACACGCTGAACATGGGGAGCACGGAGAGCACGGTGAACATGGCGAGCACGGTGAACATGCTGAACAGGAGGAG : 1139
H10                  : --------------------------------------------------------------------CATGGCGAGCACGGTGAACATGCTGAACAGGAGGAG : 1057
H11                  : --------------------------------CACGCTGAACATGGGGAGCACGGAGA------------------GCACGGTGAACATGCTGAACAGGAGGAG : 1066
H12                  : -----------------------------------------------------------------------GGCGAGCACGGTGAACATGCTGAACAGGAGGAG : 1048
H13                  : CTGAACATGGGGAGCACGGAGAGCACGGTGAACACGCTGAACATGGGGAGCACGGAGAGCACGGTGAACATGGCGAGCACGGTGAACATGCTGAACAGGAGGAG : 1120
H14                  : --------------------------------CACGCTGAACATGGGGAGCACGGAGA------------------GCACGGTGAACATGCTGAACAGGAGGAG : 1066
H15                  : --------------------------------------------------------------------CATGGCGAGCACGGTGAACATGCTGAACAGGAGGAG : 1048
H16                  : --------------------------------------------------------------------------------------CATGCTGAACAGGAGGAG : 1012
H17                  : CTGAACATGGGGAGCACGGAGAGCACGGTGAACACGCTGAACATGGGGAGCACGGAGAGCACGGTGAACATGGCGAGCACGGTGAACATGCTGAACAGGAGGAG : 1228
H18                  : --------------------------------------------------------------------------------------CATGCTGAACAGGAGGAG : 1012
H19                  : --------------------------------CACGCTGAACATGGGGAGCACGGAGAGCACGGTGAA------------------CATGCTGAACAGGAGGAG : 1066
H20                  : --------------------------------------------------------------------CATGGCGAGCACGGTGAACATGCTGAACAGGAGGAG : 1048
H21                  : --------------------------------CACGCTGAACATGGGGAGCACGGAGAGCACGGTGAACATGGCGAGCACGGTGAACATGCTGAACAGGAGGAG : 1084
H22                  : --------------------------------------------------------------------CATGGCGAGCACGGTGAACATGCTGAACAGGAGGAG : 1048
```

```
H23                    : --------------------------------CACGCTGAACATGGGGAGCACGGAGAGCACGGTGAACATGGCGAGCACGGTGAACATGCTGAACAGGAGGAG : 1084
H24                    : --------------------------------CACGCTGAACATGGGGAGCACGGAGAGCACGGTGAACATGGCGAGCACGGTGAACATGCTGAACAGGAGGAG : 1093
H25                    : --------------------------------------------------------------------CATGGCGAGCACGGTGAACATGCTGAACAGGAGGAG : 1048
H26_Mauritania-I_ref   : --------------------------------CACGCTGAACATGGGGAGCACGGAGAGCACGGTGAACATGGCGAGCACGGTGAACATGCTGAACAGGAGGAG : 1138
H27_India-VII_refseq   : --------------------------------------------------------------------------------------CATGCTGAACAGGAGGAG : 1021
H28_Ctg_refsequence    : CTGAACATGGGGAGCACGGAGAGCACGGTGAACACGCTGAACATGGGGAGCACGGAGAGCACGGTGAACATGGCGAGCACGGTGAACATGCTGAACAGGAGGAG : 1093
H29_Salvador-I_refse   : CTGAACATGGGGAGCACGGAGAGCACGGTGAACACGCTGAACATGGGGAGCACGGAGAGCACGGTGAACATGGCGAGCACGGTGAACATGCTGAACAGGAGGAG : 1093
H30_NKorea_refsequen   : -----------------------------------------------------------------GAACATGGCGAGCACGGTGAACATGCTGAACAGGAGGAG : 1003
H31_P.vivax01_refseq   : --------------CATGGCGAGCACGGTGAACACGCTGAACATGGGGAGCACGGAGAGCACGGTGAACATGGCGAGCACGGTGAACATGCTGAACAGGAGGAG : 1084
H32_Brazil-I_refsequ   : ----------------------------------------------------------------------------------------TGCTGAACAGGAGGAG : 1012
                                                                                                               caTGCTGAACAGGAGGAG

                            1360         *       1380         *       1400         *       1420         *       1440         *
H1_VCG                 : CACCAAATCTATGGGACGATTCACCCCGAGGCGGAAGCCTCCATTTTGCCCGCGCTGAAGGAAAAGGGCATCCACGTGTCCAGCCCAGAAAGGGTAATCCTCGA : 1116
H2                     : CACCAAATCTATGGGACGATTCACCCCGAGGCGGAAGCCTCCATTTTGCCCGCGCTGAAGGAAAAGGGCATCCACGTGTCCAGCCCAGAAAGGGTAATCCTCGA : 1152
H3                     : CACCAAATCTATGGGACGATTCACCCCGAGGCGGAAGCCTCCATTTTGCCCGCGCTGAAGGAAAAGGGCATCCACGTGTCCAGCCCAGAAAGGGTAATCCTCGA : 1224
H4                     : CACCAAATCTATGGGACGATTCACCCCGAGGCGGAAGCCTCCATTTTGCCCGCGCTGAAGGAAAAGGGCATCCACGTGTCCAGCCCAGAAAGGGTAATCCTCGA : 1188
H5                     : CACCAAATCTATGGGACGATTCACCCCGAGGCGGAAGCCTCCATTTTGCCCGCGCTGAAGGAAAAGGGCATCCACGTGTCCAGCCCAGAAAGGGTAATCCTCGA : 1188
H6                     : CACCAAATCTATGGGACGATTCACCCCGAGGCGGAAGCCTCCATTTTGCCCGCGCTGAAGGAAAAGGGCATCCACGTGTCCAGCCCAGAAAGGGTAATCCTCGA : 1107
H7                     : CACCAAATCTATGGGACGATTCACCCCGAGGCGGAAGCCTCCATTTTGCCCGCGCTGAAGGAAAAGGGCATCCACGTGTCCAGCCCAGAAAGGGTAATCCTCGA : 1125
H8                     : CACCAAATCTATGGGACGATTCACCCCGAGGCGGAAGCCTCCATTTTGCCCGCGCTGAAGGAAAAGGGCATCCACGTGTCCAGCCCAGAAAGGGTAATCCTCGA : 1161
H9                     : CACCAAATCTATGGGACGATTCACCCCGAGGCGGAAGCCTCCATTTTGCCCGCGCTGAAGGAAAAGGGCATCCACGTGTCCAGCCCAGAAAGGGTAATCCTCGA : 1243
H10                    : CACCAAATCTATGGGACGATTCACCCCGAGGCGGAAGCCTCCATTTTGCCCGCGCTGAAGGAAAAGGGCATCCACGTGTCCAGCCCAGAAAGGGTAATCCTCGA : 1161
H11                    : CACCAAATCTATGGGACGATTCACCCCGAGGCGGAAGCCTCCATTTTGCCCGCGCTGAAGGAAAAGGGCATCCACGTGTCCAGCCCAGAAAGGGTAATCCTCGA : 1170
H12                    : CACCAAATCTATGGGACGATTCACCCCGAGGCGGAAGCCTCCATTTTGCCCGCGCTGAAGGAAAAGGGCATCCACGTGTCCAGCCCAGAAAGGGTAATCCTCGA : 1152
H13                    : CACCAAATCTATGGGACGATTCACCCCGAGGCGGAAGCCTCCATTTTGCCCGCGCTGAAGGAAAAGGGCATCCACGTGTCCAGCCCAGAAAGGGTAATCCTCGA : 1224
H14                    : CACCAAATCTATGGGACGATTCACCCCGAGGCGGAAGCCTCCATTTTGCCCGCGCTGAAGGAAAAGGGCATCCACGTGTCCAGCCCAGAAAGGGTAATCCTCGA : 1170
H15                    : CACCAAATCTATGGGACGATTCACCCCGAGGCGGAAGCCTCCATTTTGCCCGCGCTGAAGGAAAAGGGCATCCACGTGTCCAGCCCAGAAAGGGTAATCCTCGA : 1152
H16                    : CACCAAATCTATGGGACGATTCACCCCGAGGCGGAAGCCTCCATTTTGCCCGCGCTGAAGGAAAAGGGCATCCACGTGTCCAGCCCAGAAAGGGTAATCCTCGA : 1116
H17                    : CACCAAATCTATGGGACGATTCACCCCGAGGCGGAAGCCTCCATTTTGCCCGCGCTGAAGGAAAAGGGCATCCACGTGTCCAGCCCAGAAAGGGTAATCCTCGA : 1332
H18                    : CACCAAATCTATGGGACGATTCACCCCGAGGCGGAAGCCTCCATTTTGCCCGCGCTGAAGGAAAAGGGCATCCACGTGTCCAGCCCAGAAAGGGTAATCCTCGA : 1116
H19                    : CACCAAATCTATGGGACGATTCACCCCGAGGCGGAAGCCTCCATTTTGCCCGCGCTGAAGGAAAAGGGCATCCACGTGTCCAGCCCAGAAAGGGTAATCCTCGA : 1170
H20                    : CACCAAATCTATGGGACGATTCACCCCGAGGCGGAAGCCTCCATTTTGCCCGCGCTGAAGGAAAAGGGCATCCACGTGTCCAGCCCAGAAAGGGTAATCCTCGA : 1152
H21                    : CACCAAATCTATGGGACGATTCACCCCGAGGCGGAAGCCTCCATTTTGCCCGCGCTGAAGGAAAAGGGCATCCACGTGTCCAGCCCAGAAAGGGTAATCCTCGA : 1188
H22                    : CACCAAATCTATGGGACGATTCACCCCGAGGCGGAAGCCTCCATTTTGCCCGCGCTGAAGGAAAAGGGCATCCACGTGTCCAGCCCAGAAAGGGTAATCCTCGA : 1152
H23                    : CACCAAATCTATGGGACGATTCACCCCGAGGCGGAAGCCTCCATTTTGCCCGCGCTGAAGGAAAAGGGCATCCACGTGTCCAGCCCAGAAAGGGTAATCCTCGA : 1188
H24                    : CACCAAATCTATGGGACGATTCACCCCGAGGCGGAAGCCTCCATTTTGCCCGCGCTGAAGGAAAAGGGCATCCACGTGTCCAGCCCAGAAAGGGTAATCCTCGA : 1197
H25                    : CACCAAATCTATGGGACGATTCACCCCGAGGCGGAAGCCTCCATTTTGCCCGCGCTGAAGGAAAAGGGCATCCACGTGTCCAGCCCAGAAAGGGTAATCCTCGA : 1152
H26_Mauritania-I_ref   : CACCAAATCTATGGGACGATTCACCCCGAGGCGGAAGCCTCCATTTTGCCCGCGCTGAAGGAAAAGGGCATCCACGTGTCCAGCCCAGAAAGGGTAATCCTCGA : 1242
H27_India-VII_refseq   : CACCAAATCTATGGGACGATTCACCCCGAGGCGGAAGCCTCCATTTTGCCCGCGCTGAAGGAAAAGGGCATCCACGTGTCCAGCCCAGAAAGGGTAATCCTCGA : 1125
H28_Ctg_refsequence    : CACCAAATCTATGGGACGATTCACCCCGAGGCGGAAGCCTCCATTTTGCCCGCGCTGAAGGAAAAGGGCATCCACGTGTCCAGCCCAGAAAGGGTAATCCTCGA : 1197
H29_Salvador-I_refse   : CACCAAATCTATGGGACGATTCACCCCGAGGCGGAAGCCTCCATTTTGCCCGCGCTGAAGGAAAAGGGCATCCACGTGTCCAGCCCAGAAAGGGTAATCCTCGA : 1197
H30_NKorea_refsequen   : CACCAAATCTATGGGACGATTCACCCCGAGGCGGAAGCCTCCATTTTGCCCGCGCTGAAGGAAAAGGGCATCCACGTGTCCAGCCCAGAAAGGGTAATCCTCGA : 1107
H31_P.vivax01_refseq   : CACCAAATCTATGGGACGATTCACCCCGAGGCGGAAGCCTCCATTTTGCCCGCGCTGAAGGAAAAGGGCATCCACGTGTCCAGCCCAGAAAGGGTAATCCTCGA : 1188
H32_Brazil-I_refsequ   : CACCAAATCTATGGGACGATTCACCCCGAGGCGGAAGCCTCCATTTTGCCCGCGCTGAAGGAAAAGGGCATCCACGTGTCCAGCCCAGAAAGGGTAATCCTCGA : 1116
                         CACCAAATCTATGGGACGATTCACCCCGAGGCGGAAGCCTCCATTTTGCCCGCGCTGAAGGAAAAGGGCATCCACGTGTCCAGCCCAGAAAGGGTAATCCTCGA

                         1460         *       1480         *       1500         *       1520         *       1540         *       1560
H1_VCG                 : GATCATCGAAAGCGCCAAGGAGGGAATAAAAGGATTGCTGAAGCTCCGGAAGAGCAAAGACACAGGCAAGCTGCTGGAGGAGGCACTAGAAAAGCTAAACATAA : 1220
H2                     : GATCATCGAAAGCGCCAAGGAGGGAATAAAAGGATTGCTGAAGCTCCGGAAGAGCAAAGACACAGGCAAGCTGCTGGAGGAGGCACTAGAAAAGCTAAACATAA : 1256
```

```
H3                       : GATCATCGAAAGCGCCAAGGAGGGAATAAAAGGATTGCTGAAGCTCCGGAAGAGCAAAGACACAGGCAAGCTGCTGGAGGAGGCACTAGAAAAGCTAAACATAA : 1328
H4                       : GATCATCGAAAGCGCCAAGGAGGGAATAAAAGGATTGCTGAAGCTCCGGAAGAGCAAAGACACAGGCAAGCTGCTGGAGGAGGCACTAGAAAAGCTAAACATAA : 1292
H5                       : GATCATCGAAAGCGCCAAGGAGGGAATAAAAGGATTGCTGAAGCTCCGGAAGAGCAAAGACACAGGCAAGCTGCTGGAGGAGGCACTAGAAAAGCTAAACATAA : 1292
H6                       : GATCATCGAAAGCGCCAAGGAGGGAATAAAAGGATTGCTGAAGCTCCGGAAGAGCAAAGACACAGGCAAGCTGCTGGAGGAGGCACTAGAAAAGCTAAACATAA : 1211
H7                       : GATCATCGAAAGCGCCAAGGAGGGAATAAAAGGATTGCTGAAGCTCCGGAAGAGCAAAGACACAGGCAAGCTGCTGGAGGAGGCACTAGAAAAGCTAAACATAA : 1229
H8                       : GATCATCGAAAGCGCCAAGGAGGGAATAAAAGGATTGCTGAAGCTCCGGAAGAGCAAAGACACAGGCAAGCTGCTGGAGGAGGCACTAGAAAAGCTAAACATAA : 1265
H9                       : GATCATCGAAAGCGCCAAGGAGGGAATAAAAGGATTGCTGAAGCTCCGGAAGAGCAAAGACACAGGCAAGCTGCTGGAGGAGGCACTAGAAAAGCTAAACATAA : 1347
H10                      : GATCATCGAAAGCGCCAAGGAGGGAATAAAAGGATTGCTGAAGCTCCGGAAGAGCAAAGACACAGGCAAGCTGCTGGAGGAGGCACTAGAAAAGCTAAACATAA : 1265
H11                      : GATCATCGAAAGCGCCAAGGAGGGAATAAAAGGATTGCTGAAGCTCCGGAAGAGCAAAGACACAGGCAAGCTGCTGGAGGAGGCACTAGAAAAGCTAAACATAA : 1274
H12                      : GATCATCGAAAGCGCCAAGGAGGGAATAAAAGGATTGCTGAAGCTCCGGAAGAGCAAAGACACAGGCAAGCTGCTGGAGGAGGCACTAGAAAAGCTAAACATAA : 1256
H13                      : GATCATCGAAAGCGCCAAGGAGGGAATAAAAGGATTGCTGAAGCTCCGGAAGAGCAAAGACACAGGCAAGCTGCTGGAGGAGGCACTAGAAAAGCTAAACATAA : 1328
H14                      : GATCATCGAAAGCGCCAAGGAGGGAATAAAAGGATTGCTGAAGCTCCGGAAGAGCAAAGACACAGGCAAGCTGCTGGAGGAGGCACTAGAAAAGCTAAACATAA : 1274
H15                      : GATCATCGAAAGCGCCAAGGAGGGAATAAAAGGATTGCTGAAGCTCCGGAAGAGCAAAGACACAGGCAAGCTGCTGGAGGAGGCACTAGAAAAGCTAAACATAA : 1256
H16                      : GATCATCGAAAGCGCCAAGGAGGGAATAAAAGGATTGCTGAAGCTCCGGAAGAGCAAAGACACAGGCAAGCTGCTGGAGGAGGCACTAGAAAAGCTAAACATAA : 1220
H17                      : GATCATCGAAAGCGCCAAGGAGGGAATAAAAGGATTGCTGAAGCTCCGGAAGAGCAAAGACACAGGCAAGCTGCTGGAGGAGGCACTAGAAAAGCTAAACATAA : 1436
H18                      : GATCATCGAAAGCGCCAAGGAGGGAATAAAAGGATTGCTGAAGCTCCGGAAGAGCAAAGACACAGGCAAGCTGCTGGAGGAGGCACTAGAAAAGCTAAACATAA : 1220
H19                      : GATCATCGAAAGCGCCAAGGAGGGAATAAAAGGATTGCTGAAGCTCCGGAAGAGCAAAGACACAGGCAAGCTGCTGGAGGAGGCACTAGAAAAGCTAAACATAA : 1274
H20                      : GATCATCGAAAGCGCCAAGGAGGGAATAAAAGGATTGCTGAAGCTCCGGAAGAGCAAAGACACAGGCAAGCTGCTGGAGGAGGCACTAGAAAAGCTAAACATAA : 1256
H21                      : GATCATCGAAAGCGCCAAGGAGGGAATAAAAGGATTGCTGAAGCTCCGGAAGAGCAAAGACACAGGCAAGCTGCTGGAGGAGGCACTAGAAAAGCTAAACATAA : 1292
H22                      : GATCATCGAAAGCGCCAAGGAGGGAATAAAAGGATTGCTGAAGCTCCGGAAGAGCAAAGACACAGGCAAGCTGCTGGAGGAGGCACTAGAAAAGCTAAACATAA : 1256
H23                      : GATCATCGAAAGCGCCAAGGAGGGAATAAAAGGATTGCTGAAGCTCCGGAAGAGCAAAGACACAGGCAAGCTGCTGGAGGAGGCACTAGAAAAGCTAAACATAA : 1292
H24                      : GATCATCGAAAGCGCCAAGGAGGGAATAAAAGGATTGCTGAAGCTCCGGAAGAGCAAAGACACAGGCAAGCTGCTGGAGGAGGCACTAGAAAAGCTAAACATAA : 1301
H25                      : GATCATCGAAAGCGCCAAGGAGGGAATAAAAGGATTGCTGAAGCTCCGGAAGAGCAAAGACACAGGCAAGCTGCTGGAGGAGGCACTAGAAAAGCTAAACATAA : 1256
H26_Mauritania-I_ref     : GATCATCGAAAGCGCCAAGGAGGGAATAAAAGGATTGCTGAAGCTCCGGAAGAGCAAAGACACAGGCAAGCTGCTGGAGGAGGCACTAGAAAAGCTAAACATAA : 1346
H27_India-VII_refseq     : GATCATCGAAAGCGCCAAGGAGGGAATAAAAGGATTGCTGAAGCTCCGGAAGAGCAAAGACACAGGCAAGCTGCTGGAGGAGGCACTAGAAAAGCTAAACATAA : 1229
H28_Ctg_refsequence      : GATCATCGAAAGCGCCAAGGAGGGAATAAAAGGATTGCTGAAGCTCCGGAAGAGCAAAGACACAGGCAAGCTGCTGGAGGAGGCACTAGAAAAGCTAAACATAA : 1301
H29_Salvador-I_refse     : GATCATCGAAAGCGCCAAGGAGGGAATAAAAGGATTGCTGAAGCTCCGGAAGAGCAAAGACACAGGCAAGCTGCTGGAGGAGGCACTAGAAAAGCTAAACATAA : 1301
H30_NKorea_refsequen     : GATCATCGAAAGCGCCAAGGAGGGAATAAAAGGATTGCTGAAGCTCCGGAAGAGCAAAGACACAGGCAAGCTGCTGGAGGAGGCACTAGAAAAGCTAAACATAA : 1211
H31_P.vivax01_refseq     : GATCATCGAAAGCGCCAAGGAGGGAATAAAAGGATTGCTGAAGCTCCGGAAGAGCAAAGACACAGGCAAGCTGCTGGAGGAGGCACTAGAAAAGCTAAACATAA : 1292
H32_Brazil-I_refsequ     : GATCATCGAAAGCGCCAAGGAGGGAATAAAAGGATTGCTGAAGCTCCGGAAGAGCAAAGACACAGGCAAGCTGCTGGAGGAGGCACTAGAAAAGCTAAACATAA : 1220
                           GATCATCGAAAGCGCCAAGGAGGGAATAAAAGGATTGCTGAAGCTCCGGAAGAGCAAAGACACAGGCAAGCTGCTGGAGGAGGCACTAGAAAAGCTAAACATAA

                                      *       1580         *       1600         *       1620         *       1640         *       1660
H1_VCG                   : ACCAAAGGGATCTGCATGCAAACAATAATTTCATTACCCTAGAAATGTATGACCGGATCCTCTCCGAAATGTTTAAAATATTAACAGAAATGTCATTCTATAAG : 1324
H2                       : ACCAAAGGGATCTGCATGCAAACAATAATTTCATTACCCTAGAAATGTATGACCGGATCCTCTCCGAAATGTTTAAAATATTAACAGAAATGTCATTCTATAAG : 1360
H3                       : ACCAAAGGGATCTGCATGCAAACAATAATTTCATTACCCTAGAAATGTATGACCGGATCCTCTCCGAAATGTTTAAAATATTAACAGAAATGTCATTCTATAAG : 1432
H4                       : ACCAAAGGGATCTGCATGCAAACAATAATTTCATTACCCTAGAAATGTATGACCGGATCCTCTCCGAAATGTTTAAAATATTAACAGAAATGTCATTCTATAAG : 1396
H5                       : ACCAAAGGGATCTGCATGCAAACAATAATTTCATTACCCTAGAAATGTATGACCGGATCCTCTCCGAAATGTTTAAAATATTAACAGAAATGTCATTCTATAAG : 1396
H6                       : ACCAAAGGGATCTGCATGCAAACAATAATTTCATTACCCTAGAAATGTATGACCGGATCCTCTCCGAAATGTTTAAAATATTAACAGAAATGTCATTCTATAAG : 1315
H7                       : ACCAAAGGGATCTGCATGCAAACAATAATTTCATTACCCTAGAAATGTATGACCGGATCCTCTCCGAAATGTTTAAAATATTAACAGAAATGTCATTCTATAAG : 1333
H8                       : ACCAAAGGGATCTGCATGCAAACAATAATTTCATTACCCTAGAAATGTATGACCGGATCCTCTCCGAAATGTTTAAAATATTAACAGAAATGTCATTCTATAAG : 1369
H9                       : ACCAAAGGGATCTGCATGCAAACAATAATTTCATTACCCTAGAAATGTATGACCGGATCCTCTCCGAAATGTTTAAAATATTAACAGAAATGTCATTCTATAAG : 1451
H10                      : ACCAAAGGGATCTGCATGCAAACAATAATTTCATTACCCTAGAAATGTATGACCGGATCCTCTCCGAAATGTTTAAAATATTAACAGAAATGTCATTCTATAAG : 1369
H11                      : ACCAAAGGGATCTGCATGCAAACAATAATTTCATTACCCTAGAAATGTATGACCGGATCCTCTCCGAAATGTTTAAAATATTAACAGAAATGTCATTCTATAAG : 1378
H12                      : ACCAAAGGGATCTGCATGCAAACAATAATTTCATTACCCTAGAAATGTATGACCGGATCCTCTCCGAAATGTTTAAAATATTAACAGAAATGTCATTCTATAAG : 1360
H13                      : ACCAAAGGGATCTGCATGCAAACAATAATTTCATTACCCTAGAAATGTATGACCGGATCCTCTCCGAAATGTTTAAAATATTAACAGAAATGTCATTCTATAAG : 1432
H14                      : ACCAAAGGGATCTGCATGCAAACAATAATTTCATTACCCTAGAAATGTATGACCGGATCCTCTCCGAAATGTTTAAAATATTAACAGAAATGTCATTCTATAAG : 1378
H15                      : ACCAAAGGGATCTGCATGCAAACAATAATTTCATTACCCTAGAAATGTATGACCGGATCCTCTCCGAAATGTTTAAAATATTAACAGAAATGTCATTCTATAAG : 1360
H16                      : ACCAAAGGGATCTGCATGCAAACAATAATTTCATTACCCTAGAAATGTATGACCGGATCCTCTCCGAAATGTTTAAAATATTAACAGAAATGTCATTCTATAAG : 1324
H17                      : ACCAAAGGGATCTGCATGCAAACAATAATTTCATTACCCTAGAAATGTATGACCGGATCCTCTCCGAAATGTTTAAAATATTAACAGAAATGTCATTCTATAAG : 1540
```

```
H18                       : ACCAAAGGGATCTGCATGCAAACAATAATTTCATTACCCTAGAAATGTATGACCGGATCCTCTCCGAAATGTTTAAAATATTAACAGAAATGTCATTCTATAAG : 1324
H19                       : ACCAAAGGGATCTGCATGCAAACAATAATTTCATTACCCTAGAAATGTATGACCGGATCCTCTCCGAAATGTTTAAAATATTAACAGAAATGTCATTCTATAAG : 1378
H20                       : ACCAAAGGGATCTGCATGCAAACAATAATTTCATTACCCTAGAAATGTATGACCGGATCCTCTCCGAAATGTTTAAAATATTAACAGAAATGTCATTCTATAAG : 1360
H21                       : ACCAAAGGGATCTGCATGCAAACAATAATTTCATTACCCTAGAAATGTATGACCGGATCCTCTCCGAAATGTTTAAAATATTAACAGAAATGTCATTCTATAAG : 1396
H22                       : ACCAAAGGGATCTGCATGCAAACAATAATTTCATTACCCTAGAAATGTATGACCGGATCCTCTCCGAAATGTTTAAAATATTAACAGAAATGTCATTCTATAAG : 1360
H23                       : ACCAAAGGGATCTGCATGCAAACAATAATTTCATTACCCTAGAAATGTATGACCGGATCCTCTCCGAAATGTTTAAAATATTAACAGAAATGTCATTCTATAAG : 1396
H24                       : ACCAAAGGGATCTGCATGCAAACAATAATTTCATTACCCTAGAAATGTATGACCGGATCCTCTCCGAAATGTTTAAAATATTAACAGAAATGTCATTCTATAAG : 1405
H25                       : ACCAAAGGGATCTGCATGCAAACAATAATTTCATTACCCTAGAAATGTATGACCGGATCCTCTCCGAAATGTTTAAAATATTAACAGAAATGTCATTCTATAAG : 1360
H26_Mauritania-I_ref      : ACCAAAGGGATCTGCATGCAAACAATAATTTCATTACCCTAGAAATGTATGACCGGATCCTCTCCGAAATGTTTAAAATATTAACAGAAATGTCATTCTATAAG : 1450
H27_India-VII_refseq      : ACCAAAGGGATCTGCATGCAAACAATAATTTCATTACCCTAGAAATGTATGACCGGATCCTCTCCGAAATGTTTAAAATATTAACAGAAATGTCATTCTATAAG : 1333
H28_Ctg_refsequence       : ACCAAAGGGATCTGCATGCAAACAATAATTTCATTACCCTAGAAATGTATGACCGGATCCTCTCCGAAATGTTTAAAATATTAACAGAAATGTCATTCTATAAG : 1405
H29_Salvador-I_refse      : ACCAAAGGGATCTGCATGCAAACAATAATTTCATTACCCTAGAAATGTATGACCGGATCCTCTCCGAAATGTTTAAAATATTAACAGAAATGTCATTCTATAAG : 1405
H30_NKorea_refsequen      : ACCAAAGGGATCTGCATGCAAACAATAATTTCATTACCCTAGAAATGTATGACCGGATCCTCTCCGAAATGTTTAAAATATTAACAGAAATGTCATTCTATAAG : 1315
H31_P.vivax01_refseq      : ACCAAAGGGATCTGCATGCAAACAATAATTTCATTACCCTAGAAATGTATGACCGGATCCTCTCCGAAATGTTTAAAATATTAACAGAAATGTCATTCTATAAG : 1396
H32_Brazil-I_refsequ      : ACCAAAGGGATCTGCATGCAAACAATAATTTCATTACCCTAGAAATGTATGACCGGATCCTCTCCGAAATGTTTAAAATATTAACAGAAATGTCATTCTATAAG : 1324
                            ACCAAAGGGATCTGCATGCAAACAATAATTTCATTACCCTAGAAATGTATGACCGGATCCTCTCCGAAATGTTTAAAATATTAACAGAAATGTCATTCTATAAG

                                    *        1680         *        1700         *        1720         *        1740         *        1760
H1_VCG                    : GATGGCCATTTTTACGAAACGTTAGGTTTGAATAAATCTATACTAAATCAATCGCTGAAAGAAATTAAAATAAAAATGTTGAGGACCATTGGAGTGCCATACAC : 1428
H2                        : GATGGCCATTTTTACGAAACGTTAGGTTTGAATAAATCTATACTAAATCAATCGCTGAAAGAAATTAAAATAAAAATGTTGAGGACCATTGGAGTGCCATACAC : 1464
H3                        : GATGGCCATTTTTACGAAACGTTAGGTTTGAATAAATCTATACTAAATCAATCGCTGAAAGAAATTAAAATAAAAATGTTGAGGACCATTGGAGTGCCATACAC : 1536
H4                        : GATGGCCATTTTTACGAAACGTTAGGTTTGAATAAATCTATACTAAATCAATCGCTGAAAGAAATTAAAATAAAAATGTTGAGGACCATTGGAGTGCCATACAC : 1500
H5                        : GATGGCCATTTTTACGAAACGTTAGGTTTGAATAAATCTATACTAAATCAATCGCTGAAAGAAATTAAAATAAAAATGTTGAGGACCATTGGAGTGCCATACAC : 1500
H6                        : GATGGCCATTTTTACGAAACGTTAGGTTTGAATAAATCTATACTAAATCAATCGCTGAAAGAAATTAAAATAAAAATGTTGAGGACCATTGGAGTGCCATACAC : 1419
H7                        : GATGGCCATTTTTACGAAACGTTAGGTTTGAATAAATCTATACTAAATCAATCGCTGAAAGAAATTAAAATAAAAATGTTGAGGACCATTGGAGTGCCATACAC : 1437
H8                        : GATGGCCATTTTTACGAAACGTTAGGTTTGAATAAATCTATACTAAATCAATCGCTGAAAGAAATTAAAATAAAAATGTTGAGGACCATTGGAGTGCCATACAC : 1473
H9                        : GATGGCCATTTTTACGAAACGTTAGGTTTGAATAAATCTATACTAAATCAATCGCTGAAAGAAATTAAAATAAAAATGTTGAGGACCATTGGAGTGCCATACAC : 1555
H10                       : GATGGCCATTTTTACGAAACGTTAGGTTTGAATAAATCTATACTAAATCAATCGCTGAAAGAAATTAAAATAAAAATGTTGAGGACCATTGGAGTGCCATACAC : 1473
H11                       : GATGGCCATTTTTACGAAACGTTAGGTTTGAATAAATCTATACTAAATCAATCGCTGAAAGAAATTAAAATAAAAATGTTGAGGACCATTGGAGTGCCATACAC : 1482
H12                       : GATGGCCATTTTTACGAAACGTTAGGTTTGAATAAATCTATACTAAATCAATCGCTGAAAGAAATTAAAATAAAAATGTTGAGGACCATTGGAGTGCCATACAC : 1464
H13                       : GATGGCCATTTTTACGAAACGTTAGGTTTGAATAAATCTATACTAAATCAATCGCTGAAAGAAATTAAAATAAAAATGTTGAGGACCATTGGAGTGCCATACAC : 1536
H14                       : GATGGCCATTTTTACGAAACGTTAGGTTTGAATAAATCTATACTAAATCAATCGCTGAAAGAAATTAAAATAAAAATGTTGAGGACCATTGGAGTGCCATACAC : 1482
H15                       : GATGGCCATTTTTACGAAACGTTAGGTTTGAATAAATCTATACTAAATCAATCGCTGAAAGAAATTAAAATAAAAATGTTGAGGACCATTGGAGTGCCATACAC : 1464
H16                       : GATGGCCATTTTTACGAAACGTTAGGTTTGAATAAATCTATACTAAATCAATCGCTGAAAGAAATTAAAATAAAAATGTTGAGGACCATTGGAGTGCCATACAC : 1428
H17                       : GATGGCCATTTTTACGAAACGTTAGGTTTGAATAAATCTATACTAAATCAATCGCTGAAAGAAATTAAAATAAAAATGTTGAGGACCATTGGAGTGCCATACAC : 1644
H18                       : GATGGCCATTTTTACGAAACGTTAGGTTTGAATAAATCTATACTAAATCAATCGCTGAAAGAAATTAAAATAAAAATGTTGAGGACCATTGGAGTGCCATACAC : 1428
H19                       : GATGGCCATTTTTACGAAACGTTAGGTTTGAATAAATCTATACTAAATCAATCGCTGAAAGAAATTAAAATAAAAATGTTGAGGACCATTGGAGTGCCATACAC : 1482
H20                       : GATGGCCATTTTTACGAAACGTTAGGTTTGAATAAATCTATACTAAATCAATCGCTGAAAGAAATTAAAATAAAAATGTTGAGGACCATTGGAGTGCCATACAC : 1464
H21                       : GATGGCCATTTTTACGAAACGTTAGGTTTGAATAAATCTATACTAAATCAATCGCTGAAAGAAATTAAAATAAAAATGTTGAGGACCATTGGAGTGCCATACAC : 1500
H22                       : GATGGCCATTTTTACGAAACGTTAGGTTTGAATAAATCTATACTAAATCAATCGCTGAAAGAAATTAAAATAAAAATGTTGAGGACCATTGGAGTGCCATACAC : 1464
H23                       : GATGGCCATTTTTACGAAACGTTAGGTTTGAATAAATCTATACTAAATCAATCGCTGAAAGAAATTAAAATAAAAATGTTGAGGACCATTGGAGTGCCATACAC : 1500
H24                       : GATGGCCATTTTTACGAAACGTTAGGTTTGAATAAATCTATACTAAATCAATCGCTGAAAGAAATTAAAATAAAAATGTTGAGGACCATTGGAGTGCCATACAC : 1509
H25                       : GATGGCCATTTTTACGAAACGTTAGGTTTGAATAAATCTATACTAAATCAATCGCTGAAAGAAATTAAAATAAAAATGTTGAGGACCATTGGAGTGCCATACAC : 1464
H26_Mauritania-I_ref      : GATGGCCATTTTTACGAAACGTTAGGTTTGAATAAATCTATACTAAATCAATCGCTGAAAGAAATTAAAATAAAAATGTTGAGGACCATTGGAGTGCCATACAC : 1554
H27_India-VII_refseq      : GATGGCCATTTTTACGAAACGTTAGGTTTGAATAAATCTATACTAAATCAATCGCTGAAAGAAATTAAAATAAAAATGTTGAGGACCATTGGAGTGCCATACAC : 1437
H28_Ctg_refsequence       : GATGGCCATTTTTACGAAACGTTAGGTTTGAATAAATCTATACTAAATCAATCGCTGAAAGAAATTAAAATAAAAATGTTGAGGACCATTGGAGTGCCATACAC : 1509
H29_Salvador-I_refse      : GATGGCCATTTTTACGAAACGTTAGGTTTGAATAAATCTATACTAAATCAATCGCTGAAAGAAATTAAAATAAAAATGTTGAGGACCATTGGAGTGCCATACAC : 1509
H30_NKorea_refsequen      : GATGGCCATTTTTACGAAACGTTAGGTTTGAATAAATCTATACTAAATCAATCGCTGAAAGAAATTAAAATAAAAATGTTGAGGACCATTGGAGTGCCATACAC : 1419
H31_P.vivax01_refseq      : GATGGCCATTTTTACGAAACGTTAGGTTTGAATAAATCTATACTAAATCAATCGCTGAAAGAAATTAAAATAAAAATGTTGAGGACCATTGGAGTGCCATACAC : 1500
H32_Brazil-I_refsequ      : GATGGCCATTTTTACGAAACGTTAGGTTTGAATAAATCTATACTAAATCAATCGCTGAAAGAAATTAAAATAAAAATGTTGAGGACCATTGGAGTGCCATACAC : 1428
```

```
                          GATGGCCATTTTTACGAAACGTTAGGTTTGAATAAATCTATACTAAATCAATCGCTGAAAGAATTAAAATAAAAATGTTGAGGACCATTGGAGTGCCATACAC

                           *       1780         *       1800         *       1820         *       1840         *       1860         *
H1_VCG                  : GAAGCTACCGCCAATCGTTAAGAACAAAGAGAAGGAAAGCACATGTGCTGCGAACAATCTCATCATTAGCATTACATCAAAAGAGCTAGCTCAACGAATGGCTA : 1532
H2                      : GAAGCTACCGCCAATCGTTAAGAACAAAGAGAAGGAAAGCACATGTGCTGCGAACAATCTCATCATTAGCATTACATCAAAAGAGCTAGCTCAACGAATGGCTA : 1568
H3                      : GAAGCTACCGCCAATCGTTAAGAACAAAGAGAAGGAAAGCACATGTGCTGCGAACAATCTCATCATTAGCATTACATCAAAAGAGCTAGCTCAACGAATGGCTA : 1640
H4                      : GAAGCTACCGCCAATCGTTAAGAACAAAGAGAAGGAAAGCACATGTGCTGCGAACAATCTCATCATTAGCATTACATCAAAAGAGCTAGCTCAACGAATGGCTA : 1604
H5                      : GAAGCTACCGCCAATCGTTAAGAACAAAGAGAAGGAAAGCACATGTGCTGCGAACAATCTCATCATTAGCATTACATCAAAAGAGCTAGCTCAACGAATGGCTA : 1604
H6                      : GAAGCTACCGCCAATCGTTAAGAACAAAGAGAAGGAAAGCACATGTGCTGCGAACAATCTCATCATTAGCATTACATCAAAAGAGCTAGCTCAACGAATGGCTA : 1523
H7                      : GAAGCTACCGCCAATCGTTAAGAACAAAGAGAAGGAAAGCACATGTGCTGCGAACAATCTCATCATTAGCATTACATCAAAAGAGCTAGCTCAACGAATGGCTA : 1541
H8                      : GAAGCTACCGCCAATCGTTAAGAACAAAGAGAAGGAAAGCACATGTGCTGCGAACAATCTCATCATTAGCATTACATCAAAAGAGCTAGCTCAACGAATGGCTA : 1577
H9                      : GAAGCTACCGCCAATCGTTAAGAACAAAGAGAAGGAAAGCACATGTGCTGCGAACAATCTCATCATTAGCATTACATCAAAAGAGCTAGCTCAACGAATGGCTA : 1659
H10                     : GAAGCTACCGCCAATCGTTAAGAACAAAGAGAAGGAAAGCACATGTGCTGCGAACAATCTCATCATTAGCATTACATCAAAAGAGCTAGCTCAACGAATGGCTA : 1577
H11                     : GAAGCTACCGCCAATCGTTAAGAACAAAGAGAAGGAAAGCACATGTGCTGCGAACAATCTCATCATTAGCATTACATCAAAAGAGCTAGCTCAACGAATGGCTA : 1586
H12                     : GAAGCTACCGCCAATCGTTAAGAACAAAGAGAAGGAAAGCACATGTGCTGCGAACAATCTCATCATTAGCATTACATCAAAAGAGCTAGCTCAACGAATGGCTA : 1568
H13                     : GAAGCTACCGCCAATCGTTAAGAACAAAGAGAAGGAAAGCACATGTGCTGCGAACAATCTCATCATTAGCATTACATCAAAAGAGCTAGCTCAACGAATGGCTA : 1640
H14                     : GAAGCTACCGCCAATCGTTAAGAACAAAGAGAAGGAAAGCACATGTGCTGCGAACAATCTCATCATTAGCATTACATCAAAAGAGCTAGCTCAACGAATGGCTA : 1586
H15                     : GAAGCTACCGCCAATCGTTAAGAACAAAGAGAAGGAAAGCACATGTGCTGCGAACAATCTCATCATTAGCATTACATCAAAAGAGCTAGCTCAACGAATGGCTA : 1568
H16                     : GAAGCTACCGCCAATCGTTAAGAACAAAGAGAAGGAAAGCACATGTGCTGCGAACAATCTCATCATTAGCATTACATCAAAAGAGCTAGCTCAACGAATGGCTA : 1532
H17                     : GAAGCTACCGCCAATCGTTAAGAACAAAGAGAAGGAAAGCACATGTGCTGCGAACAATCTCATCATTAGCATTACATCAAAAGAGCTAGCTCAACGAATGGCTA : 1748
H18                     : GAAGCTACCGCCAATCGTTAAGAACAAAGAGAAGGAAAGCACATGTGCTGCGAACAATCTCATCATTAGCATTACATCAAAAGAGCTAGCTCAACGAATGGCTA : 1532
H19                     : GAAGCTACCGCCAATCGTTAAGAACAAAGAGAAGGAAAGCACATGTGCTGCGAACAATCTCATCATTAGCATTACATCAAAAGAGCTAGCTCAACGAATGGCTA : 1586
H20                     : GAAGCTACCGCCAATCGTTAAGAACAAAGAGAAGGAAAGCACATGTGCTGCGAACAATCTCATCATTAGCATTACATCAAAAGAGCTAGCTCAACGAATGGCTA : 1568
H21                     : GAAGCTACCGCCAATCGTTAAGAACAAAGAGAAGGAAAGCACATGTGCTGCGAACAATCTCATCATTAGCATTACATCAAAAGAGCTAGCTCAACGAATGGCTA : 1604
H22                     : GAAGCTACCGCCAATCGTTAAGAACAAAGAGAAGGAAAGCACATGTGCTGCGAACAATCTCATCATTAGCATTACATCAAAAGAGCTAGCTCAACGAATGGCTA : 1568
H23                     : GAAGCTACCGCCAATCGTTAAGAACAAAGAGAAGGAAAGCACATGTGCTGCGAACAATCTCATCATTAGCATTACATCAAAAGAGCTAGCTCAACGAATGGCTA : 1604
H24                     : GAAGCTACCGCCAATCGTTAAGAACAAAGAGAAGGAAAGCACATGTGCTGCGAACAATCTCATCATTAGCATTACATCAAAAGAGCTAGCTCAACGAATGGCTA : 1613
H25                     : GAAGCTACCGCCAATCGTTAAGAACAAAGAGAAGGAAAGCACATGTGCTGCGAACAATCTCATCATTAGCATTACATCAAAAGAGCTAGCTCAACGAATGGCTA : 1568
H26_Mauritania-I_ref    : GAAGCTACCGCCAATCGTTAAGAACAAAGAGAAGGAAAGCACATGTGCTGCGAACAATCTCATCATTAGCATTACATCAAAAGAGCTAGCTCAACGAATGGCTA : 1658
H27_India-VII_refseq    : GAAGCTACCGCCAATCGTTAAGAACAAAGAGAAGGAAAGCACATGTGCTGCGAACAATCTCATCATTAGCATTACATCAAAAGAGCTAGCTCAACGAATGGCTA : 1541
H28_Ctg_refsequence     : GAAGCTACCGCCAATCGTTAAGAACAAAGAGAAGGAAAGCACATGTGCTGCGAACAATCTCATCATTAGCATTACATCAAAAGAGCTAGCTCAACGAATGGCTA : 1613
H29_Salvador-I_refse    : GAAGCTACCGCCAATCGTTAAGAACAAAGAGAAGGAAAGCACATGTGCTGCGAACAATCTCATCATTAGCATTACATCAAAAGAGCTAGCTCAACGAATGGCTA : 1613
H30_NKorea_refsequen    : GAAGCTACCGCCAATCGTTAAGAACAAAGAGAAGGAAAGCACATGTGCTGCGAACAATCTCATCATTAGCATTACATCAAAAGAGCTAGCTCAACGAATGGCTA : 1523
H31_P.vivax01_refseq    : GAAGCTACCGCCAATCGTTAAGAACAAAGAGAAGGAAAGCACATGTGCTGCGAACAATCTCATCATTAGCATTACATCAAAAGAGCTAGCTCAACGAATGGCTA : 1604
H32_Brazil-I_refsequ    : GAAGCTACCGCCAATCGTTAAGAACAAAGAGAAGGAAAGCACATGTGCTGCGAACAATCTCATCATTAGCATTACATCAAAAGAGCTAGCTCAACGAATGGCTA : 1532
                          GAAGCTACCGCCAATCGTTAAGAACAAAGAGAAGGAAAGCACATGTGCTGCGAACAATCTCATCATTAGCATTACATCAAAAGAGCTAGCTCAACGAATGGCTA

                               1880         *       1900         *       1920         *       1940         *       1960         *
H1_VCG                  : TCATGTTCGCAAAATGGCTAGCTCCAGAAGAATACGGGTCTGTCGTCGATTTAGATAAAAGCATAGAATTAAATGTCCTCTGCGCAGGGGCACCTATCCTAGTA : 1636
H2                      : TCATGTTCGCAAAATGGCTAGCTCCAGAAGAATACGGGTCTGTCGTCGATTTAGATAAAAGCATAGAATTAAATGTCCTCTGCGCAGGGGCACCTATCCTAGTA : 1672
H3                      : TCATGTTCGCAAAATGGCTAGCTCCAGAAGAATACGGGTCTGTCGTCGATTTAGATAAAAGCATAGAATTAAATGTCCTCTGCGCAGGGGCACCTATCCTAGTA : 1744
H4                      : TCATGTTCGCAAAATGGCTAGCTCCAGAAGAATACGGGTCTGTCGTCGATTTAGATAAAAGCATAGAATTAAATGTCCTCTGCGCAGGGGCACCTATCCTAGTA : 1708
H5                      : TCATGTTCGCAAAATGGCTAGCTCCAGAAGAATACGGGTCTGTCGTCGATTTAGATAAAAGCATAGAATTAAATGTCCTCTGCGCAGGGGCACCTATCCTAGTA : 1708
H6                      : TCATGTTCGCAAAATGGCTAGCTCCAGAAGAATACGGGTCTGTCGTCGATTTAGATAAAAGCATAGAATTAAATGTCCTCTGCGCAGGGGCACCTATCCTAGTA : 1627
H7                      : TCATGTTCGCAAAATGGCTAGCTCCAGAAGAATACGGGTCTGTCGTCGATTTAGATAAAAGCATAGAATTAAATGTCCTCTGCGCAGGGGCACCTATCCTAGTA : 1645
H8                      : TCATGTTCGCAAAATGGCTAGCTCCAGAAGAATACGGGTCTGTCGTCGATTTAGATAAAAGCATAGAATTAAATGTCCTCTGCGCAGGGGCACCTATCCTAGTA : 1681
H9                      : TCATGTTCGCAAAATGGCTAGCTCCAGAAGAATACGGGTCTGTCGTCGATTTAGATAAAAGCATAGAATTAAATGTCCTCTGCGCAGGGGCACCTATCCTAGTA : 1763
H10                     : TCATGTTCGCAAAATGGCTAGCTCCAGAAGAATACGGGTCTGTCGTCGATTTAGATAAAAGCATAGAATTAAATGTCCTCTGCGCAGGGGCACCTATCCTAGTA : 1681
H11                     : TCATGTTCGCAAAATGGCTAGCTCCAGAAGAATACGGGTCTGTCGTCGATTTAGATAAAAGCATAGAATTAAATGTCCTCTGCGCAGGGGCACCTATCCTAGTA : 1690
H12                     : TCATGTTCGCAAAATGGCTAGCTCCAGAAGAATACGGGTCTGTCGTCGATTTAGATAAAAGCATAGAATTAAATGTCCTCTGCGCAGGGGCACCTATCCTAGTA : 1672
```

```
H13                   : TCATGTTCGCAAAATGGCTAGCTCCAGAAGAATACGGGTCTGTCGTCGATTTAGATAAAAGCATAGAATTAAATGTCCTCTGCGCAGGGGCACCTATCCTAGTA : 1744
H14                   : TCATGTTCGCAAAATGGCTAGCTCCAGAAGAATACGGGTCTGTCGTCGATTTAGATAAAAGCATAGAATTAAATGTCCTCTGCGCAGGGGCACCTATCCTAGTA : 1690
H15                   : TCATGTTCGCAAAATGGCTAGCTCCAGAAGAATACGGGTCTGTCGTCGATTTAGATAAAAGCATAGAATTAAATGTCCTCTGCGCAGGGGCACCTATCCTAGTA : 1672
H16                   : TCATGTTCGCAAAATGGCTAGCTCCAGAAGAATACGGGTCTGTCGTCGATTTAGATAAAAGCATAGAATTAAATGTCCTCTGCGCAGGGGCACCTATCCTAGTA : 1636
H17                   : TCATGTTCGCAAAATGGCTAGCTCCAGAAGAATACGGGTCTGTCGTCGATTTAGATAAAAGCATAGAATTAAATGTCCTCTGCGCAGGGGCACCTATCCTAGTA : 1852
H18                   : TCATGTTCGCAAAATGGCTAGCTCCAGAAGAATACGGGTCTGTCGTCGATTTAGATAAAAGCATAGAATTAAATGTCCTCTGCGCAGGGGCACCTATCCTAGTA : 1636
H19                   : TCATGTTCGCAAAATGGCTAGCTCCAGAAGAATACGGGTCTGTCGTCGATTTAGATAAAAGCATAGAATTAAATGTCCTCTGCGCAGGGGCACCTATCCTAGTA : 1690
H20                   : TCATGTTCGCAAAATGGCTAGCTCCAGAAGAATACGGGTCTGTCGTCGATTTAGATAAAAGCATAGAATTAAATGTCCTCTGCGCAGGGGCACCTATCCTAGTA : 1672
H21                   : TCATGTTCGCAAAATGGCTAGCTCCAGAAGAATACGGGTCTGTCGTCGATTTAGATAAAAGCATAGAATTAAATGTCCTCTGCGCAGGGGCACCTATCCTAGTA : 1708
H22                   : TCATGTTCGCAAAATGGCTAGCTCCAGAAGAATACGGGTCTGTCGTCGATTTAGATAAAAGCATAGAATTAAATGTCCTCTGCGCAGGGGCACCTATCCTAGTA : 1672
H23                   : TCATGTTCGCAAAATGGCTAGCTCCAGAAGAATACGGGTCTGTCGTCGATTTAGATAAAAGCATAGAATTAAATGTCCTCTGCGCAGGGGCACCTATCCTAGTA : 1708
H24                   : TCATGTTCGCAAAATGGCTAGCTCCAGAAGAATACGGGTCTGTCGTCGATTTAGATAAAAGCATAGAATTAAATGTCCTCTGCGCAGGGGCACCTATCCTAGTA : 1717
H25                   : TCATGTTCGCAAAATGGCTAGCTCCAGAAGAATACGGGTCTGTCGTCGATTTAGATAAAAGCATAGAATTAAATGTCCTCTGCGCAGGGGCACCTATCCTAGTA : 1672
H26_Mauritania-I_ref  : TCATGTTCGCAAAATGGCTAGCTCCAGAAGAATACGGGTCTGTCGTCGATTTAGATAAAAGCATAGAATTAAATGTCCTCTGCGCAGGGGCACCTATCCTAGTA : 1762
H27_India-VII_refseq  : TCATGTTCGCAAAATGGCTAGCTCCAGAAGAATACGGGTCTGTCGTCGATTTAGATAAAAGCATAGAATTAAATGTCCTCTGCGCAGGGGCACCTATCCTAGTA : 1645
H28_Ctg_refsequence   : TCATGTTCGCAAAATGGCTAGCTCCAGAAGAATACGGGTCTGTCGTCGATTTAGATAAAAGCATAGAATTAAATGTCCTCTGCGCAGGGGCACCTATCCTAGTA : 1717
H29_Salvador-I_refse  : TCATGTTCGCAAAATGGCTAGCTCCAGAAGAATACGGGTCTGTCGTCGATTTAGATAAAAGCATAGAATTAAATGTCCTCTGCGCAGGGGCACCTATCCTAGTA : 1717
H30_NKorea_refsequen  : TCATGTTCGCAAAATGGCTAGCTCCAGAAGAATACGGGTCTGTCGTCGATTTAGATAAAAGCATAGAATTAAATGTCCTCTGCGCAGGGGCACCTATCCTAGTA : 1627
H31_P.vivax01_refseq  : TCATGTTCGCAAAATGGCTAGCTCCAGAAGAATACGGGTCTGTCGTCGATTTAGATAAAAGCATAGAATTAAATGTCCTCTGCGCAGGGGCACCTATCCTAGTA : 1708
H32_Brazil-I_refsequ  : TCATGTTCGCAAAATGGCTAGCTCCAGAAGAATACGGGTCTGTCGTCGATTTAGATAAAAGCATAGAATTAAATGTCCTCTGCGCAGGGGCACCTATCCTAGTA : 1636
                        TCATGTTCGCAAAATGGCTAGCTCCAGAAGAATACGGGTCTGTCGTCGATTTAGATAAAAGCATAGAATTAAATGTCCTCTGCGCAGGGGCACCTATCCTAGTA

                        1980        *       2000        *       2020        *       2040        *       2060        *       2080
H1_VCG                : CAGCAATGGAAATATTACCAAAACATGTTAGGGTTCGAAACGGGAAATGAACATGCCTTCTTAAACCTCATCGATGAGTTGCTAGTCATCGACAAGAGACACAG : 1740
H2                    : CAGCAATGGAAATATTACCAAAACATGTTAGGGTTCGAAACGGGAAATGAACATGCCTTCTTAAACCTCATCGATGAGTTGCTAGTCATCGACAAGAGACACAG : 1776
H3                    : CAGCAATGGAAATATTACCAAAACATGTTAGGGTTCGAAACGGGAAATGAACATGCCTTCTTAAACCTCATCGATGAGTTGCTAGTCATCGACAAGAGACACAG : 1848
H4                    : CAGCAATGGAAATATTACCAAAACATGTTAGGGTTCGAAACGGGAAATGAACATGCCTTCTTAAACCTCATCGATGAGTTGCTAGTCATCGACAAGAGACACAG : 1812
H5                    : CAGCAATGGAAATATTACCAAAACATGTTAGGGTTCGAAACGGGAAATGAACATGCCTTCTTAAACCTCATCGATGAGTTGCTAGTCATCGACAAGAGACACAG : 1812
H6                    : CAGCAATGGAAATATTACCAAAACATGTTAGGGTTCGAAACGGGAAATGAACATGCCTTCTTAAACCTCATCGATGAGTTGCTAGTCATCGACAAGAGACACAG : 1731
H7                    : CAGCAATGGAAATATTACCAAAACATGTTAGGGTTCGAAACGGGAAATGAACATGCCTTCTTAAACCTCATCGATGAGTTGCTAGTCATCGACAAGAGACACAG : 1749
H8                    : CAGCAATGGAAATATTACCAAAACATGTTAGGGTTCGAAACGGGAAATGAACATGCCTTCTTAAACCTCATCGATGAGTTGCTAGTCATCGACAAGAGACACAG : 1785
H9                    : CAGCAATGGAAATATTACCAAAACATGTTAGGGTTCGAAACGGGAAATGAACATGCCTTCTTAAACCTCATCGATGAGTTGCTAGTCATCGACAAGAGACACAG : 1867
H10                   : CAGCAATGGAAATATTACCAAAACATGTTAGGGTTCGAAACGGGAAATGAACATGCCTTCTTAAACCTCATCGATGAGTTGCTAGTCATCGACAAGAGACACAG : 1785
H11                   : CAGCAATGGAAATATTACCAAAACATGTTAGGGTTCGAAACGGGAAATGAACATGCCTTCTTAAACCTCATCGATGAGTTGCTAGTCATCGACAAGAGACACAG : 1794
H12                   : CAGCAATGGAAATATTACCAAAACATGTTAGGGTTCGAAACGGGAAATGAACATGCCTTCTTAAACCTCATCGATGAGTTGCTAGTCATCGACAAGAGACACAG : 1776
H13                   : CAGCAATGGAAATATTACCAAAACATGTTAGGGTTCGAAACGGGAAATGAACATGCCTTCTTAAACCTCATCGATGAGTTGCTAGTCATCGACAAGAGACACAG : 1848
H14                   : CAGCAATGGAAATATTACCAAAACATGTTAGGGTTCGAAACGGGAAATGAACATGCCTTCTTAAACCTCATCGATGAGTTGCTAGTCATCGACAAGAGACACAG : 1794
H15                   : CAGCAATGGAAATATTACCAAAACATGTTAGGGTTCGAAACGGGAAATGAACATGCCTTCTTAAACCTCATCGATGAGTTGCTAGTCATCGACAAGAGACACAG : 1776
H16                   : CAGCAATGGAAATATTACCAAAACATGTTAGGGTTCGAAACGGGAAATGAACATGCCTTCTTAAACCTCATCGATGAGTTGCTAGTCATCGACAAGAGACACAG : 1740
H17                   : CAGCAATGGAAATATTACCAAAACATGTTAGGGTTCGAAACGGGAAATGAACATGCCTTCTTAAACCTCATCGATGAGTTGCTAGTCATCGACAAGAGACACAG : 1956
H18                   : CAGCAATGGAAATATTACCAAAACATGTTAGGGTTCGAAACGGGAAATGAACATGCCTTCTTAAACCTCATCGATGAGTTGCTAGTCATCGACAAGAGACACAG : 1740
H19                   : CAGCAATGGAAATATTACCAAAACATGTTAGGGTTCGAAACGGGAAATGAACATGCCTTCTTAAACCTCATCGATGAGTTGCTAGTCATCGACAAGAGACACAG : 1794
H20                   : CAGCAATGGAAATATTACCAAAACATGTTAGGGTTCGAAACGGGAAATGAACATGCCTTCTTAAACCTCATCGATGAGTTGCTAGTCATCGACAAGAGACACAG : 1776
H21                   : CAGCAATGGAAATATTACCAAAACATGTTAGGGTTCGAAACGGGAAATGAACATGCCTTCTTAAACCTCATCGATGAGTTGCTAGTCATCGACAAGAGACACAG : 1812
H22                   : CAGCAATGGAAATATTACCAAAACATGTTAGGGTTCGAAACGGGAAATGAACATGCCTTCTTAAACCTCATCGATGAGTTGCTAGTCATCGACAAGAGACACAG : 1776
H23                   : CAGCAATGGAAATATTACCAAAACATGTTAGGGTTCGAAACGGGAAATGAACATGCCTTCTTAAACCTCATCGATGAGTTGCTAGTCATCGACAAGAGACACAG : 1812
H24                   : CAGCAATGGAAATATTACCAAAACATGTTAGGGTTCGAAACGGGAAATGAACATGCCTTCTTAAACCTCATCGATGAGTTGCTAGTCATCGACAAGAGACACAG : 1821
H25                   : CAGCAATGGAAATATTACCAAAACATGTTAGGGTTCGAAACGGGAAATGAACATGCCTTCTTAAACCTCATCGATGAGTTGCTAGTCATCGACAAGAGACACAG : 1776
H26_Mauritania-I_ref  : CAGCAATGGAAATATTACCAAAACATGTTAGGGTTCGAAACGGGAAATGAACATGCCTTCTTAAACCTCATCGATGAGTTGCTAGTCATCGACAAGAGACACAG : 1866
H27_India-VII_refseq  : CAGCAATGGAAATATTACCAAAACATGTTAGGGTTCGAAACGGGAAATGAACATGCCTTCTTAAACCTCATCGATGAGTTGCTAGTCATCGACAAGAGACACAG : 1749
```

```
H28_Ctg_refsequence  : CAGCAATGGAAATATTACCAAAACATGTTAGGGTTCGAAACGGGAAATGAACATGCCTTCTTAAACCTCATCGATGAGTTGCTAGTCATCGACAAGAGACACAG : 1821
H29_Salvador-I_refse : CAGCAATGGAAATATTACCAAAACATGTTAGGGTTCGAAACGGGAAATGAACATGCCTTCTTAAACCTCATCGATGAGTTGCTAGTCATCGACAAGAGACACAG : 1821
H30_NKorea_refsequen : CAGCAATGGAAATATTACCAAAACATGTTAGGGTTCGAAACGGGAAATGAACATGCCTTCTTAAACCTCATCGATGAGTTGCTAGTCATCGACAAGAGACACAG : 1731
H31_P.vivax01_refseq : CAGCAATGGAAATATTACCAAAACATGTTAGGGTTCGAAACGGGAAATGAACATGCCTTCTTAAACCTCATCGATGAGTTGCTAGTCATCGACAAGAGACACAG : 1812
H32_Brazil-I_refsequ : CAGCAATGGAAATATTACCAAAACATGTTAGGGTTCGAAACGGGAAATGAACATGCCTTCTTAAACCTCATCGATGAGTTGCTAGTCATCGACAAGAGACACAG : 1740
                       CAGCAATGGAAATATTACCAAAACATGTTAGGGTTCGAAACGGGAAATGAACATGCCTTCTTAAACCTCATCGATGAGTTGCTAGTCATCGACAAGAGACACAG

                                *       2100         *       2120         *       2140         *       2160         *       2180
H1_VCG               : TAACAATGAAGCGTATTCAAAAGTGATAAGGAAAATAAAAAAATCGAAAGCCTTTAATTATTGTACTAAAGTTATGAGGATTGCAGGGAATATTTCTTCCATTC : 1844
H2                   : TAACAATGAAGCGTATTCAAAAGTGATAAGGAAAATAAAAAAATCGAAAGCCTTTAATTATTGTACTAAAGTTATGAGGATTGCAGGGAATATTTCTTCCATTC : 1880
H3                   : TAACAATGAAGCGTATTCAAAAGTGATAAGGAAAATAAAAAAATCGAAAGCCTTTAATTATTGTACTAAAGTTATGAGGATTGCAGGGAATATTTCTTCCATTC : 1952
H4                   : TAACAATGAAGCGTATTCAAAAGTGATAAGGAAAATAAAAAAATCGAAAGCCTTTAATTATTGTACTAAAGTTATGAGGATTGCAGGGAATATTTCTTCCATTC : 1916
H5                   : TAACAATGAAGCGTATTCAAAAGTGATAAGGAAAATAAAAAAATCGAAAGCCTTTAATTATTGTACTAAAGTTATGAGGATTGCAGGGAATATTTCTTCCATTC : 1916
H6                   : TAACAATGAAGCGTATTCAAAAGTGATAAGGAAAATAAAAAAATCGAAAGCCTTTAATTATTGTACTAAAGTTATGAGGATTGCAGGGAATATTTCTTCCATTC : 1835
H7                   : TAACAATGAAGCGTATTCAAAAGTGATAAGGAAAATAAAAAAATCGAAAGCCTTTAATTATTGTACTAAAGTTATGAGGATTGCAGGGAATATTTCTTCCATTC : 1853
H8                   : TAACAATGAAGCGTATTCAAAAGTGATAAGGAAAATAAAAAAATCGAAAGCCTTTAATTATTGTACTAAAGTTATGAGGATTGCAGGGAATATTTCTTCCATTC : 1889
H9                   : TAACAATGAAGCGTATTCAAAAGTGATAAGGAAAATAAAAAAATCGAAAGCCTTTAATTATTGTACTAAAGTTATGAGGATTGCAGGGAATATTTCTTCCATTC : 1971
H10                  : TAACAATGAAGCGTATTCAAAAGTGATAAGGAAAATAAAAAAATCGAAAGCCTTTAATTATTGTACTAAAGTTATGAGGATTGCAGGGAATATTTCTTCCATTC : 1889
H11                  : TAACAATGAAGCGTATTCAAAAGTGATAAGGAAAATAAAAAAATCGAAAGCCTTTAATTATTGTACTAAAGTTATGAGGATTGCAGGGAATATTTCTTCCATTC : 1898
H12                  : TAACAATGAAGCGTATTCAAAAGTGATAAGGAAAATAAAAAAATCGAAAGCCTTTAATTATTGTACTAAAGTTATGAGGATTGCAGGGAATATTTCTTCCATTC : 1880
H13                  : TAACAATGAAGCGTATTCAAAAGTGATAAGGAAAATAAAAAAATCGAAAGCCTTTAATTATTGTACTAAAGTTATGAGGATTGCAGGGAATATTTCTTCCATTC : 1952
H14                  : TAACAATGAAGCGTATTCAAAAGTGATAAGGAAAATAAAAAAATCGAAAGCCTTTAATTATTGTACTAAAGTTATGAGGATTGCAGGGAATATTTCTTCCATTC : 1898
H15                  : TAACAATGAAGCGTATTCAAAAGTGATAAGGAAAATAAAAAAATCGAAAGCCTTTAATTATTGTACTAAAGTTATGAGGATTGCAGGGAATATTTCTTCCATTC : 1880
H16                  : TAACAATGAAGCGTATTCAAAAGTGATAAGGAAAATAAAAAAATCGAAAGCCTTTAATTATTGTACTAAAGTTATGAGGATTGCAGGGAATATTTCTTCCATTC : 1844
H17                  : TAACAATGAAGCGTATTCAAAAGTGATAAGGAAAATAAAAAAATCGAAAGCCTTTAATTATTGTACTAAAGTTATGAGGATTGCAGGGAATATTTCTTCCATTC : 2060
H18                  : TAACAATGAAGCGTATTCAAAAGTGATAAGGAAAATAAAAAAATCGAAAGCCTTTAATTATTGTACTAAAGTTATGAGGATTGCAGGGAATATTTCTTCCATTC : 1844
H19                  : TAACAATGAAGCGTATTCAAAAGTGATAAGGAAAATAAAAAAATCGAAAGCCTTTAATTATTGTACTAAAGTTATGAGGATTGCAGGGAATATTTCTTCCATTC : 1898
H20                  : TAACAATGAAGCGTATTCAAAAGTGATAAGGAAAATAAAAAAATCGAAAGCCTTTAATTATTGTACTAAAGTTATGAGGATTGCAGGGAATATTTCTTCCATTC : 1880
H21                  : TAACAATGAAGCGTATTCAAAAGTGATAAGGAAAATAAAAAAATCGAAAGCCTTTAATTATTGTACTAAAGTTATGAGGATTGCAGGGAATATTTCTTCCATTC : 1916
H22                  : TAACAATGAAGCGTATTCAAAAGTGATAAGGAAAATAAAAAAATCGAAAGCCTTTAATTATTGTACTAAAGTTATGAGGATTGCAGGGAATATTTCTTCCATTC : 1880
H23                  : TAACAATGAAGCGTATTCAAAAGTGATAAGGAAAATAAAAAAATCGAAAGCCTTTAATTATTGTACTAAAGTTATGAGGATTGCAGGGAATATTTCTTCCATTC : 1916
H24                  : TAACAATGAAGCGTATTCAAAAGTGATAAGGAAAATAAAAAAATCGAAAGCCTTTAATTATTGTACTAAAGTTATGAGGATTGCAGGGAATATTTCTTCCATTC : 1925
H25                  : TAACAATGAAGCGTATTCAAAAGTGATAAGGAAAATAAAAAAATCGAAAGCCTTTAATTATTGTACTAAAGTTATGAGGATTGCAGGGAATATTTCTTCCATTC : 1880
H26_Mauritania-I_ref : TAACAATGAAGCGTATTCAAAAGTGATAAGGAAAATAAAAAAATCGAAAGCCTTTAATTATTGTACTAAAGTTATGAGGATTGCAGGGAATATTTCTTCCATTC : 1970
H27_India-VII_refseq : TAACAATGAAGCGTATTCAAAAGTGATAAGGAAAATAAAAAAATCGAAAGCCTTTAATTATTGTACTAAAGTTATGAGGATTGCAGGGAATATTTCTTCCATTC : 1853
H28_Ctg_refsequence  : TAACAATGAAGCGTATTCAAAAGTGATAAGGAAAATAAAAAAATCGAAAGCCTTTAATTATTGTACTAAAGTTATGAGGATTGCAGGGAATATTTCTTCCATTC : 1925
H29_Salvador-I_refse : TAACAATGAAGCGTATTCAAAAGTGATAAGGAAAATAAAAAAATCGAAAGCCTTTAATTATTGTACTAAAGTTATGAGGATTGCAGGGAATATTTCTTCCATTC : 1925
H30_NKorea_refsequen : TAACAATGAAGCGTATTCAAAAGTGATAAGGAAAATAAAAAAATCGAAAGCCTTTAATTATTGTACTAAAGTTATGAGGATTGCAGGGAATATTTCTTCCATTC : 1835
H31_P.vivax01_refseq : TAACAATGAAGCGTATTCAAAAGTGATAAGGAAAATAAAAAAATCGAAAGCCTTTAATTATTGTACTAAAGTTATGAGGATTGCAGGGAATATTTCTTCCATTC : 1916
H32_Brazil-I_refsequ : TAACAATGAAGCGTATTCAAAAGTGATAAGGAAAATAAAAAAATCGAAAGCCTTTAATTATTGTACTAAAGTTATGAGGATTGCAGGGAATATTTCTTCCATTC : 1844
                       TAACAATGAAGCGTATTCAAAAGTGATAAGGAAAATAAAAAAATCGAAAGCCTTTAATTATTGTACTAAAGTTATGAGGATTGCAGGGAATATTTCTTCCATTC

                            *       2200         *       2220         *       2240         *       2260         *       2280
H1_VCG               : CATTTAATCATGAAAATAATAAAACGCCTAGCTACTCCATCATCGGGTCGTTAGGTAACCTGGTGAAGGCTCACATGGGGAACTACTACGTTGCCATTGCTAAT : 1948
H2                   : CATTTAATCATGAAAATAATAAAACGCCTAGCTACTCCATCATCGGGTCGTTAGGTAACCTGGTGAAGGCTCACATGGGGAACTACTACGTTGCCATTGCTAAT : 1984
H3                   : CATTTAATCATGAAAATAATAAAACGCCTAGCTACTCCATCATCGGGTCGTTAGGTAACCTGGTGAAGGCTCACATGGGGAACTACTACGTTGCCATTGCTAAT : 2056
H4                   : CATTTAATCATGAAAATAATAAAACGCCTAGCTACTCCATCATCGGGTCGTTAGGTAACCTGGTGAAGGCTCACATGGGGAACTACTACGTTGCCATTGCTAAT : 2020
H5                   : CATTTAATCATGAAAATAATAAAACGCCTAGCTACTCCATCATCGGGTCGTTAGGTAACCTGGTGAAGGCTCACATGGGGAACTACTACGTTGCCATTGCTAAT : 2020
H6                   : CATTTAATCATGAAAATAATAAAACGCCTAGCTACTCCATCATCGGGTCGTTAGGTAACCTGGTGAAGGCTCACATGGGGAACTACTACGTTGCCATTGCTAAT : 1939
H7                   : CATTTAATCATGAAAATAATAAAACGCCTAGCTACTCCATCATCGGGTCGTTAGGTAACCTGGTGAAGGCTCACATGGGGAACTACTACGTTGCCATTGCTAAT : 1957
```

```
H8                      : CATTTAATCATGAAAATAATAAAACGCCTAGCTACTCCATCATCGGGTCGTTAGGTAACCTGGTGAAGGCTCACATGGGGAACTACTACGTTGCCATTGCTAAT : 1993
H9                      : CATTTAATCATGAAAATAATAAAACGCCTAGCTACTCCATCATCGGGTCGTTAGGTAACCTGGTGAAGGCTCACATGGGGAACTACTACGTTGCCATTGCTAAT : 2075
H10                     : CATTTAATCATGAAAATAATAAAACGCCTAGCTACTCCATCATCGGGTCGTTAGGTAACCTGGTGAAGGCTCACATGGGGAACTACTACGTTGCCATTGCTAAT : 1993
H11                     : CATTTAATCATGAAAATAATAAAACGCCTAGCTACTCCATCATCGGGTCGTTAGGTAACCTGGTGAAGGCTCACATGGGGAACTACTACGTTGCCATTGCTAAT : 2002
H12                     : CATTTAATCATGAAAATAATAAAACGCCTAGCTACTCCATCATCGGGTCGTTAGGTAACCTGGTGAAGGCTCACATGGGGAACTACTACGTTGCCATTGCTAAT : 1984
H13                     : CATTTAATCATGAAAATAATAAAACGCCTAGCTACTCCATCATCGGGTCGTTAGGTAACCTGGTGAAGGCTCACATGGGGAACTACTACGTTGCCATTGCTAAT : 2056
H14                     : CATTTAATCATGAAAATAATAAAACGCCTAGCTACTCCATCATCGGGTCGTTAGGTAACCTGGTGAAGGCTCACATGGGGAACTACTACGTTGCCATTGCTAAT : 2002
H15                     : CATTTAATCATGAAAATAATAAAACGCCTAGCTACTCCATCATCGGGTCGTTAGGTAACCTGGTGAAGGCTCACATGGGGAACTACTACGTTGCCATTGCTAAT : 1984
H16                     : CATTTAATCATGAAAATAATAAAACGCCTAGCTACTCCATCATCGGGTCGTTAGGTAACCTGGTGAAGGCTCACATGGGGAACTACTACGTTGCCATTGCTAAT : 1948
H17                     : CATTTAATCATGAAAATAATAAAACGCCTAGCTACTCCATCATCGGGTCGTTAGGTAACCTGGTGAAGGCTCACATGGGGAACTACTACGTTGCCATTGCTAAT : 2164
H18                     : CATTTAATCATGAAAATAATAAAACGCCTAGCTACTCCATCATCGGGTCGTTAGGTAACCTGGTGAAGGCTCACATGGGGAACTACTACGTTGCCATTGCTAAT : 1948
H19                     : CATTTAATCATGAAAATAATAAAACGCCTAGCTACTCCATCATCGGGTCGTTAGGTAACCTGGTGAAGGCTCACATGGGGAACTACTACGTTGCCATTGCTAAT : 2002
H20                     : CATTTAATCATGAAAATAATAAAACGCCTAGCTACTCCATCATCGGGTCGTTAGGTAACCTGGTGAAGGCTCACATGGGGAACTACTACGTTGCCATTGCTAAT : 1984
H21                     : CATTTAATCATGAAAATAATAAAACGCCTAGCTACTCCATCATCGGGTCGTTAGGTAACCTGGTGAAGGCTCACATGGGGAACTACTACGTTGCCATTGCTAAT : 2020
H22                     : CATTTAATCATGAAAATAATAAAACGCCTAGCTACTCCATCATCGGGTCGTTAGGTAACCTGGTGAAGGCTCACATGGGGAACTACTACGTTGCCATTGCTAAT : 1984
H23                     : CATTTAATCATGAAAATAATAAAACGCCTAGCTACTCCATCATCGGGTCGTTAGGTAACCTGGTGAAGGCTCACATGGGGAACTACTACGTTGCCATTGCTAAT : 2020
H24                     : CATTTAATCATGAAAATAATAAAACGCCTAGCTACTCCATCATCGGGTCGTTAGGTAACCTGGTGAAGGCTCACATGGGGAACTACTACGTTGCCATTGCTAAT : 2029
H25                     : CATTTAATCATGAAAATAATAAAACGCCTAGCTACTCCATCATCGGGTCGTTAGGTAACCTGGTGAAGGCTCACATGGGGAACTACTACGTTGCCATTGCTAAT : 1984
H26_Mauritania-I_ref    : CATTTAATCATGAAAATAATAAAACGCCTAGCTACTCCATCATCGGGTCGTTAGGTAACCTGGTGAAGGCTCACATGGGGAACTACTACGTTGCCATTGCTAAT : 2074
H27_India-VII_refseq    : CATTTAATCATGAAAATAATAAAACGCCTAGCTACTCCATCATCGGGTCGTTAGGTAACCTGGTGAAGGCTCACATGGGGAACTACTACGTTGCCATTGCTAAT : 1957
H28_Ctg_refsequence     : CATTTAATCATGAAAATAATAAAACGCCTAGCTACTCCATCATCGGGTCGTTAGGTAACCTGGTGAAGGCTCACATGGGGAACTACTACGTTGCCATTGCTAAT : 2029
H29_Salvador-I_refse    : CATTTAATCATGAAAATAATAAAACGCCTAGCTACTCCATCATCGGGTCGTTAGGTAACCTGGTGAAGGCTCACATGGGGAACTACTACGTTGCCATTGCTAAT : 2029
H30_NKorea_refsequen    : CATTTAATCATGAAAATAATAAAACGCCTAGCTACTCCATCATCGGGTCGTTAGGTAACCTGGTGAAGGCTCACATGGGGAACTACTACGTTGCCATTGCTAAT : 1939
H31_P.vivax01_refseq    : CATTTAATCATGAAAATAATAAAACGCCTAGCTACTCCATCATCGGGTCGTTAGGTAACCTGGTGAAGGCTCACATGGGGAACTACTACGTTGCCATTGCTAAT : 2020
H32_Brazil-I_refsequ    : CATTTAATCATGAAAATAATAAAACGCCTAGCTACTCCATCATCGGGTCGTTAGGTAACCTGGTGAAGGCTCACATGGGGAACTACTACGTTGCCATTGCTAAT : 1948
                          CATTTAATCATGAAAATAATAAAACGCCTAGCTACTCCATCATCGGGTCGTTAGGTAACCTGGTGAAGGCTCACATGGGGAACTACTACGTTGCCATTGCTAAT

                            *      2300        *      2320        *      2340        *      2360        *      2380        *
H1_VCG                  : AGGATTAACTCCTATTTTGCCTATGCCGAGAAGAGGAACAAAAAAAACAGCCCCTTAAAAGTTGTCTCCGTCTGTACTCTCCTTCATTTGACCGACATGCTCCA : 2052
H2                      : AGGATTAACTCCTATTTTGCCTATGCCGAGAAGAGGAACAAAAAAAACAGCCCCTTAAAAGTTGTCTCCGTCTGTACTCTCCTTCATTTGACCGACATGCTCCA : 2088
H3                      : AGGATTAACTCCTATTTTGCCTATGCCGAGAAGAGGAACAAAAAAAACAGCCCCTTAAAAGTTGTCTCCGTCTGTACTCTCCTTCATTTGACCGACATGCTCCA : 2160
H4                      : AGGATTAACTCCTATTTTGCCTATGCCGAGAAGAGGAACAAAAAAAACAGCCCCTTAAAAGTTGTCTCCGTCTGTACTCTCCTTCATTTGACCGACATGCTCCA : 2124
H5                      : AGGATTAACTCCTATTTTGCCTATGCCGAGAAGAGGAACAAAAAAAACAGCCCCTTAAAAGTTGTCTCCGTCTGTACTCTCCTTCATTTGACCGACATGCTCCA : 2124
H6                      : AGGATTAACTCCTATTTTGCCTATGCCGAGAAGAGGAACAAAAAAAACAGCCCCTTAAAAGTTGTCTCCGTCTGTACTCTCCTTCATTTGACCGACATGCTCCA : 2043
H7                      : AGGATTAACTCCTATTTTGCCTATGCCGAGAAGAGGAACAAAAAAAACAGCCCCTTAAAAGTTGTCTCCGTCTGTACTCTCCTTCATTTGACCGACATGCTCCA : 2061
H8                      : AGGATTAACTCCTATTTTGCCTATGCCGAGAAGAGGAACAAAAAAAACAGCCCCTTAAAAGTTGTCTCCGTCTGTACTCTCCTTCATTTGACCGACATGCTCCA : 2097
H9                      : AGGATTAACTCCTATTTTGCCTATGCCGAGAAGAGGAACAAAAAAAACAGCCCCTTAAAAGTTGTCTCCGTCTGTACTCTCCTTCATTTGACCGACATGCTCCA : 2179
H10                     : AGGATTAACTCCTATTTTGCCTATGCCGAGAAGAGGAACAAAAAAAACAGCCCCTTAAAAGTTGTCTCCGTCTGTACTCTCCTTCATTTGACCGACATGCTCCA : 2097
H11                     : AGGATTAACTCCTATTTTGCCTATGCCGAGAAGAGGAACAAAAAAAACAGCCCCTTAAAAGTTGTCTCCGTCTGTACTCTCCTTCATTTGACCGACATGCTCCA : 2106
H12                     : AGGATTAACTCCTATTTTGCCTATGCCGAGAAGAGGAACAAAAAAAACAGCCCCTTAAAAGTTGTCTCCGTCTGTACTCTCCTTCATTTGACCGACATGCTCCA : 2088
H13                     : AGGATTAACTCCTATTTTGCCTATGCCGAGAAGAGGAACAAAAAAAACAGCCCCTTAAAAGTTGTCTCCGTCTGTACTCTCCTTCATTTGACCGACATGCTCCA : 2160
H14                     : AGGATTAACTCCTATTTTGCCTATGCCGAGAAGAGGAACAAAAAAAACAGCCCCTTAAAAGTTGTCTCCGTCTGTACTCTCCTTCATTTGACCGACATGCTCCA : 2106
H15                     : AGGATTAACTCCTATTTTGCCTATGCCGAGAAGAGGAACAAAAAAAACAGCCCCTTAAAAGTTGTCTCCGTCTGTACTCTCCTTCATTTGACCGACATGCTCCA : 2088
H16                     : AGGATTAACTCCTATTTTGCCTATGCCGAGAAGAGGAACAAAAAAAACAGCCCCTTAAAAGTTGTCTCCGTCTGTACTCTCCTTCATTTGACCGACATGCTCCA : 2052
H17                     : AGGATTAACTCCTATTTTGCCTATGCCGAGAAGAGGAACAAAAAAAACAGCCCCTTAAAAGTTGTCTCCGTCTGTACTCTCCTTCATTTGACCGACATGCTCCA : 2268
H18                     : AGGATTAACTCCTATTTTGCCTATGCCGAGAAGAGGAACAAAAAAAACAGCCCCTTAAAAGTTGTCTCCGTCTGTACTCTCCTTCATTTGACCGACATGCTCCA : 2052
H19                     : AGGATTAACTCCTATTTTGCCTATGCCGAGAAGAGGAACAAAAAAAACAGCCCCTTAAAAGTTGTCTCCGTCTGTACTCTCCTTCATTTGACCGACATGCTCCA : 2106
H20                     : AGGATTAACTCCTATTTTGCCTATGCCGAGAAGAGGAACAAAAAAAACAGCCCCTTAAAAGTTGTCTCCGTCTGTACTCTCCTTCATTTGACCGACATGCTCCA : 2088
H21                     : AGGATTAACTCCTATTTTGCCTATGCCGAGAAGAGGAACAAAAAAAACAGCCCCTTAAAAGTTGTCTCCGTCTGTACTCTCCTTCATTTGACCGACATGCTCCA : 2124
H22                     : AGGATTAACTCCTATTTTGCCTATGCCGAGAAGAGGAACAAAAAAAACAGCCCCTTAAAAGTTGTCTCCGTCTGTACTCTCCTTCATTTGACCGACATGCTCCA : 2088
```

```
H23                    : AGGATTAACTCCTATTTTGCCTATGCCGAGAAGAGGAACAAAAAAAACAGCCCCTTAAAAGTTGTCTCCGTCTGTACTCTCCTTCATTTGACCGACATGCTCCA : 2124
H24                    : AGGATTAACTCCTATTTTGCCTATGCCGAGAAGAGGAACAAAAAAAACAGCCCCTTAAAAGTTGTCTCCGTCTGTACTCTCCTTCATTTGACCGACATGCTCCA : 2133
H25                    : AGGATTAACTCCTATTTTGCCTATGCCGAGAAGAGGAACAAAAAAAACAGCCCCTTAAAAGTTGTCTCCGTCTGTACTCTCCTTCATTTGACCGACATGCTCCA : 2088
H26_Mauritania-I_ref   : AGGATTAACTCCTATTTTGCCTATGCCGAGAAGAGGAACAAAAAAAACAGCCCCTTAAAAGTTGTCTCCGTCTGTACTCTCCTTCATTTGACCGACATGCTCCA : 2178
H27_India-VII_refseq   : AGGATTAACTCCTATTTTGCCTATGCCGAGAAGAGGAACAAAAAAAACAGCCCCTTAAAAGTTGTCTCCGTCTGTACTCTCCTTCATTTGACCGACATGCTCCA : 2061
H28_Ctg_refsequence    : AGGATTAACTCCTATTTTGCCTATGCCGAGAAGAGGAACAAAAAAAACAGCCCCTTAAAAGTTGTCTCCGTCTGTACTCTCCTTCATTTGACCGACATGCTCCA : 2133
H29_Salvador-I_refse   : AGGATTAACTCCTATTTTGCCTATGCCGAGAAGAGGAACAAAAAAAACAGCCCCTTAAAAGTTGTCTCCGTCTGTACTCTCCTTCATTTGACCGACATGCTCCA : 2133
H30_NKorea_refsequen   : AGGATTAACTCCTATTTTGCCTATGCCGAGAAGAGGAACAAAAAAAACAGCCCCTTAAAAGTTGTCTCCGTCTGTACTCTCCTTCATTTGACCGACATGCTCCA : 2043
H31_P.vivax01_refseq   : AGGATTAACTCCTATTTTGCCTATGCCGAGAAGAGGAACAAAAAAAACAGCCCCTTAAAAGTTGTCTCCGTCTGTACTCTCCTTCATTTGACCGACATGCTCCA : 2124
H32_Brazil-I_refsequ   : AGGATTAACTCCTATTTTGCCTATGCCGAGAAGAGGAACAAAAAAAACAGCCCCTTAAAAGTTGTCTCCGTCTGTACTCTCCTTCATTTGACCGACATGCTCCA : 2052
                         AGGATTAACTCCTATTTTGCCTATGCCGAGAAGAGGAACAAAAAAAACAGCCCCTTAAAAGTTGTCTCCGTCTGTACTCTCCTTCATTTGACCGACATGCTCCA

                             2400         *       2420         *       2440         *       2460         *       2480         *
H1_VCG                 : CAATTGCTCTGATGAACATTTAAAGAATATTTTGGACCTCAACACTTTGAAGCTTAACATTCTGAACATGCAGGGCAGGCGCGTGCTGCAGCCCCTCGTCAAGA : 2156
H2                     : CAATTGCTCTGATGAACATTTAAAGAATATTTTGGACCTCAACACTTTGAAGCTTAACATTCTGAACATGCAGGGCAGGCGCGTGCTGCAGCCCCTCGTCAAGA : 2192
H3                     : CAATTGCTCTGATGAACATTTAAAGAATATTTTGGACCTCAACACTTTGAAGCTTAACATTCTGAACATGCAGGGCAGGCGCGTGCTGCAGCCCCTCGTCAAGA : 2264
H4                     : CAATTGCTCTGATGAACATTTAAAGAATATTTTGGACCTCAACACTTTGAAGCTTAACATTCTGAACATGCAGGGCAGGCGCGTGCTGCAGCCCCTCGTCAAGA : 2228
H5                     : CAATTGCTCTGATGAACATTTAAAGAATATTTTGGACCTCAACACTTTGAAGCTTAACATTCTGAACATGCAGGGCAGGCGCGTGCTGCAGCCCCTCGTCAAGA : 2228
H6                     : CAATTGCTCTGATGAACATTTAAAGAATATTTTGGACCTCAACACTTTGAAGCTTAACATTCTGAACATGCAGGGCAGGCGCGTGCTGCAGCCCCTCGTCAAGA : 2147
H7                     : CAATTGCTCTGATGAACATTTAAAGAATATTTTGGACCTCAACACTTTGAAGCTTAACATTCTGAACATGCAGGGCAGGCGCGTGCTGCAGCCCCTCGTCAAGA : 2165
H8                     : CAATTGCTCTGATGAACATTTAAAGAATATTTTGGACCTCAACACTTTGAAGCTTAACATTCTGAACATGCAGGGCAGGCGCGTGCTGCAGCCCCTCGTCAAGA : 2201
H9                     : CAATTGCTCTGATGAACATTTAAAGAATATTTTGGACCTCAACACTTTGAAGCTTAACATTCTGAACATGCAGGGCAGGCGCGTGCTGCAGCCCCTCGTCAAGA : 2283
H10                    : CAATTGCTCTGATGAACATTTAAAGAATATTTTGGACCTCAACACTTTGAAGCTTAACATTCTGAACATGCAGGGCAGGCGCGTGCTGCAGCCCCTCGTCAAGA : 2201
H11                    : CAATTGCTCTGATGAACATTTAAAGAATATTTTGGACCTCAACACTTTGAAGCTTAACATTCTGAACATGCAGGGCAGGCGCGTGCTGCAGCCCCTCGTCAAGA : 2210
H12                    : CAATTGCTCTGATGAACATTTAAAGAATATTTTGGACCTCAACACTTTGAAGCTTAACATTCTGAACATGCAGGGCAGGCGCGTGCTGCAGCCCCTCGTCAAGA : 2192
H13                    : CAATTGCTCTGATGAACATTTAAAGAATATTTTGGACCTCAACACTTTGAAGCTTAACATTCTGAACATGCAGGGCAGGCGCGTGCTGCAGCCCCTCGTCAAGA : 2264
H14                    : CAATTGCTCTGATGAACATTTAAAGAATATTTTGGACCTCAACACTTTGAAGCTTAACATTCTGAACATGCAGGGCAGGCGCGTGCTGCAGCCCCTCGTCAAGA : 2210
H15                    : CAATTGCTCTGATGAACATTTAAAGAATATTTTGGACCTCAACACTTTGAAGCTTAACATTCTGAACATGCAGGGCAGGCGCGTGCTGCAGCCCCTCGTCAAGA : 2192
H16                    : CAATTGCTCTGATGAACATTTAAAGAATATTTTGGACCTCAACACTTTGAAGCTTAACATTCTGAACATGCAGGGCAGGCGCGTGCTGCAGCCCCTCGTCAAGA : 2156
H17                    : CAATTGCTCTGATGAACATTTAAAGAATATTTTGGACCTCAACACTTTGAAGCTTAACATTCTGAACATGCAGGGCAGGCGCGTGCTGCAGCCCCTCGTCAAGA : 2372
H18                    : CAATTGCTCTGATGAACATTTAAAGAATATTTTGGACCTCAACACTTTGAAGCTTAACATTCTGAACATGCAGGGCAGGCGCGTGCTGCAGCCCCTCGTCAAGA : 2156
H19                    : CAATTGCTCTGATGAACATTTAAAGAATATTTTGGACCTCAACACTTTGAAGCTTAACATTCTGAACATGCAGGGCAGGCGCGTGCTGCAGCCCCTCGTCAAGA : 2210
H20                    : CAATTGCTCTGATGAACATTTAAAGAATATTTTGGACCTCAACACTTTGAAGCTTAACATTCTGAACATGCAGGGCAGGCGCGTGCTGCAGCCCCTCGTCAAGA : 2192
H21                    : CAATTGCTCTGATGAACATTTAAAGAATATTTTGGACCTCAACACTTTGAAGCTTAACATTCTGAACATGCAGGGCAGGCGCGTGCTGCAGCCCCTCGTCAAGA : 2228
H22                    : CAATTGCTCTGATGAACATTTAAAGAATATTTTGGACCTCAACACTTTGAAGCTTAACATTCTGAACATGCAGGGCAGGCGCGTGCTGCAGCCCCTCGTCAAGA : 2192
H23                    : CAATTGCTCTGATGAACATTTAAAGAATATTTTGGACCTCAACACTTTGAAGCTTAACATTATGAACATGCAGGGCAGGCGCGTGCTGCAGCCCCTCGTCAAGA : 2228
H24                    : CAATTGCTCTGATGAACATTTAAAGAATATTTTGGACCTCAACACTTTGAAGCTTAACATTCTGAACATGCAGGGCAGGCGCGTGCTGCAGCCCCTCGTCAAGA : 2237
H25                    : CAATTGCTCTGATGAACATTTAAAGAATATTTTGGACCTCAACACTTTGAAGCTTAACATTCTGAACATGCAGGGCAGGCGCGTGCTGCAGCCCCTCGTCAAGA : 2192
H26_Mauritania-I_ref   : CAATTGCTCTGATGAACATTTAAAGAATATTTTGGACCTCAACACTTTGAAGCTTAACATTCTGAACATGCAGGGCAGGCGCGTGCTGCAGCCCCTCGTCAAGA : 2282
H27_India-VII_refseq   : CAATTGCTCTGATGAACATTTAAAGAATATTTTGGACCTCAACACTTTGAAGCTTAACATTCTGAACATGCAGGGCAGGCGCGTGCTGCAGCCCCTCGTCAAGA : 2165
H28_Ctg_refsequence    : CAATTGCTCTGATGAACATTTAAAGAATATTTTGGACCTCAACACTTTGAAGCTTAACATTCTGAACATGCAGGGCAGGCGCGTGCTGCAGCCCCTCGTCAAGA : 2237
H29_Salvador-I_refse   : CAATTGCTCTGATGAACATTTAAAGAATATTTTGGACCTCAACACTTTGAAGCTTAACATTCTGAACATGCAGGGCAGGCGCGTGCTGCAGCCCCTCGTCAAGA : 2237
H30_NKorea_refsequen   : CAATTGCTCTGATGAACATTTAAAGAATATTTTGGACCTCAACACTTTGAAGCTTAACATTCTGAACATGCAGGGCAGGCGCGTGCTGCAGCCCCTCGTCAAGA : 2147
H31_P.vivax01_refseq   : CAATTGCTCTGATGAACATTTAAAGAATATTTTGGACCTCAACACTTTGAAGCTTAACATTCTGAACATGCAGGGCAGGCGCGTGCTGCAGCCCCTCGTCAAGA : 2228
H32_Brazil-I_refsequ   : CAATTGCTCTGATGAACATTTAAAGAATATTTTGGACCTCAACACTTTGAAGCTTAACATTCTGAACATGCAGGGCAGGCGCGTGCTGCAGCCCCTCGTCAAGA : 2156
                         CAATTGCTCTGATGAACATTTAAAGAATATTTTGGACCTCAACACTTTGAAGCTTAACATTcTGAACATGCAGGGCAGGCGCGTGCTGCAGCCCCTCGTCAAGA

                         2500       *       2520         *       2540         *       2560         *       2580         *        2600
H1_VCG                 : TGAGCTTCCTCGGCGCCGCGCAGAACCCCGCCCTCAAGGAGATCTGCGAGCCGAGCAACCACCTAGTGGGTGAGTGACGGGGGGGGCGACCTCCATGGGGGGCT : 2260
H2                     : TGAGCTTCCTCGGCGCCGCGCAGAACCCCGCCCTCAAGGAGATCTGCGAGCCGAGCAACCACCTAGTGGGTGAGTGACGGGGGGGGCGACCTCCATGGGGGGCT : 2296
```

```
H3                       : TGAGCTTCCTCGGCGCCGCGCAGAACCCCGCCCTCAAGGAGATCTGCGAGCCGAGCAACCACCTAGTGGGTGAGTGACGGGGGGGGCGACCTCCATGGGGGGCT : 2368
H4                       : TGAGCTTCCTCGGCGCCGCGCAGAACCCCGCCCTCAAGGAGATCTGCGAGCCGAGCAACCACCTAGTGGGTGAGTGACGGGGGGGGCGACCTCCATGGGGGGCT : 2332
H5                       : TGAGCTTCCTCGGCGCCGCGCAGAACCCCGCCCTCAAGGAGATCTGCGAGCCGAGCAACCACCTAGTGGGTGAGTGACGGGGGGGGCGACCTCCATGGGGGGCT : 2332
H6                       : TGAGCTTCCTCGGCGCCGCGCAGAACCCCGCCCTCAAGGAGATCTGCGAGCCGAGCAACCACCTAGTGGGTGAGTGACGGGGGGGGCGACCTCCATGGGGGGCT : 2251
H7                       : TGAGCTTCCTCGGCGCCGCGCAGAACCCCGCCCTCAAGGAGATCTGCGAGCCGAGCAACCACCTAGTGGGTGAGTGACGGGGGGGGCGACCTCCATGGGGGGCT : 2269
H8                       : TGAGCTTCCTCGGCGCCGCGCAGAACCCCGCCCTCAAGGAGATCTGCGAGCCGAGCAACCACCTAGTGGGTGAGTGACGGGGGGGGCGACCTCCATGGGGGGCT : 2305
H9                       : TGAGCTTCCTCGGCGCCGCGCAGAACCCCGCCCTCAAGGAGATCTGCGAGCCGAGCAACCACCTAGTGGGTGAGTGACGGGGGGGGCGACCTCCATGGGGGGCT : 2387
H10                      : TGAGCTTCCTCGGCGCCGCGCAGAACCCCGCCCTCAAGGAGATCTGCGAGCCGAGCAACCACCTAGTGGGTGAGTGACGGGGGGGGCGACCTCCATGGGGGGCT : 2305
H11                      : TGAGCTTCCTCGGCGCCGCGCAGAACCCCGCCCTCAAGGAGATCTGCGAGCCGAGCAACCACCTAGTGGGTGAGTGACGGGGGGGGCGACCTCCATGGGGGGCT : 2314
H12                      : TGAGCTTCCTCGGCGCCGCGCAGAACCCCGCCCTCAAGGAGATCTGCGAGCCGAGCAACCACCTAGTGGGTGAGTGACGGGGGGGGCGACCTCCATGGGGGGCT : 2296
H13                      : TGAGCTTCCTCGGCGCCGCGCAGAACCCCGCCCTCAAGGAGATCTGCGAGCCGAGCAACCACCTAGTGGGTGAGTGACGGGGGGGGCGACCTCCATGGGGGGCT : 2368
H14                      : TGAGCTTCCTCGGCGCCGCGCAGAACCCCGCCCTCAAGGAGATCTGCGAGCCGAGCAACCACCTAGTGGGTGAGTGACGGGGGGGGCGACCTCCATGGGGGGCT : 2314
H15                      : TGAGCTTCCTCGGCGCCGCGCAGAACCCCGCCCTCAAGGAGATCTGCGAGCCGAGCAACCACCTAGTGGGTGAGTGACGGGGGGGGCGACCTCCATGGGGGGCT : 2296
H16                      : TGAGCTTCCTCGGCGCCGCGCAGAACCCCGCCCTCAAGGAGATCTGCGAGCCGAGCAACCACCTAGTGGGTGAGTGACGGGGGGGGCGACCTCCATGGGGGGCT : 2260
H17                      : TGAGCTTCCTCGGCGCCGCGCAGAACCCCGCCCTCAAGGAGATCTGCGAGCCGAGCAACCACCTAGTGGGTGAGTGACGGGGGGGGCGACCTCCATGGGGGGCT : 2476
H18                      : TGAGCTTCCTCGGCGCCGCGCAGAACCCCGCCCTCAAGGAGATCTGCGAGCCGAGCAACCACCTAGTGGGTGAGTGACGGGGGGGGCGACCTCCATGGGGGGCT : 2260
H19                      : TGAGCTTCCTCGGCGCCGCGCAGAACCCCGCCCTCAAGGAGATCTGCGAGCCGAGCAACCACCTAGTGGGTGAGTGACGGGGGGGGCGACCTCCATGGGGGGCT : 2314
H20                      : TGAGCTTCCTCGGCGCCGCGCAGAACCCCGCCCTCAAGGAGATCTGCGAGCCGAGCAACCACCTAGTGGGTGAGTGACGGGGGGGGCGACCTCCATGGGGGGCT : 2296
H21                      : TGAGCTTCCTCGGCGCCGCGCAGAACCCCGCCCTCAAGGAGATCTGCGAGCCGAGCAACCACCTAGTGGGTGAGTGACGGGGGGGGCGACCTCCATGGGGGGCT : 2332
H22                      : TGAGCTTCCTCGGCGCCGCGCAGAACCCCGCCCTCAAGGAGATCTGCGAGCCGAGCAACCACCTAGTGGGTGAGTGACGGGGGGGGCGACCTCCATGGGGGGCT : 2296
H23                      : TGAGCTTCCTCGGCGCCGCGCAGAACCCCGCCCTCAAGGAGATCTGCGAGCCGAGCAACCACCTAGTGGGTGAGTGACGGGGGGGGCGACCTCCATGGGGGGCT : 2332
H24                      : TGAGCTTCCTCGGCGCCGCGCAGAACCCCGCCCTCAAGGAGATCTGCGAGCCGAGCAACCACCTAGTGGGTGAGTGACGGGGGGGGCGACCTCCATGGGGGGCT : 2341
H25                      : TGAGCTTCCTCGGCGCCGCGCAGAACCCCGCCCTCAAGGAGATCTGCGAGCCGAGCAACCACCTAGTGGGTGAGTGACGGGGGGGGCGACCTCCATGGGGGGCT : 2296
H26_Mauritania-I_ref     : TGAGCTTCCTCGGCGCCGCGCAGAACCCCGCCCTCAAGGAGATCTGCGAGCCGAGCAACCACCTAGTGGGTGAGTGACGGGGGGGGCGACCTCCATGGGGGGCT : 2386
H27_India-VII_refseq     : TGAGCTTCCTCGGCGCCGCGCAGAACCCCGCCCTCAAGGAGATCTGCGAGCCGAGCAACCACCTAGTGGGTGAGTGACGGGGGGGGCGACCTCCATGGGGGGCT : 2269
H28_Ctg_refsequence      : TGAGCTTCCTCGGCGCCGCGCAGAACCCCGCCCTCAAGGAGATCTGCGAGCCGAGCAACCACCTAGTGGGTGAGTGACGGGGGGGACGACCTCCATGGGGGGCT : 2341
H29_Salvador-I_refse     : TGAGCTTCCTCGGCGCCGCGCAGAACCCCGCCCTCAAGGAGATCTGCGAGCCGAGCAACCACCTAGTGGGTGAGTGACGGGGGGGACGACCTCCATGGGGGGCT : 2341
H30_NKorea_refsequen     : TGAGCTTCCTCGGCGCCGCGCAGAACCCCGCCCTCAAGGAGATCTGCGAGCCGAGCAACCACCTAGTGGGTGAGTGACGGGGGGGGCGACCTCCATGGGGGGCT : 2251
H31_P.vivax01_refseq     : TGAGCTTCCTCGGCGCCGCGCAGAACCCCGCCCTCAAGGAGATCTGCGAGCCGAGCAACCACCTAGTGGGTGAGTGACGGGGGGGGCGACCTCCATGGGGGGCT : 2332
H32_Brazil-I_refsequ     : TGAGCTTCCTCGGCGCCGCGCAGAACCCCGCCCTCAAGGAGATCTGCGAGCCGAGCAACCACCTAGTGGGTGAGTGACGGGGGGGGCGACCTCCATGGGGGGCT : 2260
                           TGAGCTTCCTCGGCGCCGCGCAGAACCCCGCCCTCAAGGAGATCTGCGAGCCGAGCAACCACCTAGTGGGTGAGTGACGGGGGGGgCGACCTCCATGGGGGGCT

                                    *       2620         *       2640         *       2660         *       2680         *       2700
H1_VCG                   : GCTATTCCAGCGCGGTGATCCACGCAGTGATCCGCGCAGCTGCTTTATTGGAAGCGCTTTACTCGAACTGCCTTCCTTCATTCGAACTTCCTTCCTTCATTCGC : 2364
H2                       : GCTATTCCAGCGCGGTGATCCACGCAGTGATCCGCGCAGCTGCTTTATTGGAAGCGCTTTACTCGAACTGCCTTCCTTCATTCGAACTTCCTTCCTTCATTCGC : 2400
H3                       : GCTATTCCAGCGCGGTGATCCACGCAGTGATCCGCGCAGCTGCTTTATTGGAAGCGCTTTACTCGAACTGCCTTCCTTCATTCGAACTTCCTTCCTTCATTCGC : 2472
H4                       : GCTATTCCAGCGCGGTGATCCACGCAGTGATCCGCGCAGCTGCTTTATTGGAAGCGCTTTACTCGAACTGCCTTCCTTCATTCGAACTTCCTTCCTTCATTCGC : 2436
H5                       : GCTATTCCAGCGCGGTGATCCACGCAGTGATCCGCGCAGCTGCTTTATTGGAAGCGCTTTACTCGAACTGCCTTCCTTCATTCGAACTTCCTTCCTTCATTCGC : 2436
H6                       : GCTATTCCAGCGCGGTGATCCACGCAGTGATCCGCGCAGCTGCTTTATTGGAAGCGCTTTACTCGAACTGCCTTCCTTCATTCGAACTTCCTTCCTTCATTCGC : 2355
H7                       : GCTATTCCAGCGCGGTGATCCACGCAGTGATCCGCGCAGCTGCTTTATTGGAAGCGCTTTACTCGAACTGCCTTCCTTCATTCGAACTTCCTTCCTTCATTCGC : 2373
H8                       : GCTATTCCAGCGCGGTGATCCACGCAGTGATCCGCGCAGCTGCTTTATTGGAAGCGCTTTACTCGAACTGCCTTCCTTCATTCGAACTTCCTTCCTTCATTCGC : 2409
H9                       : GCTATTCCAGCGCGGTGATCCACGCAGTGATCCGCGCAGCTGCTTTATTGGAAGCGCTTTACTCGAACTGCCTTCCTTCATTCGAACTTCCTTCCTTCATTCGC : 2491
H10                      : GCTATTCCAGCGCGGTGATCCACGCAGTGATCCGCGCAGCTGCTTTATTGGAAGCGCTTTACTCGAACTGCCTTCCTTCATTCGAACTTCCTTCCTTCATTCGC : 2409
H11                      : GCTATTCCAGCGCGGTGATCCACGCAGTGATCCGCGCAGCTGCTTTATTGGAAGCGCTTTACTCGAACTGCCTTCCTTCATTCGAACTTCCTTCCTTCATTCGC : 2418
H12                      : GCTATTCCAGCGCGGTGATCCACGCAGTGATCCGCGCAGCTGCTTTATTGGAAGCGCTTTACTCGAACTGCCTTCCTTCATTCGAACTTCCTTCCTTCATTCGC : 2400
H13                      : GCTATTCCAGCGCGGTGATCCACGCAGTGATCCGCGCAGCTGCTTTATTGGAAGCGCTTTACTCGAACTGCCTTCCTTCATTCGAACTTCCTTCCTTCATTCGC : 2472
H14                      : GCTATTCCAGCGCGGTGATCCACGCAGTGATCCGCGCAGCTGCTTTATTGGAAGCGCTTTACTCGAACTGCCTTCCTTCATTCGAACTTCCTTCCTTCATTCGC : 2418
H15                      : GCTATTCCAGCGCGGTGATCCACGCAGTGATCCGCGCAGCTGCTTTATTGGAAGCGCTTTACTCGAACTGCCTTCCTTCATTCGAACTTCCTTCCTTCATTCGC : 2400
H16                      : GCTATTCCAGCGCGGTGATCCACGCAGTGATCCGCGCAGCTGCTTTATTGGAAGCGCTTTACTCGAACTGCCTTCCTTCATTCGAACTTCCTTCCTTCATTCGC : 2364
H17                      : GCTATTCCAGCGCGGTGATCCACGCAGTGATCCGCGCAGCTGCTTTATTGGAAGCGCTTTACTCGAACTGCCTTCCTTCATTCGAACTTCCTTCCTTCATTCGC : 2580
```

```
H18                       : GCTATTCCAGCGCGGTGATCCACGCAGTGATCCGCGCAGCTGCTTTATTGGAAGCGCTTTACTCGAACTGCCTTCCTTCATTCGAACTTCCTTCCTTCATTCGC : 2364
H19                       : GCTATTCCAGCGCGGTGATCCACGCAGTGATCCGCGCAGCTGCTTTATTGGAAGCGCTTTACTCGAACTGCCTTCCTTCATTCGAACTTCCTTCCTTCATTCGC : 2418
H20                       : GCTATTCCAGCGCGGTGATCCACGCAGTGATCCGCGCAGCTGCTTTATTGGAAGCGCTTTACTCGAACTGCCTTCCTTCATTCGAACTTCCTTCCTTCATTCGC : 2400
H21                       : GCTATTCCAGCGCGGTGATCCACGCAGTGATCCGCGCAGCTGCTTTATTGGAAGCGCTTTACTCGAACTGCCTTCCTTCATTCGAACTTCCTTCCTTCATTCGC : 2436
H22                       : GCTATTCCAGCGCGGTGATCCACGCAGTGATCCGCGCAGCTGCTTTATTGGAAGCGCTTTACTCGAACTGCCTTCCTTCATTCGAACTTCCTTCCTTCATTCGC : 2400
H23                       : GCTATTCCAGCGCGGTGATCCACGCAGTGATCCGCGCAGCTGCTTTATTGGAAGCGCTTTACTCGAACTGCCTTCCTTCATTCGAACTTCCTTCCTTCATTCGC : 2436
H24                       : GCTATTCCAGCGCGGTGATCCACGCAGTGATCCGCGCAGCTGCTTTATTGGAAGCGCTTTACTCGAACTGCCTTCCTTCATTCGAACTTCCTTCCTTCATTCGC : 2445
H25                       : GCTATTCCAGCGCGGTGATCCACGCAGTGATCCGCGCAGCTGCTTTATTGGAAGCGCTTTACTCGAACTGCCTTCCTTCATTCGAACTTCCTTCCTTCATTCGC : 2400
H26_Mauritania-I_ref      : GCTATTCCAGCGCGGTGATCCACGCAGTGATCCGCGCAGCTGCTTTATTGGAAGCGCTTTACTCGAACTGCCTTCCTTCATTCGAACTTCCTTCCTTCATTCGC : 2490
H27_India-VII_refseq      : GCTATTCCAGCGCGGTGATCCACGCAGTGATCCGCGCAGCTGCTTTATTGGAAGCGCTTTACTCGAACTGCCTTCCTTCATTCGAACTTCCTTCCTTCATTCGC : 2373
H28_Ctg_refsequence       : GCTATTCCAGCGCGGTGATCCACGCAGTGATCCGCGCAGCTGCTTTATTGGAAGCGCTTTACTCGAACTGCCTTCCTTCATTCGAACTTCCTTCCTTCATTCGC : 2445
H29_Salvador-I_refse      : GCTATTCCAGCGCGGTGATCCACGCAGTGATCCGCGCAGCTGCTTTATTGGAAGCGCTTTACTCGAACTGCCTTCCTTCATTCGAACTTCCTTCCTTCATTCGC : 2445
H30_NKorea_refsequen      : GCTATTCCAGCGCGGTGATCCACGCAGTGATCCGCGCAGCTGCTTTATTGGAAGCGCTTTACTCGAACTGCCTTCCTTCATTCGAACTTCCTTCCTTCATTCGC : 2355
H31_P.vivax01_refseq      : GCTATTCCAGCGCGGTGATCCACGCAGTGATCCGCGCAGCTGCTTTATTGGAAGCGCTTTACTCGAACTGCCTTCCTTCATTCGAACTTCCTTCCTTCATTCGC : 2436
H32_Brazil-I_refsequ      : GCTATTCCAGCGCGGTGATCCACGCAGTGATCCGCGCAGCTGCTTTATTGGAAGCGCTTTACTCGAACTGCCTTCCTTCATTCGAACTTCCTTCCTTCATTCGC : 2364
                            GCTATTCCAGCGCGGTGATCCACGCAGTGATCCGCGCAGCTGCTTTATTGGAAGCGCTTTACTCGAACTGCCTTCCTTCATTCGAACTTCCTTCCTTCATTCGC

                                  *      2720         *      2740         *      2760         *      2780         *      2800
H1_VCG                    : ACCGCCTTCTTTCACCCCCCTCAGACGAGACCCTCTCCAAGCTGCTCAACCTGCTCTCGACCGGTTCGCACGAACTCCTCGCGGCCGAGGTGGAAAAGCGCGGC : 2468
H2                        : ACCGCCTTCTTTCACCCCCCTCAGACGAGACCCTCTCCAAGCTGCTCAACCTGCTCTCGACCGGTTCGCACGAACTCCTCGCGGCCGAGGTGGAAAAGCGCGGC : 2504
H3                        : ACCGCCTTCTTTCACCCCCCTCAGACGAGACCCTCTCCAAGCTGCTCAACCTGCTCTCGACCGGTTCGCACGAACTCCTCGCGGCCGAGGTGGAAAAGCGCGGC : 2576
H4                        : ACCGCCTTCTTTCACCCCCCTCAGACGAGACCCTCTCCAAGCTGCTCAACCTGCTCTCGACCGGTTCGCACGAACTCCTCGCGGCCGAGGTGGAAAAGCGCGGC : 2540
H5                        : ACCGCCTTCTTTCACCCCCCTCAGACGAGACCCTCTCCAAGCTGCTCAACCTGCTCTCGACCGGTTCGCACGAACTCCTCGCGGCCGAGGTGGAAAAGCGCGGC : 2540
H6                        : ACCGCCTTCTTTCACCCCCCTCAGACGAGACTCTCTCCAAGCTGCTCAACCTGCTCTCGACCGGTTCGCACGAACTCCTCGCGGCCGAGGTGGAAAAGCGCGGC : 2459
H7                        : ACCGCCTTCTTTCACCCCCCTCAGACGAGACCCTCTCCAAGCTGCTCAACCTGCTCTCGACCGGTTCGCACGAACTCCTCGCGGCCGAGGTGGAAAAGCGCGGC : 2477
H8                        : ACCGCCTTCTTTCACCCCCCTCAGACGAGACCCTCTCCAAGCTGCTCAACCTGCTCTCGACCGGTTCGCACGAACTCCTCGCGGCCGAGGTGGAAAAGCGCGGC : 2513
H9                        : ACCGCCTTCTTTCACCCCCCTCAGACGAGACCCTCTCCAAGCTGCTCAACCTGCTCTCGACCGGTTCGCACGAACTCCTCGCGGCCGAGGTGGAAAAGCGCGGC : 2595
H10                       : ACCGCCTTCTTTCACCCCCCTCAGACGAGACCCTCTCCAAGCTGCTCAACCTGCTCTCGACCGGTTCGCACGAACTCCTCGCGGCCGAGGTGGAAAAGCGCGGC : 2513
H11                       : ACCGCCTTCTTTCACCCCCCTCAGACGAGACCCTCTCCAAGCTGCTCAACCTGCTCTCGACCGGTTCGCACGAACTCCTCGCGGCCGAGGTGGAAAAGCGCGGC : 2522
H12                       : ACCGCCTTCTTTCACCCCCCTCAGACGAGACCCTCTCCAAGCTGCTCAACCTGCTCTCGACCGGTTCGCACGAACTCCTCGCGGCCGAGGTGGAAAAGCGCGGC : 2504
H13                       : ACCGCCTTCTTTCACCCCCCTCAGACGAGACCCTCTCCAAGCTGCTCAACCTGCTCTCGACCGGTTCGCACGAACTCCTCGCGGCCGAGGTGGAAAAGCGCGGC : 2576
H14                       : ACCGCCTTCTTTCACCCCCCTCAGACGAGACCCTCTCCAAGCTGCTCAACCTGCTCTCGACCGGTTCGCACGAACTCCTCGCGGCCGAGGTGGAAAAGCGCGGC : 2522
H15                       : ACCGCCTTCTTTCACCCCCCTCAGACGAGACCCTCTCCAAGCTGCTCAACCTGCTCTCGACCGGTTCGCACGAACTCCTCGCGGCCGAGGTGGAAAAGCGCGGC : 2504
H16                       : ACCGCCTTCTTTCACCCCCCTCAGACGAGACCCTCTCCAAGCTGCTCAACCTGCTCTCGACCGGTTCGCACGAACTCCTCGCGGCCGAGGTGGAAAAGCGCGGC : 2468
H17                       : ACCGCCTTCTTTCACCCCCCTCAGACGAGACCCTCTCCAAGCTGCTCAACCTGCTCTCGACCGGTTCGCACGAACTCCTCGCGGCCGAGGTGGAAAAGCGCGGC : 2684
H18                       : ACCGCCTTCTTTCACCCCCCTCAGACGAGACCCTCTCCAAGCTGCTCAACCTGCTCTCGACCGGTTCGCACGAACTCCTCGCGGCCGAGGTGGAAAAGCGCGGC : 2468
H19                       : ACCGCCTTCTTTCACCCCCCTCAGACGAGACCCTCTCCAAGCTGCTCAACCTGCTCTCGACCGGTTCGCACGAACTCCTCGCGGCCGAGGTGGAAAAGCGCGGC : 2522
H20                       : ACCGCCTTCTTTCACCCCCCTCAGACGAGACTCTCTCCAAGCTGCTCAACCTGCTCTCGACCGGTTCGCACGAACTCCTCGCGGCCGAGGTGGAAAAGCGCGGC : 2504
H21                       : ACCGCCTTCTTTCACCCCCCTCAGACGAGACTCTCTCCAAGCTGCTCAACCTGCTCTCGACCGGTTCGCACGAACTCCTCGCGGCCGAGGTGGAAAAGCGCGGC : 2540
H22                       : ACCGCCTTCTTTCACCCCCCTCAGACGAGACTCTCTCCAAGCTGCTCAACCTGCTCTCGACCGGTTCGCACGAACTCCTCGCGGCCGAGGTGGAAAAGCGCGGC : 2504
H23                       : ACCGCCTTCTTTCACCCCCCTCAGACGAGACCCTCTCCAAGCTGCTCAACCTGCTCTCGACCGGTTCGCACGAACTCCTCGCGGCCGAGGTGGAAAAGCGCGGC : 2540
H24                       : ACCGCCTTCTTTCACCCCCCTCAGACGAGACCCTCTCCAAGCTGCTCAACCTGCTCTCGACCGGTTCGCACGAACTCCTCGCGGCCGAGGTGGAAAAGCGCGGC : 2549
H25                       : ACCGCCTTCTTTCACCCCCCTCAGACGAGACCCTCTCCAAGCTGCTCAACCTGCTCTCGACCGGTTCGCACGAACTCCTCGCGGCCGAGGTGGAAAAGCGCGGC : 2504
H26_Mauritania-I_ref      : ACCGCCTTCTTTCACCCCCCTCAGACGAGACCCTCTCCAAGCTGCTCAACCTGCTCTCGACCGGTTCGCACGAACTCCTCGCGGCCGAGGTGGAAAAGCGCGGC : 2594
H27_India-VII_refseq      : ACCGCCTTCTTTCACCCCCCTCAGACGAGACCCTCTCCAAGCTGCTCAACCTGCTCTCGACCGGTTCGCACGAACTCCTCGCGGCCGAGGTGGAAAAGCGCGGC : 2477
H28_Ctg_refsequence       : ACCGCCTTCTTTCACCCCCCTCAGACGAGACCCTCTCCAAGCTGCTCAACCTGCTCTCGACCGGTTCGCACGAACTCCTCGCGGCCGAGGTGGAAAAGCGCGGC : 2549
H29_Salvador-I_refse      : ACCGCCTTCTTTCACCCCCCTCAGACGAGACCCTCTCCAAGCTGCTCAACCTGCTCTCGACCGGTTCGCACGAACTCCTCGCGGCCGAGGTGGAAAAGCGCGGC : 2549
H30_NKorea_refsequen      : ACCGCCTTCTTTCACCCCCCTCAGACGAGACCGTCTCCAAGCTGCTCAACCTGCTCTCGACCGGTTCGCACGAACTCCTCGCGGCCGAGGTGGAAAAGCGCGGC : 2459
H31_P.vivax01_refseq      : ACCGCCTTCTTTCACCCCCCTCAGACGAGACCCTCTCCAAGCTGCTCAACCTGCTCTCGACCGGTTCGCACGAACTCCTCGCGGCCGAGGTGGAAAAGCGCGGC : 2540
H32_Brazil-I_refsequ      : ACCGCCTTCTTTCACCCCCCTCAGACGAGACCCTCTCCAAGCTGCTCAACCTGCTCTCGACCGGTTCGCACGAACTCCTCGCGGCCGAGGTGGAAAAGCGCGGC : 2468
```

```
                              ACCGCCTTCTTTCACCCCCCTCAGACGAGACccTCTCCAAGCTGCTCAACCTGCTCTCGACCGGTTCGCACGAACTCCTCGCGGCCGAGGTGGAAAAGCGCGGC

                                *       2820         *       2840         *       2860         *       2880         *       2900         *
H1_VCG                      : TTCGACGAGGACTACATCCAGGAGGAAATCAAAAACATAAACGAAAGCGACAACAACGTTCGGGACAAGGGCGAAGACGAAGTGGAAAATCTCATTTTTGAAGA : 2572
H2                          : TTCGACGAGGACTACATCCAGGAGGAAATCAAAAACATAAACGAAAGCGACAACAACGTTCGGGACAAGGGCGAAGACGAAGTGGAAAATCTCATTTTTGAAGA : 2608
H3                          : TTCGACGAGGACTACATCCAGGAGGAAATCAAAAACATAAACGAAAGCGACAACAACGTTCGGGACAAGGGCGAAGACGAAGTGGAAAATCTCATTTTTGAAGA : 2680
H4                          : TTCGACGAGGACTACATCCAGGAGGAAATCAAAAACATAAACGAAAGCGACAACAACGTTCGGGACAAGGGCGAAGACGAAGTGGAAAATCTCATTTTTGAAGA : 2644
H5                          : TTCGACGAGGACTACATCCAGGAGGAAATCAAAAACATAAACGAAAGCGACAACAACGTTCGGGACAAGGGCGAAGACGAAGTGGAAAATCTCATTTTTGAAGA : 2644
H6                          : TTCGACGAGGACTACATCCAGGAGGAAATCAAAAACATAAACGAAAGCGACAACAACGTTCGGGACAAGGGCGAAGACGAAGTGGAAAATCTCATTTTTGAAGA : 2563
H7                          : TTCGACGAGGACTACATCCAGGAGGAAATCAAAAACATAAACGAAAGCGACAACAACGTTCGGGACAAGGGCGAAGACGAAGTGGAAAATCTCATTTTTGAAGA : 2581
H8                          : TTCGACGAGGACTACATCCAGGAGGAAATCAAAAACATAAACGAAAGCGACAACAACGTTCGGGACAAGGGCGAAGACGAAGTGGAAAATCTCATTTTTGAAGA : 2617
H9                          : TTCGACGAGGACTACATCCAGGAGGAAATCAAAAACATAAACGAAAGCGACAACAACGTTCGGGACAAGGGCGAAGACGAAGTGGAAAATCTCATTTTTGAAGA : 2699
H10                         : TTCGACGAGGACTACATCCAGGAGGAAATCAAAAACATAAACGAAAGCGACAACAACGTTCGGGACAAGGGCGAAGACGAAGTGGAAAATCTCATTTTTGAAGA : 2617
H11                         : TTCGACGAGGACTACATCCAGGAGGAAATCAAAAACATAAACGAAAGCGACAACAACGTTCGGGACAAGGGCGAAGACGAAGTGGAAAATCTCATTTTTGAAGA : 2626
H12                         : TTCGACGAGGACTACATCCAGGAGGAAATCAAAAACATAAACGAAAGCGACAACAACGTTCGGGACAAGGGCGAAGACGAAGTGGAAAATCTCATTTTTGAAGA : 2608
H13                         : TTCGACGAGGACTACATCCAGGAGGAAATCAAAAACATAAACGAAAGCGACAACAACGTTCGGGACAAGGGCGAAGACGAAGTGGAAAATCTCATTTTTGAAGA : 2680
H14                         : TTCGACGAGGACTACATCCAGGAGGAAATCAAAAACATAAACGAAAGCGACAACAACGTTCGGGACAAGGGCGAAGACGAAGTGGAAAATCTCATTTTTGAAGA : 2626
H15                         : TTCGACGAGGACTACATCCAGGAGGAAATCAAAAACATAAACGAAAGCGACAACAACGTTCGGGACAAGGGCGAAGACGAAGTGGAAAATCTCATTTTTGAAGA : 2608
H16                         : TTCGACGAGGACTACATCCAGGAGGAAATCAAAAACATAAACGAAAGCGACAACAACGTTCGGGACAAGGGCGAAGACGAAGTGGAAAATCTCATTTTTGAAGA : 2572
H17                         : TTCGACGAGGACTACATCCAGGAGGAAATCAAAAACATAAACGAAAGCGACAACAACGTTCGGGACAAGGGCGAAGACGAAGTGGAAAATCTCATTTTTGAAGA : 2788
H18                         : TTCGACGAGGACTACATCCAGGAGGAAATCAAAAACATAAACGAAAGCGACAACAACGTTCGGGACAAGGGCGAAGACGAAGTGGAAAATCTCATTTTTGAAGA : 2572
H19                         : TTCGACGAGGACTACATCCAGGAGGAAATCAAAAACATAAACGAAAGCGACAACAACGTTCGGGACAAGGGCGAAGACGAAGTGGAAAATCTCATTTTTGAAGA : 2626
H20                         : TTCGACGAGGACTACATCCAGGAGGAAATCAAAAACATAAACGAAAGCGACAACAACGTTCGGGACAAGGGCGAAGACGAAGTGGAAAATCTCATTTTTGAAGA : 2608
H21                         : TTCGACGAGGACTACATCCAGGAGGAAATCAAAAACATAAACGAAAGCGACAACAACGTTCGGGACAAGGGCGAAGACGAAGTGGAAAATCTCATTTTTGAAGA : 2644
H22                         : TTCGACGAGGACTACATCCAGGAGGAAATCAAAAACATAAACGAAAGCGACAACAACGTTCGGGACAAGGGCGAAGACGAAGTGGAAAATCTCATTTTTGAAGA : 2608
H23                         : TTCGACGAGGACTACATCCAGGAGGAAATCAAAAACATAAACGAAAGCGACAACAACGTTCGGGACAAGGGCGAAGACGAAGTGGAAAATCTCATTTTTGAAGA : 2644
H24                         : TTCGACGAGGACTACATCCAGGAGGAAATCAAAAACATAAACGAAAGCGACAACAACGTTCGGGACAAGGGCGAAGACGAAGTGGAAAATCTCATTTTTGAAGA : 2653
H25                         : TTCGACGAGGACTACATCCAGGAGGAAATCAAAAACATAAACGAAAGCGACAACAACGTTCGGGACAAGGGCGAAGACGAAGTGGAAAATCTCATTTTTGAAGA : 2608
H26_Mauritania-I_ref        : TTCGACGAGGACTACATCCAGGAGGAAATCAAAAACATAAACGAAAGCGACAACAACGTTCGGGACAAGGGCGAAGACGAAGTGGAAAATCTCATTTTTGAAGA : 2698
H27_India-VII_refseq        : TTCGACGAGGACTACATCCAGGAGGAAATCAAAAACATAAACGAAAGCGACAACAACGTTCGGGACAAGGGCGAAGACGAAGTGGAAAATCTCATTTTTGAAGA : 2581
H28_Ctg_refsequence         : TTCGACGAGGACTACATCCAGGAGGAAATCAAAAACATAAACGAAAGCGACAACAACGTTCGGGACAAGGGCGAAGACGAAGTGGAAAATCTCATTTTTGAAGA : 2653
H29_Salvador-I_refse        : TTCGACGAGGACTACATCCAGGAGGAAATCAAAAACATAAACGAAAGCGACAACAACGTTCGGGACAAGGGCGAAGACGAAGTGGAAAATCTCATTTTTGAAGA : 2653
H30_NKorea_refsequen        : TTCGACGAGGACTACATCCAGGAGGAAATCAAAAACATAAACGAAAGCGACAACAACGTTCGGGACAAGGGCGAAGACGAAGTGGAAAATCTCATTTTTGAAGA : 2563
H31_P.vivax01_refseq        : TTCGACGAGGACTACATCCAGGAGGAAATCAAAAACATAAACGAAAGCGACAACAACGTTCGGGACAAGGGCGAAGACGAAGTGGAAAATCTCATTTTTGAAGA : 2644
H32_Brazil-I_refsequ        : TTCGACGAGGACTACATCCAGGAGGAAATCAAAAACATAAACGAAAGCGACAACAACGTTCGGGACAAGGGCGAAGACGAAGTGGAAAATCTCATTTTTGAAGA : 2572
                              TTCGACGAGGACTACATCCAGGAGGAAATCAAAAACATAAACGAAAGCGACAACAACGTTCGGGACAAGGGCGAAGACGAAGTGGAAAATCTCATTTTTGAAGA


H1_VCG                      : CTTGTGA : 2579
H2                          : CTTGTGA : 2615
H3                          : CTTGTGA : 2687
H4                          : CTTGTGA : 2651
H5                          : CTTGTGA : 2651
H6                          : CTTGTGA : 2570
H7                          : CTTGTGA : 2588
H8                          : CTTGTGA : 2624
H9                          : CTTGTGA : 2706
H10                         : CTTGTGA : 2624
H11                         : CTTGTGA : 2633
H12                         : CTTGTGA : 2615
```

```
H13                  : CTTGTGA : 2687
H14                  : CTTGTGA : 2633
H15                  : CTTGTGA : 2615
H16                  : CTTGTGA : 2579
H17                  : CTTGTGA : 2795
H18                  : CTTGTGA : 2579
H19                  : CTTGTGA : 2633
H20                  : CTTGTGA : 2615
H21                  : CTTGTGA : 2651
H22                  : CTTGTGA : 2615
H23                  : CTTGTGA : 2651
H24                  : CTTGTGA : 2660
H25                  : CTTGTGA : 2615
H26_Mauritania-I_ref : CTTGTGA : 2705
H27_India-VII_refseq : CTTGTGA : 2588
H28_Ctg_refsequence  : CTTGTGA : 2660
H29_Salvador-I_refse : CTTGTGA : 2660
H30_NKorea_refsequen : CTTGTGA : 2570
H31_P.vivax01_refseq : CTTGTGA : 2651
H32_Brazil-I_refsequ : CTTGTGA : 2579
                       CTTGTGA
```
