# Supplementary figures and images for: Size polymorphism and low sequence diversity in the locus encoding the Plasmodium vivax rhoptry neck protein 4 (PvRON4) in Colombian isolates
Source: Malar J. 2016 Oct 18;15:501. doi: 10.1186/s12936-016-1563-4 (PMC5069803; doi:10.1186/s12936-016-1563-4)

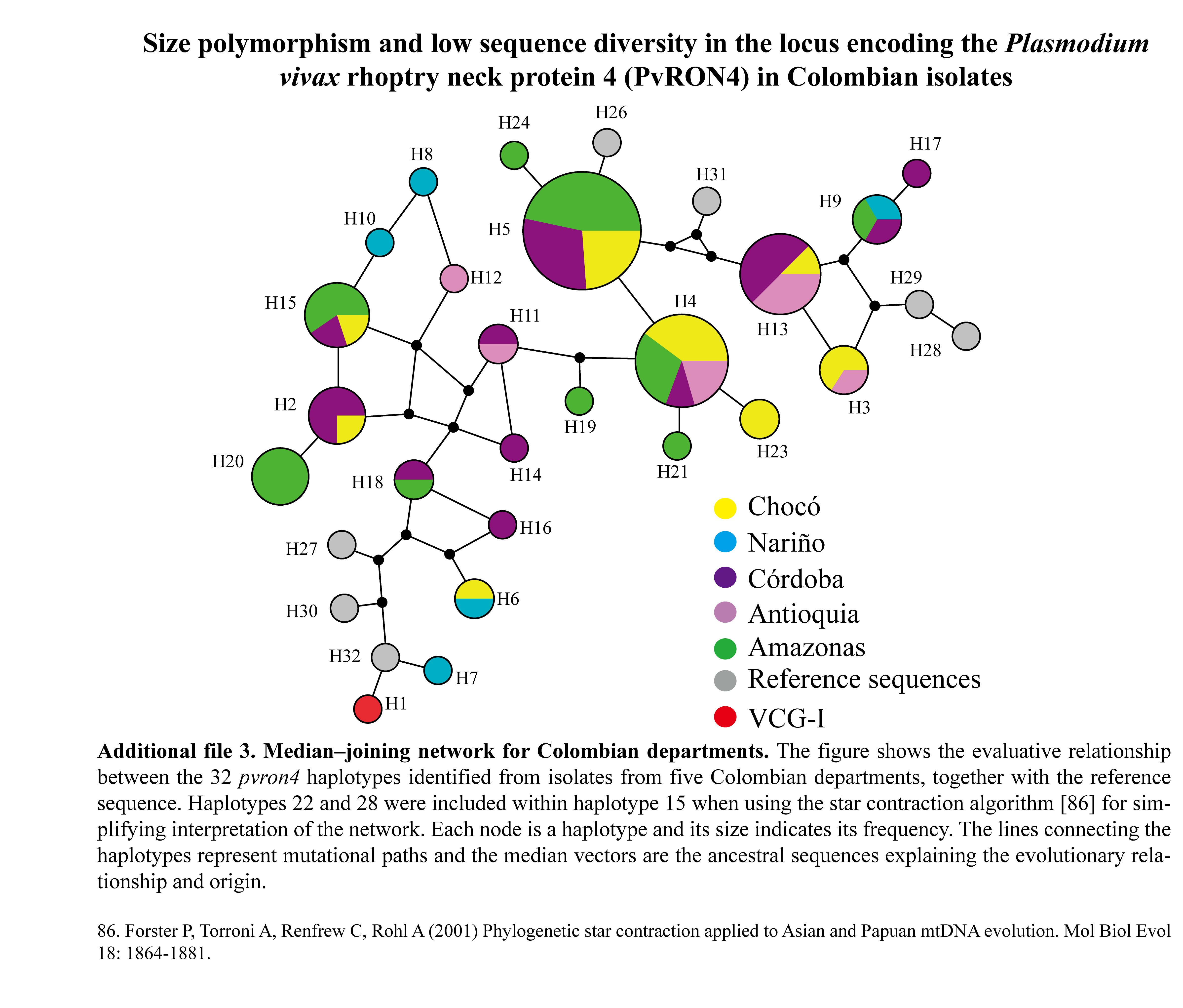

Supplement: Supplementary file 3 — Additional file 3. Median–joining network for Colombian departments. The Figure shows the evaluative relationship between the 32 pvron4 haplotypes identified from isolates from five Colombian departments, together with the reference sequence. Haplotypes 22 and 28 were included within haplotype 15 when using the star contraction algorithm [86] for simplifying interpretation of the network. Each node is a haplotype and its size indicates its frequency. The lines connecting the haplotypes represent mutational paths and the median vectors are the ancestral sequences explaining the evolutionary relationship and origin. [file 12936_2016_1563_MOESM3_ESM.tif]

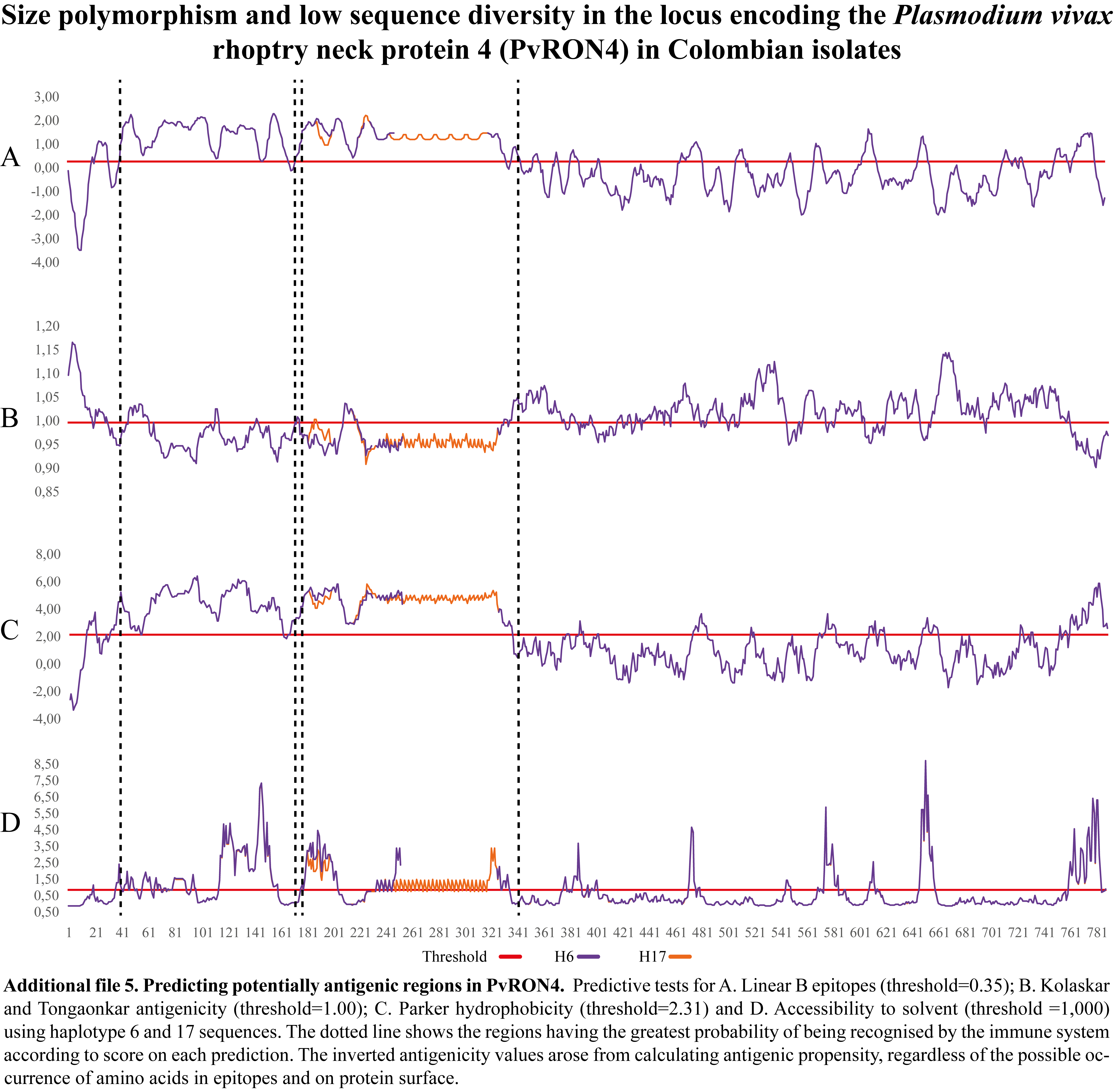

Supplement: Supplementary file 5 — Additional file 5. Predicting potentially antigenic regions in PvRON4. Predictive tests for A. Linear B epitopes (threshold=0.35); B. Kolaskar and Tongaonkar antigenicity (threshold=1.00); C. Parker hydrophobicity (threshold=2.31) and D. Accessibility to solvent (threshold=1,000) using haplotype 6 and 17 sequences. The dotted line shows the regions having the greatest probability of being recognised by the immune system according to score on each prediction. The inverted antigenicity values arose from calculating antigenic propensity, regardless of the possible occurrence of amino acids in epitopes and on protein surface. [file 12936_2016_1563_MOESM5_ESM.tif]
