# Supplementary material for: Size polymorphism and low sequence diversity in the locus encoding the Plasmodium vivax rhoptry neck protein 4 (PvRON4) in Colombian isolates
Source: Malar J. 2016 Oct 18;15:501. doi: 10.1186/s12936-016-1563-4 (PMC5069803; doi:10.1186/s12936-016-1563-4)
Supplement: Supplementary file 4 — Additional file 4. Inter-population FST statistic for pvron4 per department. FST was calculated for parasite subpopulations in Colombia. Values close to 0 indicate low genetic differentiation while values close to 1 indicate high genetic differentiation. Values below the diagonal indicate the FST value and those above the diagonal represent the p-values. Values in bold indicate significant differences having p <0.03. [file 12936_2016_1563_MOESM4_ESM.pdf]

Size polymorphism and low sequence diversity in the locus encoding the *Plasmodium vivax* rhoptry neck protein 4 (PvRON4) in Colombian isolates

	**Chocó**	**Nariño**	**Antioquia**	**Córdoba**	**Amazonas**
**Chocó**		0.00195	0.22656	0.19336	0.05176
**Nariño**	**0.40062**		0.02930	0.05273	0.00879
**Antioquia**	0.04272	**0.28000**		0.43555	0.01172
**Córdoba**	0.02428	0.13422	-0.01859		0.07715
**Amazonas**	0.09516	**0.27552**	**0.22928**	0.05302	

Additional file 4. Inter-population F_{ST} statistic for *pvron4* per department F_{ST} was calculated for parasite subpopulations in Colombia. Values close to 0 indicate low genetic differentiation whilst values close to 1 indicate high genetic differentiation. Values below the diagonal indicate the F_{ST} value and those above the diagonal represent the p-values. Values in bold indicate significant differences having $p < 0.03$.
